# Hepatoprotective Principles and Other Chemical Constituents from the Mycelium of *Phellinus linteus*

**DOI:** 10.3390/molecules23071705

**Published:** 2018-07-12

**Authors:** Shiow-Chyn Huang, Pei-Wen Wang, Ping-Chung Kuo, Hsin-Yi Hung, Tai-Long Pan

**Affiliations:** 1 Department of Pharmacy, Chia-Nan University of Pharmacy and Science, Tainan 717, Taiwan; 2 Department of Medical Research, China Medical University Hospital, China Medical University, Taichung 404, Taiwan; pwwang5105@hotmail.com; 3 School of Pharmacy, College of Medicine, National Cheng Kung University, Tainan 701, Taiwan; z10502016@email.ncku.edu.tw (P.-C.K.); z10308005@email.ncku.edu.tw (H.-Y.H.); 4 School of Traditional Chinese Medicine, Chang Gung University; Research Center for Chinese Herbal Medicine and Research Center for Food and Cosmetic Safety, College of Human Ecology, Chang Gung University of Science and Technology; Liver Research Center, Chang Gung Memorial Hospital, Taoyuan 333, Taiwan; pan@mail.cgu.edu.tw

**Keywords:** *Phellinus linteus*, hepatic fibrosis, ionone derivative, spectroscopic and spectrometric analysis, hepatic stellate cell

## Abstract

In the dimethylnitrosamine (DMN)-induced hepatic fibrosis Wistar rat model, the mycelium extract of *Phellinus linteus* (PLE) (20 mg/Kg) displayed significant protection against hepatic fibrosis. The present investigation characterized eleven new ionone derivatives, phellinulins D–N (**4**–**14**), from the *P. linteus* mycelium extract and the relative stereochemical structures were constructed according to the spectroscopic and spectrometric analytical results. Some purified compounds were examined for their inhibitory effects on activated rat hepatic stellate cells (HSCs) and several isolates did exhibit significant protection. The results indicated that the mycelium of *P. linteus* could be explored as a hepatoprotective drug or healthy food candidate in the near future.

## 1. Introduction

Medicinal fungi could produce various bioactive metabolites including antibiotics [1], anti-cancer drugs [2,3] and immunosuppressants [4,5]. Many medically significant metabolites had been reported from various edible fungi. Recent research results indicated that Sangwhang (*Phellinus linteus*) displayed the hepatoprotective and antihepatotoxic effects [6,7,8,9,10]. Liver fibrosis, resulted from hepatitis, alcoholic liver diseases, or biliary diseases, will lead to serious irreversible cirrhosis and liver damage [11,12]. Traditionally, *P. linteus* (Hymenochaetaceae) was commonly used as medicines or healthy foods in oriental countries to treat several healthy problems such as ulcer, diabetes, cancer and infections. In the previous reports, *P. linteus* exhibited wide spectrum of bioactivities, such as anti-platelet aggregation, anti-diabetic, anti-dementia, antioxidant, anti-inflammatory, cytotoxic and anti-viral effects [13,14,15,16,17,18]. In our previous paper, the mycelium ethanol extract of *P. linteus* and purified compound phellinulin A (**1**) displayed significant protection against hepatic fibrosis [19]. Although various compounds had been identified from the cultured mycelium of *P. linteus* and reported significant antioxidant and anti-inflammatory activities [20,21,22,23,24,25,26], there were no comprehensive investigations regarding the bioactive compounds to inhibit hepatic fibrosis. Moreover, the DMN-induced hepatic fibrosis Wistar rat model [27] shows similar manifestations as progression in human liver fibrosis and thus this model is chosen to explore the protective effects of isolated principles from PLE against hepatic fibrosis. The chemical compositions of the cultured mycelium were thoroughly studied. Several isolated compounds from PLE along with the crude extract were examined for their inhibitory potentials of activated HSCs to discover natural hepatoprotective lead compounds.

## 2. Results

**Figure 1:**
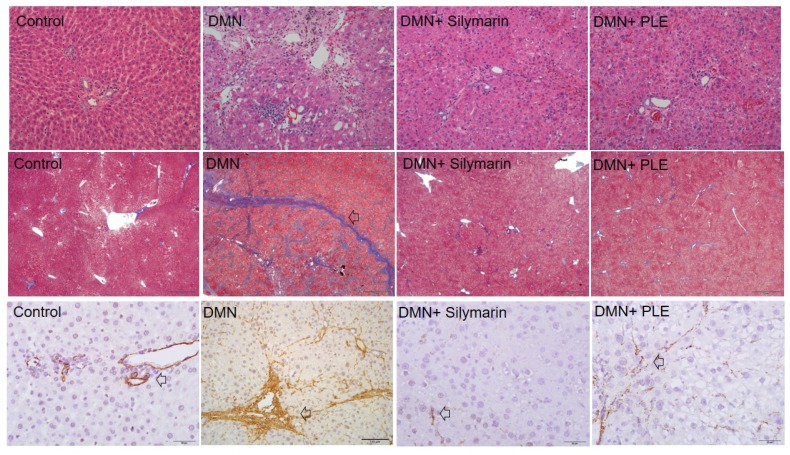
Histological changes in rat liver tissues of PLE.

**Figure 2:**
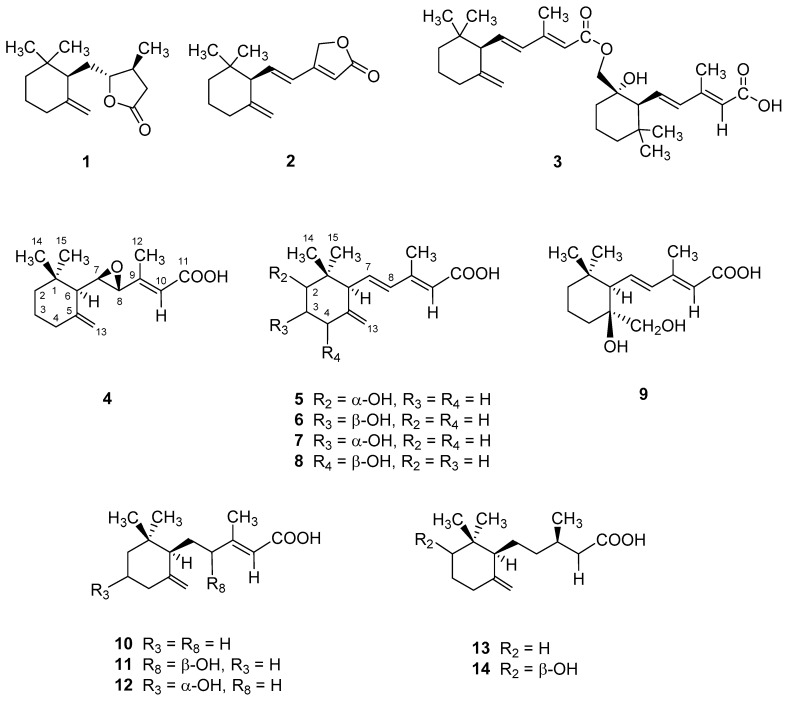
Structures of compounds **1**–**14**.

The DMN-induced hepatic fibrosis Wistar rat model treated with PLE was utilized to investigate the protective efficacy of PLE against hepatic fibrosis in vivo. The histological images (Figure 1) indicated that intact lobular architecture and rare expression of collagen were shown in the normal control, while DMN administration caused severe hepatic injury demonstrated as massive necrosis of hepatocytes, lymphocyte infiltration (indicated by red arrow) as well as increased collagen synthesis (indicated by black arrow). On the other hand, both the positive control exposed to silymarin and PLE treatment could effectively attenuate the liver cell damage, fibrosis and collage accumulation induced by DMN application. These findings suggest that PLE could significantly prevent the hepatic detriment and fibrogenesis caused by chemical toxicity such as DMN. Successively, the powder of dried *P. linteus* mycelium was refluxed with ethanol and the afforded syrup was extracted with chloroform to yield CHCl_3_ and H_2_O fractions. The chloroform fraction was further isolated by a series of column (CC) and thin layer (TLC) chromatography to result in three derivatives of γ-ionylideneacetic acid, phellinulins A–C (**1**–**3**) [19] and eleven new ionone derivatives (**4**–**14**) (Figure 2) along with other chemical constituents. The chemical structures of ionone derivatives **4**–**14** were elucidated as shown below according to their spectroscopic and spectrometric analytical data.

**Figure 3:**
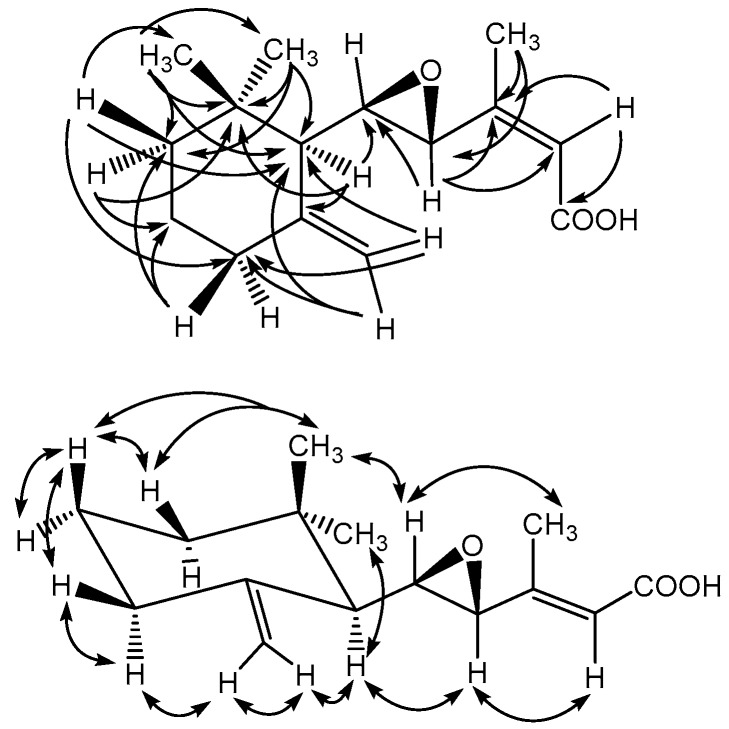
HMBC and NOESY correlations of compound **4**.

Compound **4** was afforded as optically active colorless powder (mp 92–94 °C, ${\left[\alpha \right]}_{D}^{25}$ −52). The High resolution electrospray ionization mass spectrometry (HR-ESI-MS) of **4** showed a pseudomolecular ion peak at *m*/*z* 273.1466 ([M + Na]^+^) corresponding to the molecular formula C_15_H_22_O_3_. The ultraviolet (UV) spectrum of **4** exhibited absorption maxima at 221 nm coincided well with an α,β-unsaturated carbonyl chromophore. The infrared (IR) absorption band at 1689 cm^−1^ indicated the presence of conjugated carbonyl group. The ^1^H-NMR spectrum of **4** displayed three methyl singlets at δ 0.99 (3H, CH_3_-14), 1.04 (3H, CH_3_-15) and 2.12 (3H, CH_3_-12); three mutually coupled methylene groups at δ 1.35 (2H, m, CH_2_-2), 1.52 (2H, m, CH_2_-2) and 2.07 (2H, m, CH_2_-2); three olefinic protons at δ 4.53 (1H, br s, H-13a), 4.81 (1H, br s, H-13b) and 5.95 (1H, s, H-10), respectively. In addition, three methine protons at δ 1.59 (1H, d, *J* = 8.0 Hz, H-6), 2.92 (1H, dd, *J* = 8.0, 1.9 Hz, H-7) and 3.04 (1H, br s, H-8), along with the ^13^C-NMR signals of two sets of carbon-carbon double bond at δ 109.0 (C-13), 115.1 (C-10), 147.7 (C-5) and 157.4 (C-9); two oxymethine carbons of one oxirane ring at δ 59.1 (C-8) and 60.3 (C-7); and one carboxylic acidic carbonyl carbon at δ 171.6 (C-11), evidenced the basic skeleton of **4** as γ-ionylideneacetic acid [28] with oxirane functionality. The ^2^*J*, ^3^*J*-heteronuclear multiple bond correlation (HMBC) correlations (Figure 3) from CH_3_-14 to C-1, -2, -6; from CH_3_-12 to C-8, -9; from H-6 to C-1, -5, -7; from H-8 to C-7, -9, -10; from H-10 to C-9, -11; and from CH_2_-13 to C-4, -6, respectively, further determined the locations of oxirane and terminal methylene groups to be at C-7/C-8 and C-5. The stereochemical configurations of C-1, C-6, C-7 and C-8 were established as shown according to the nuclear Overhauser effect spectroscopy (NOESY) analysis (Figure 3). Conclusively, the chemical structure of **4** was established as shown in Figure 2 and the trivial name phellinulin D was given according to the previous convention [19].

**Figure 4:**
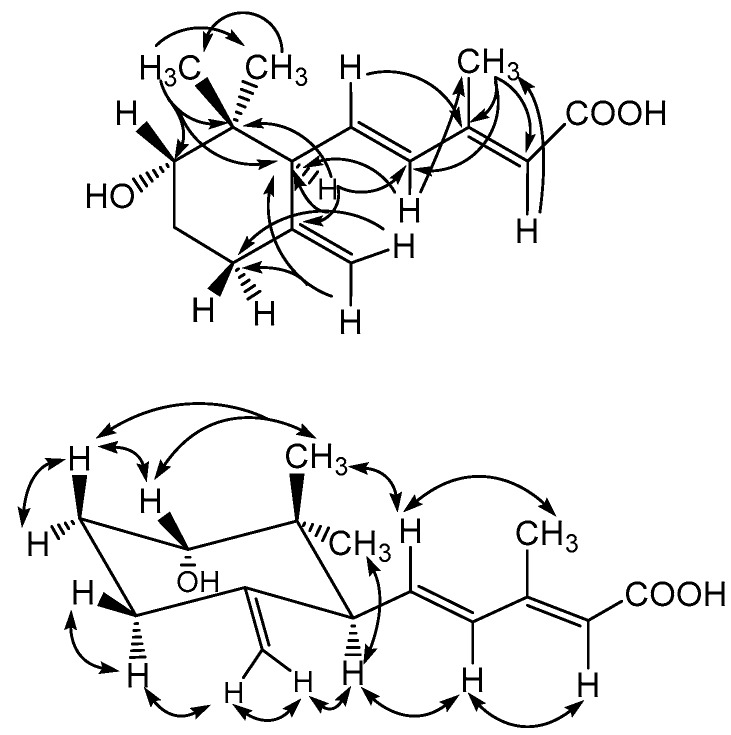
HMBC and NOESY correlations of compound **5**.

Phellinulin E (**5**) was obtained as optically active colorless syrup and its molecular formula was also assigned as C_15_H_22_O_3_ according to the HR-ESI-MS data (*m*/*z* 273.1465, [M + Na]^+^). The UV maxima at 264 nm and IR absorption bands at 3399 and 1686 cm^−1^ suggested the presence of hydroxyl and α,β-conjugated carbonyl functionalities in **5**. The ^1^H and ^13^C-NMR spectral characteristics of **5** (Table 1 and Table 2) also constructed the γ-ionylideneacetic acid basic skeleton as shown above [28]. The ^2^*J*, ^3^*J*-HMBC correlations (Figure 4) from CH_3_-14 to C-1, -2, -6; from CH_3_-12 to C-8, -9, -10; from H-6 to C-1, -5, -8; from H-8 to C-6, -12; from H-10 to C-11, -12; and from CH_2_-13 to C-4, -6, respectively, further determined the locations of hydroxyl and terminal methylene groups to be at C-2 and C-5. The small coupling constant of H-2 (m) and NOE correlations between CH_3_-14 and H-2 indicated the equatorial orientation of H-2. The stereochemical configurations of H-2 and H-6 were further established as β and α according to the NOESY analysis (Figure 4) and therefore **5** was constructed as shown.

Compounds **6**–**8** exhibited UV and IR spectroscopic characteristics similar to those of **5** and were recognized as being isomers of **5**, as the same molecular formula were assigned by HRESIMS analytical data. The HMBC correlation spectroscopic analysis revealed that compounds **6** and **7** were the 3-hydroxyl derivatives and compound **8** was a 4-hydroxyl analog (see Figures S11, S15, and S19). The stereochemical configurations of compounds **6**–**8** were determined by coupling constants of oxymethines and their NOESY spectroscopy (see Figures S12, S16, and S20). Consequently, the chemical structures of **6**–**8** were determined as shown in Figure 2 and named trivially as phellinulins F (**6**), G (**7**) and H (**8**), respectively. 

The HR-ESI-MS of **9** showed a sodium adduct ion peak at *m*/*z* 291.1574 ([M + Na]^+^) corresponding to the formula C_15_H_24_O_4_. Comparison of the UV, IR, ^1^H and ^13^C-NMR data of **9** with phellinulin E (**5**) inferred that the terminal methylene group at C-5 in **5** was oxidized to a dihydroxylation product in **9** (δ_H_ 3.21 (1H, d, *J* = 10.0 Hz, H-13a), 3.29 (1H, d, *J* = 10.0 Hz, H-13b); δ_C_ 70.1 (C-13), 73.8 (C-5)). It was further evidenced by the ^2^*J*, ^3^*J*-HMBC crosspeaks from CH_2_-13 to C-4, -5 and -6. In the NOESY spectral analysis (see Figure S24), the stereochemical configurations of C-5 and C-6 were confirmed and all other proton and carbon signals assignments (Table 1 and Table 2) were established by 2D NMR techniques. Therefore, **9** was named trivially as phellinulin I.

Phellinulins J–L (**10**–**12**) exhibited similar UV, IR and ^1^H-NMR spectroscopic characteristics as compared to those of **5**, except the disappearance of one set of carbon-carbon double bond. The HR-ESI-MS analysis of **10** exhibited a sodiated ion peak at *m*/*z* 259.1676 established the molecular formula of **10** as C_15_H_24_O_2_. The ^1^H-NMR spectrum of **10** displayed two aliphatic fragment signals, in which the connections were proved by its COSY spectrum, including three mutually coupled methylene groups at δ 1.23 (1H, m, H-2a), 1.47 (1H, m, H-2b), 1.54 (2H, m, CH2-3), 2.03 (2H, m, CH2-4); and five mutually coupled protons at δ 1.54 (1H, m, H-7a), 1.64 (1H, m, H-7b), 1.70 (1H, dd, *J* = 12.0, 3.2 Hz, H-6), 1.94(1H, m, H-8a), 2.13 (1H, m, H-8b), respectively. The stereochemical configuration of C-6 was established by NOESY spectral study (see Figure S28) and all other proton and carbon signals assignments (Table 1 and Table 2) were accomplished by 2D NMR techniques. Compounds **11** and **12** were determined as isomers with the same molecular formula C_15_H_24_O_3_ assigned by HRESIMS analytical data. This molecular formula was one oxygen atom more than that of **10** and comparison of their ^1^H-NMR spectra with that of **10** inferred that compounds **11** and **12** were the hydroxyl derivatives of **10**. In **11**, the ^2^*J*, ^3^*J*-HMBC correlations from oxygenated methine (δ 4.04 (1H, d, *J* = 10.0 Hz, H-8)) to C-7, -9, -10 and -12 confirmed the location of hydroxyl group at C-8. Comparatively, **12** is a 3-hydroxylated analog of **10** according to the HMBC analysis in which exhibited correlations from H-4 (δ 1.95) to C-3 (δ 68.2) and H-2 (δ 1.42) to C-3. Furthermore, the stereochemical configurations of **11** and **12** were determined by NOESY analysis (see Figures S32 and S36).

Phellinulin M (**13**) was purified as optically active colorless syrup (${\left[\alpha \right]}_{D}^{25}$ −95) and its HR-ESI-MS analytical data afforded a pseudomolecular ion peak at *m*/*z* 261.1828 indicating the molecular formula of C_15_H_26_O_2_. Comparison of its UV, IR, ^1^H and ^13^C-NMR data with **9** displayed the saturation of one more set of carbon-carbon double bond. There were only three sp^2^ carbons in **13**, including one terminal methylene group attached at C-5 and one carboxylic acid function at C-11. However, the stereochemistry of CH_3_-12 remained unknown due to the lack of further crystallographic evidence. Phellinulin N (**14**) was assigned the molecular formula C_15_H_26_O_3_ according to its HRESIMS data, in which was one more oxygen atom than that of **13**. Comparison of its ^1^H-NMR spectra with that of **13** suggested that compound **14** was the 2-hydroxyl derivative of **13**, confirmed by the HMBC spectroscopic analysis that exhibited ^2^*J*, ^3^*J*-correlations from H-2 (δ 3.67 (1H, dd, *J* = 12.0, 5.0 Hz)) to C-1, -4; and from CH_3_-14 to C-1, -2, -6, respectively. Although the configuration of H-2 was established as β by its coupling constant and NOESY spectrum (see Supporting Information), the stereochemistry of CH_3_-12 remained unknown similar with that of **13**.

In addition to phellinulins A–N (**1**–**14**), nineteen known compounds, including phellilins A–C (**15**–**17**) [29], γ-ionylideneacetic acid (**18**) [28], ergosta-(7,9(11),22t)-trien-3β-ol [30], (−)-cinnamolide [31], ergosterol peroxide [32], 6β-hydroxycinnamolide [33], cinnamolide-3β-ol [34], 5-hydroxy-4-phenyl-5*H*-furan-2-one [35], *p*-hydroxybenzaldehyde [36], lumichrome [37], phenylalanine-leucine [38], phenylalanine-isoleucine [39], phenylalanine [40], proline-valine [41], phenylalanine-proline [42], proline-leucine [43] and phenylalanine-alalinine [44] were also identified by comparison of their physical and spectroscopic data with those reported.

## 3. Discussion

Chronic liver injury resulted from viral, autoimmune, drug-induced, cholestatic and metabolic diseases would induce hepatic fibrosis, a wound-healing response in which several cytokines and compounds would stimulate HSCs and then undergo phenotypic transformation from quiescent cells into proliferative and fibrogenic cells [45,46,47,48]. Liver cirrhosis and further organ failure may occur if the control of fibrosis is poor [11]. Activated HSCs could mediate the release of pro-inflammatory cytokines and tissue inhibitor of metallo-proteinases (TIMP), which would cause the deposition of collagen and further fibrosis [12]. Due to these reasons, to induce the apoptosis of activated HSCs has been claimed to be a potential method of anti-fibrosis [19,49]. More and more scientific reports have evidenced the efficacy of traditional Chinese herbs for treating chronic liver diseases since these herbs usually displayed multi-ingredients, multi-mechanism and low adverse effects [19,49,50]. Various experimental data demonstrated that dimethylnitrosamine (DMN) and transforming growth factor-1 (TGF-1) can induce activated quiescent hepatic stellate cells into proliferating myofibroblast-like cells resulting in hepatic accumulation of extracellular matrix and liver fibrosis [51,52]. The mycelium ethanol extract of *P. linteus* (PLE) exhibited protective effect against hepatic fibrosis in experimental animals in the preliminary study. To validate the inhibition of PLE on hepatic fibrosis in vivo, the histological changes in rat liver tissues were investigated and the experimental results evidenced the protective effect (Figure 1). Both the positive control exposed to silymarin and PLE treatment could effectively attenuate the liver cell damage, fibrosis and collage accumulation induced by DMN application. Therefore, the purified compounds **1**, **4**–**9**, **11**, **13**, **14**, **17** and **18** were subjected to the examination for their inhibition on activated rat HSCs (Table 3) [49]. At the tested concentration (40 μM), **1**, **8**, **9**, **11** and **13** exhibited the protective effects against the activated rat HSCs with the inhibition percentages higher than 50% (Table 3) and western blot analysis of examined compounds were shown in Supporting Information (Figure S45). According to the experimental results, the terminal α,β-unsaturated carboxylic acid moiety was important for the inhibitory bioactivity. Moreover, any substitutions in the C-2 and C-3 will reduce the inhibitory potential significantly. Conclusively, the mycelium of *P. linteus* could be explored as an anti-hepatic fibrosis healthy food in the future and these γ-ionylideneacetic acid and ionone derivatives could be selected as the potential candidates for discovering new lead drugs.

## 4. Materials and Methods 

### 4.1. General

Melting points were determined using Yanagimoto MP-S3 apparatus (Yanagimoto, Kyoto, Japan) without corrections. Optical rotations were measured using a Jasco DIP-370 digital polarimeter (Jasco, Tokyo, Japan). UV spectra were recorded at room temperature on a Hitachi UV-3210 spectrophotometer (Hitachi, Tokyo, Japan), respectively. IR spectra were obtained with a Shimadzu FT-IR DR-8011 spectrophotometer (Shimadzu, Kyoto, Japan). ^1^H and ^13^C-NMR spectra were recorded on Bruker AV-500, Avance-III 400 and Avance 300 NMR spectrometers (Bruker, Billerica, MA, USA). Chemical shifts are shown in δ values (ppm) with tetramethylsilane as an internal standard. The HR-ESI-MS spectra were taken on a Bruker Daltonics APEX II 30e spectrometer (positive-ion mode) (Bruker, Billerica, MA, USA). Column chromatography (CC) was performed on silica gel (70–230 mesh, 230–400 mesh) (Merck, Darmstadt, Germany) and pTLC (preparative thin layer chromatography) was executed on precoated Si gel 60 F254 plates (Merck, Darmstadt, Germany), using UV light to visualize the spots. 

### 4.2. Materials

The dried powder of fermentation cultivated mycelium of *P. linteus* was provided and identified from GeneFerm Biotechnology Co. Ltd. in Taiwan in August 2003. A voucher specimen (Wu 2003010001) has been deposited in the Herbarium of National Cheng Kung University, Tainan, Taiwan.

### 4.3. Animal Experiments

6-Week-old male Wistar rats were purchased from a commercial animal breeder (BioLASCO, Taipei, Taiwan). The rats were acclimated for 1 week and housed in an environmentally controlled room at 22 ± 2 °C, 55 ± 10% relative humidity and in 12-hr light/dark cycles. The rats were randomly grouped into four groups of three rats each (control: saline-treated, DMN-treated, DMN/silymarin-treated, DMN/PLE-treated). Saline and DMN was intraperitoneally injected to rats at 10 mg/kg body weight for 3 consecutive days each week for 4 weeks and the rats of the treatment groups were fed with silymarin (100 mg/kg) and PLE (20 mg/kg) for four weeks via oral route from the first week of DMN exposure. The Committee on Research Involving Animal Subjects of Chang Gung University, Taiwan, approved all experiments in compliance with the standards of Chang Gung University's Committee for the Use and Care of Animals.

### 4.4. Histological Examination

The liver tissue fixed with 10% neutral-buffered formalin was embedded in paraffin and sliced into 5-μm sections that were stained with hematoxylin-eosin and Masson’s trichrome for a histological assessment. The histological images were observed with optical microscopy (Olympus BX51, Tokyo, Japan) in nonconsecutive, randomly chosen ×200 or ×100 histological fields. The digital photomicrographs were then processed with DP-72 (Olympus).

### 4.5. Extraction and Isolation

The dried mycelium powder of *P. linteus* (PL, 1.0 kg) was refluxed with ethanol (4 L × 5 × 4 h). The extract was then filtered with Whatman No. 1 filter paper and the ethanol extracts were combined and concentrated at 40 °C under reduced pressure to obtain the ethanol extract (PLE, 500 g). PLE was further dissolved in water and partition with chloroform to give chloroform (PLEC, 220 g) and water extracts (280 g). The PLEC extract was subjected to silica gel column chromatography eluted with *n*-hexane: ethyl acetate (4:1) to afford six fractions (Frs. 1–6).

Fraction F3 was isolated by silica gel CC with gradient mixture of chloroform and methanol (29:1 to 1:1) to afford three sub fractions (F3-1–F3-3). F3-1 and F3-2 were further purified by silica gel CC and preparative TLC to yield phellinulins A (**1**) (7.1 mg), J (**10**) (11.0 mg), M (**13**) (1.1 mg) and γ-ionylideneacetic acid (**18**) (1.1 g), respectively.

Fraction F4 was performed silica gel CC with gradient mixture of chloroform and methanol (20:1 to 1:1) to afford five sub fractions (F4-1–F4-5). F4-1 was purified by silica gel CC and preparative TLC to yield ergosta-(7,9(11),22t)-trien-3β-ol (106.0 mg) and cinnamolide (8.0 mg). F4-3 was further purified by preparative TLC with chloroform and methanol (29:1) to yield phellinulin B (**2**) (8.0 mg).

Fraction F5 was subjected to silica gel CC eluted with gradient mixture of chloroform and methanol (19:1 to 1:1) to produce five sub fractions (F5-1–F5-5). F5-1 was further purified by preparative TLC with chloroform and methanol (29:1) to yield obtain phellilins A (**15**) (7.0 mg), B (**16**) (1.0 mg) and C (**17**) (10.0 mg), respectively. F5-2 was purified by silica gel CC and preparative TLC to yield phellinulins C (**3**) (1.0 mg), D (**4**) (3.0 mg), E (**5**) (12.0 mg), F (**6**) (32.1 mg), G (**7**) (12.6 mg), H (**8**) (9.1 mg) and I (**9**) (6.0 mg), respectively. F5-3 was further isolated by silica gel CC eluted with chloroform and methanol (15:1) and subsequent preparative TLC purification of the resulted minor fractions to afford phellinulins K (**11**) (62.1 mg), L (**12**) (1.0 mg) and N (**14**) (11.0 mg). F5-4 was further purified by silica gel CC and preparative TLC to yield ergosterol peroxide (5.5 mg), 6β-hydroxycinnamolide (1.0 mg), 3β-hydroxycinnamolide (21.0 mg), 5-hydroxy-4- phenyl-5H-furan-2-one (3.0 mg) and *p*-hydroxybenzaldehyde (2.0 mg), respectively. Recrystallization of the sub fraction F5-5 produced 7,8-dimethylisoalloxazine (3.0 mg).

Fraction F6 was subjected to silica gel CC eluted with step gradient mixture of chloroform and methanol (9:1, 5:1, 3:1, 1:1) to produce three sub fractions (F6-1–F6-3). Subsequent CC on silica gel of the sub fractions 6-1 and 6-2 eluted by the chloroform and methanol mixture resulted in several dipeptide derivatives, including phenylalanine-leucine (1.0 mg), phenylalanine-isoleucine (3.0 mg), phenylalanine (1.9 mg), proline-valine (26.0 mg), phenylalanine-proline (2.0 mg), proline-leucine (4.0 mg) and phenylalanine-alalinine (11.0 mg), respectively.

*Phellinulin D* (**4**). Colorless powder (CHCl_3_); mp 92–94 °C; ${\left[\alpha \right]}_{D}^{25}$ −52 (*c* 0.3, MeOH); UV (MeOH) λ_max_ (log ε) 221.2 (2.99) nm; IR (neat) ν_max_ 3074, 2931, 2870, 1689, 1647, 1438, 1238 cm^−1^; ^1^H-NMR (300 MHz, CDCl_3_) and ^13^C-NMR (75 MHz, CDCl_3_), see Table 1 and Table 2; HRESIMS *m*/*z* 273.1466 ([M + Na]^+^, calcd for C_15_H_22_O_3_Na, 273.1467).

*Phellinulin E* (**5**). Colorless syrup (CHCl_3_); ${\left[\alpha \right]}_{D}^{25}$ −53 (*c* 0.3, MeOH); UV (MeOH) λ_max_ (log ε) 263.8 (4.02) nm; IR (neat) ν_max_ 3399, 3074, 2943, 1686, 1608, 1254 cm^−1^; ^1^H-NMR (400 MHz, CDCl_3_) and ^13^C-NMR (75 MHz, CDCl_3_), see Table 1 and Table 2; HRESIMS *m*/*z* 273.1465 ([M + Na]^+^, calcd for C_15_H_22_O_3_Na, 273.1467).

*Phellinulin F* (**6**). White needles (CHCl_3_); mp 138–140 °C; ${\left[\alpha \right]}_{D}^{25}$ −80 (*c* 0.3, MeOH); UV (MeOH) λ_max_ (log ε) 263.0 (4.29) nm; IR (neat) ν_max_ 2954, 2870, 1685, 1613, 1250, 1176 cm^−1^; ^1^H-NMR (500 MHz, Acetone-*d*_6_) and ^13^C-NMR (75 MHz, CDCl_3_), see Table 1 and Table 2; HRESIMS *m/z* 273.1465 ([M + Na]^+^, calcd for C_15_H_22_O_3_Na, 273.1467).

*Phellinulin G* (**7**). Colorless syrup (CHCl_3_); ${\left[\alpha \right]}_{D}^{25}$ −46 (*c* 0.1, MeOH); UV (MeOH) λ_max_ (log ε) 258.8 (4.28) nm; IR (neat) ν_max_ 3360, 2959, 2928, 1690, 1609, 1250 cm^−1^; ^1^H-NMR (500 MHz, CDCl_3_) and ^13^C-NMR (75 MHz, CDCl_3_), see Table 1 and Table 2; HRESIMS *m*/*z* 273.1464 ([M + Na]^+^, calcd for C_15_H_22_O_3_Na, 273.1467).

*Phellinulin H* (**8**). White powder (CHCl_3_); mp 125–127 °C; ${\left[\alpha \right]}_{D}^{25}$ −135 (*c* 0.1, MeOH); UV (MeOH) λ_max_ (log ε) 260.0 (3.89) nm; IR (neat) ν_max_ 3360, 2947, 2866, 1686, 1609, 1254 cm^−1^; ^1^H-NMR (500 MHz, CDCl_3_) and ^13^C-NMR (75 MHz, CDCl_3_), see Table 1 and Table 2; HRESIMS *m*/*z* 273.1465 ([M + Na]^+^, calcd for C_15_H_22_O_3_Na, 273.1467).

*Phellinulin I* (**9**). Colorless needles (CHCl_3_); mp 180–183 °C; ${\left[\alpha \right]}_{D}^{25}$ −83 (*c* 0.2, MeOH); UV (MeOH) λ_max_ (log ε) 264.0 (3.50) nm; IR (neat) ν_max_ 3418, 2947, 2928, 2859, 1686, 1451, 1250 cm^−1^; ^1^H-NMR (500 MHz, Acetone-*d*_6_) and ^13^C-NMR (75 MHz, CDCl_3_), see Table 1 and Table 2; HRESIMS *m*/*z* 291.1574 ([M + Na]^+^, calcd for C_15_H_24_O_4_Na, 291.1572).

*Phellinulin J* (**10**). White powder (CHCl_3_); mp 59–60 °C; ${\left[\alpha \right]}_{D}^{25}$ −59 (*c* 0.1, MeOH); UV (MeOH) λ_max_ (log ε) 264.2 (4.57) nm; IR (neat) ν_max_ 2940, 2870, 1690, 1640, 1424, 1253 cm^−1^; ^1^H-NMR (500 MHz, CDCl_3_) and ^13^C-NMR (75 MHz, CDCl_3_), see Table 1 and Table 2; HRESIMS *m*/*z* 259.1676 ([M + Na]^+^, calcd for C_15_H_24_O_2_Na, 259.1674).

*Phellinulin K* (**11**). Colorless syrup (CHCl_3_); ${\left[\alpha \right]}_{D}^{25}$ −84 (*c* 0.8, MeOH); UV (MeOH) λ_max_ (log ε) 219.2 (3.74) nm; IR (neat) ν_max_ 2932, 2866, 1694, 1647, 1246 cm^−1^; ^1^H-NMR (500 MHz, CDCl_3_) and ^13^C-NMR (75 MHz, CDCl_3_), see Table 1 and Table 2; HRESIMS *m*/*z* 275.1622 ([M + Na]^+^, calcd for C_15_H_24_O_3_Na, 275.1623).

*Phellinulin L* (**12**). Colorless syrup (CHCl_3_); ${\left[\alpha \right]}_{D}^{25}$ −111 (*c* 0.2, MeOH); UV (MeOH) λ_max_ (log ε) 219.0 (3.66) nm; IR (neat) ν_max_ 2947, 2874, 1690, 1643, 1427, 1246, 675 cm^−1^; ^1^H-NMR (500 MHz, CDCl_3_) and ^13^C-NMR (75 MHz, CDCl_3_), see Table 1 and Table 2; HRESIMS *m*/*z* 275.1621 ([M + Na]^+^, calcd for C_15_H_24_O_3_Na, 275.1623).

*Phellinulin M* (**13**). Colorless syrup (CHCl_3_); ${\left[\alpha \right]}_{D}^{25}$ −95 (*c* 0.2, MeOH); UV (MeOH) λ_max_ (log ε) 202.4 (3.35); 264.4 nm; IR (neat) ν_max_ 2936, 2870, 1708, 1647, 1447, 1292 cm^−1^; ^1^H-NMR (300 MHz, CDCl_3_) and ^13^C-NMR (75 MHz, CDCl_3_), see Table 1 and Table 2; HRESIMS *m*/*z* 261.1828 ([M + Na]^+^, calcd for C_15_H_26_O_2_Na, 261.1830).

*Phellinulin N* (**14**). Colorless syrup (CHCl_3_); ${\left[\alpha \right]}_{D}^{25}$ −67 (*c* 0.1, MeOH); UV (MeOH) λ_max_ (log ε) 264.6 (3.49), 220.4 (3.69) nm; IR (neat) ν_max_ 3363, 2939, 2870, 1709, 1385, 1256, 1261 cm^−1^; ^1^H-NMR (500 MHz, CDCl_3_) and ^13^C-NMR (75 MHz, CDCl_3_), see Table 1 and Table 2; HRESIMS *m*/*z* 277.1781 ([M + Na]^+^, calcd for C_15_H_26_O_3_Na, 277.1780).

### 4.6. Determination of the Inhibition Effects on Activated Rat HSCs

The immortalized rat myofibroblast cell line HSC-T6 was a kind gift of Dr. Scott L. Friedman (Mount Sinai School of Medicine, New York, NY, USA). The HSC-T6 cells were maintained in Waymouth medium containing 10% fetal bovine serum (FBS) at 37 °C in a humidified atmosphere of 5% CO_2_. A total of 1 × 10^5^ cells were seeded in 24-well plates for 24 h and made quiescent by incubating in medium containing 0.2% FBS overnight. After treating with 40 μM of tested compounds for 72 h, isopropanol solution mixed with tetrazolium salt was added to the wells and incubated for additional 4 h at 37 °C. The optical density of the dissolved material was measured spectrophotometrically at 570 nm and assays were performed at least in triplicate [51].

### 4.7. Statistical Analysis

Experimental results were expressed as mean ± standard deviation (SD) of three parallel measurements (SigmaPlot 8.0; SPSS Inc. Chicago, IL, USA).

## Figures and Tables

**Table 1:** ^1^H-NMR spectroscopic data of compounds **4**–**14** (δ_H_ mult. (*J* in Hz)).

Position	4 *^a^*	5 *^b^*	6 *^c^*	7 *^c^*	8 *^c^*	9 *^c^*	10 *^c^*	11 *^c^*	12 *^c^*	13 *^a^*	14 *^c^*
2	1.35 m	3.62 m	1.55 m	1.35 t (13.0) 1.84 dd (13.0, 5.0)	1.45 m 1.57 m	1.23 dt (14.0, 3.4) 1.57 dt (14.0, 4.1)	1.23 m 1.48 m	1.24 m 1.42 m	1.42 dd (12.0, 12.0) 1.54 dd (12.0, 5.0)	1.18 m	3.67 dd (12.0, 5.0)
3	1.52 m	1.66 m 1.86 m	3.77 m	3.83 m	1.53 m 1.97 m	1.45 m 1.91 m	1.54 m	1. 51 m	3.77 m	1.42 m	1.33 m 1.50 m
4	2.07 m	2.24 m 2.37 m	2.15 m 2.45 dd (11.0, 9.9)	2.00 t (12.0) 2.70 dd (12.0, 5.0)	4.06 t (5.0)	1.45 m 1.73 d (13.0)	2.03 m	2.06 m	1.95 m 2.43 dd (12.0, 5.0)	2.00 m	2.15 m
6	1.59 d (8.0)	2.93 d (9.4)	2.62 d (9.4)	2.52 d (5.0)	2.53 d (9.9)	2.01 d (10.0)	1.70 dd (12.0, 3.2)	2.10 dd (14.0, 3.5)	1.95 m 2.10 m	1.67 dd (10.6, 4.0)	1.87 dd (14.0, 3.3)
7	2.92 dd (8.0, 1.9)	6.30 dd (15.0. 9.4)	6.48 dd (16.0, 9.4)	6.17 br s	6.29 dd (16.0, 9.9)	6.41 dd (16.0, 10.0)	1.54 m 1.64 m	1.52 dt (10.0, 3.5) 1.67 dt (14.0, 3.5)	1.54 m 1.65 m	1.49 m	1.50 m 1.80 dd (14.0, 6.0)
8	3.04 br s	6.17 d (15.0)	6.24 d (16.0)	6.18 d (3.7)	6.13 d (16.0)	6.20 d (16.0)	1.93 m	4.04 d (10.0)	6.24 d (16.0)	1.51 m	1.13 m
9	-	-	-	-	-	-	-	-	-	1.92 m	1.93 m
10	5.95 s	5.75 s	5.76 s	5.77 s	5.76 s	5.76 s	5.69 s	5.98 s	5.69 s	2.14 dd (15.0, 8.0) 2.33 dd (15.0, 6.0)	2.15 m 2.29 m
12	2.12 s	2.31 s	2.29 s	2.33 s	2.34 s	2.31 s	2.16 s	2.10 s	2.16 s	0.96 d (6.6)	4.52 br s 4.78 br s
13	4.53 s 4.81 s	4.58 br s 4.81 br s	4.77 d (1.9)	4.59 d (0.9) 4.91 br s	4.69 br s 5.14 s	3.21 d (10.0) 3.29 d (10.0)	4.55 br s 4.07 br s	4.67 d (2.0) 4.85 br s	4.70 br s 4.89 br s	4.52 d (1.6) 4.73 d (1.6)	4.55 br s 4.07 br s
14	0.99 s	0.90 s	0.89 s	0.84 s	0.84 s	0.83 s	0.84 s	0.83 s	0.91 s	0.83 (3H, s)	0.89 s
15	1.04 s	0.93 s	0.92 s	0.94 s	0.88 s	1.12 s	0.92 s	0.93 s	0.95 s	0.91 (3H, s)	0.96 s

^1^H-NMR data measured in CDCl_3_ at *^a^* 300 MHz, *^b^* 400 MHz and *^c^* 500 MHz.

**Table 2:** ^13^C-NMR spectroscopic data of compounds **4**–**14** (CDCl_3_).

Position	4 *^a^*	5 *^b^*	6 *^c^*	7 *^c^*	8 *^c^*	9 *^c^*	10 *^c^*	11 *^c^*	12 *^c^*	13 *^a^*	14 *^c^*
1	35.6	39.8	34.7	36.0	35.8	34.7	34.9	34.5	34.6	34.8	39.0
2	38.8	74.9	44.0	50.1	38.2	42.3	36.1	35.7	43.5	35.2	73.9
3	23.2	30.3	66.9	67.6	32.3	18.4	23.6	23.5	68.2	36.1	23.3
4	34.9	29.9	41.1	45.6	72.8	35.8	32.3	32.1	40.2	32.3	29.9
5	147.7	148.1	147.6	146.4	151.3	73.8	148.7	149.0	145.7	149.2	147.4
6	56.9	54.3	58.2	56.5	56.1	54.5	53.6	49.8	52.9	53.9	53.8
7	60.3	136.1	136.2	135.8	135.9	136.9	24.3	32.5	24.7	23.5	31.4
8	59.1	135.9	134.6	136.6	136.0	137.1	39.8	74.3	39.6	23.7	35.2
9	157.4	154.6	152.4	154.3	137.1	153.3	164.0	164.1	163.5	30.3	30.2
10	115.1	117.6	118.1	117.6	117.8	118.3	114.9	113.7	114.8	42.1	42.2
11	171.6	171.8	167.2	171.6	171.9	168.2	172.4	172.1	170.9	180.0	178.8
12	14.8	14.2	13.0	14.2	14.3	13.9	19.3	15.9	19.3	19.5	19.5
13	109.0	109.8	110.6	111.3	106.3	70.1	109.5	110.1	113.1	108.9	110.5
14	28.9	22.8	27.4	21.5	21.9	32.8	26.6	26.5	28.6	26.4	21.6
15	23.5	23.6	28.3	30.7	29.9	22.9	28.3	28.2	28.2	28.4	24.2

^13^C-NMR data measured in CDCl_3_ at *^a^* 75 MHz, *^b^* 100 MHz and *^c^* 125 MHz.

**Table 3:** Inhibitory effects of isolated compounds on activated rat HSCs.

Sample	Expression of α-SMA ^a^ (Fold of Base)	Inhibition Percentage (%)
Control	0.53 ± 0.01	(−) ^d^
TGF	1.65 ± 0.03	(−) ^d^
TGF + PLE ^b^	1.19 ± 0.02	27.8
TGF + **1** ^c^	0.54 ± 0.01	67.2
TGF + **4** ^c^	1.58 ± 0.03	4.2
TGF + **5** ^c^	1.26 ± 0.02	23.6
TGF + **6** ^c^	1.21 ± 0.02	26.7
TGF + **7** ^c^	1.40 ± 0.03	15.2
TGF + **8** ^c^	0.65 ± 0.01	60.6
TGF + **9** ^c^	0.81 ± 0.02	50.9
TGF + **10** ^c^	1.35 ± 0.03	18.2
TGF + **11** ^c^	0.53 ± 0.01	67.9
TGF + **13** ^c^	0.72 ± 0.01	56.4
TGF + **14** ^c^	0.87 ± 0.02	47.2
TGF + **17** ^c^	1.25 ± 0.02	24.2
TGF + **18** ^c^	0.83 ± 0.02	49.7
Silymarin ^c^	1.05 ± 0.02	36.4

^a^ The expressions of α-SMA were presented as mean ± SD (*n* = 3). ^b^ Test concentration: 40 μg/mL. ^c^ Test concentration: 40 μM. (−) ^d^: Not determined.

## Supplementary Materials

The following are available online, Figures S1–S44, 2D spectra of compounds **4**–**14**; Figure S45, western blot analysis of examined compounds.
