# Supplementary material for: Hepatoprotective Principles and Other Chemical Constituents from the Mycelium of Phellinus linteus
Source: Molecules. 2018 Jul 12;23(7):1705. doi: 10.3390/molecules23071705 (PMC6099599; doi:10.3390/molecules23071705)
Supplement: Supplementary file 1 [file molecules-23-01705-s001.pdf]

Supporting Information

Hepatoprotective Principles and Other Chemical Constituents from the Mycelium of *Phellinus linteus*

Shiow-Chyn Huang [1,*], Pei-Wen Wang [2], Ping-Chung Kuo [3], Hsin-Yi Hung [3], and Tai-Long Pan [4]

[1] Department of Pharmacy, Chia-Nan University of Pharmacy and Science, Tainan 717, Taiwan

[2] Department of Medical Research, China Medical University Hospital, China Medical University, Taichung 404, Taiwan; pwwang5105@hotmail.com

[3] School of Pharmacy, College of Medicine, National Cheng Kung University, Tainan 701, Taiwan; z10502016@email.ncku.edu.tw (P.-C.K.); z10308005@email.ncku.edu.tw (H.-Y.H.)

[4] School of Traditional Chinese Medicine, Chang Gung University; Research Center for Chinese Herbal Medicine and Research Center for Food and Cosmetic Safety, College of Human Ecology, Chang Gung University of Science and Technology; Liver Research Center, Chang Gung Memorial Hospital, Taoyuan 333, Taiwan; pan@mail.cgu.edu.tw

***** Correspondence: schuang@mail.cnu.edu.tw; Tel.: +886-6-266-4911 ext. 2105

Contents

Fig. S1. ^{1}H NMR spectrum of **4**
Fig. S2. ^{13}C NMR spectrum of **4**
Fig. S3. HMBC spectrum of **4**
Fig. S4. NOESY spectrum of **4**
Fig. S5. ^{1}H NMR spectrum of **5**
Fig. S6. ^{13}C NMR spectrum of **5**
Fig. S7. HMBC spectrum of **5**
Fig. S8. NOESY spectrum of **5**
Fig. S9. ^{1}H NMR spectrum of **6**
Fig. S10. ^{13}C NMR spectrum of **6**
Fig. S11. HMBC spectrum of **6**
Fig. S12. NOESY spectrum of **6**
Fig. S13. ^{1}H NMR spectrum of **7**
Fig. S14. ^{13}C NMR spectrum of **7**
Fig. S15. HMBC spectrum of **7**
Fig. S16. NOESY spectrum of **7**
Fig. S17. ^{1}H NMR spectrum of **8**
Fig. S18. ^{13}C NMR spectrum of **8**
Fig. S19. HMBC spectrum of **8**
Fig. S20. NOESY spectrum of **8**
Fig. S21. ^{1}H NMR spectrum of **9**
Fig. S22. ^{13}C NMR spectrum of **9**
Fig. S23. HMBC spectrum of **9**
Fig. S24. NOESY spectrum of **9**
Fig. S25. ^{1}H NMR spectrum of **10**
Fig. S26. ^{13}C NMR spectrum of **10**
Fig. S27. HMBC spectrum of **10**
Fig. S28. NOESY spectrum of **10**
Fig. S29. ^{1}H NMR spectrum of **11**
Fig. S30. ^{13}C NMR spectrum of **11**
Fig. S31. HMBC spectrum of **11**

Fig. S32. NOESY spectrum of **11**

Fig. S33. ^{1}H NMR spectrum of **12**

Fig. S34. ^{13}C NMR spectrum of **12**

Fig. S35. HMBC spectrum of **12**

Fig. S36. NOESY spectrum of **12**

Fig. S37. ^{1}H NMR spectrum of **13**

Fig. S38. ^{13}C NMR spectrum of **13**

Fig. S39. HMBC spectrum of **13**

Fig. S40. NOESY spectrum of **13**

Fig. S41. ^{1}H NMR spectrum of **14**

Fig. S42. ^{13}C NMR spectrum of **14**

Fig. S43. HMBC spectrum of **14**

Fig. S44. NOESY spectrum of **14**

Fig. S45. Western blot analysis of examined compounds

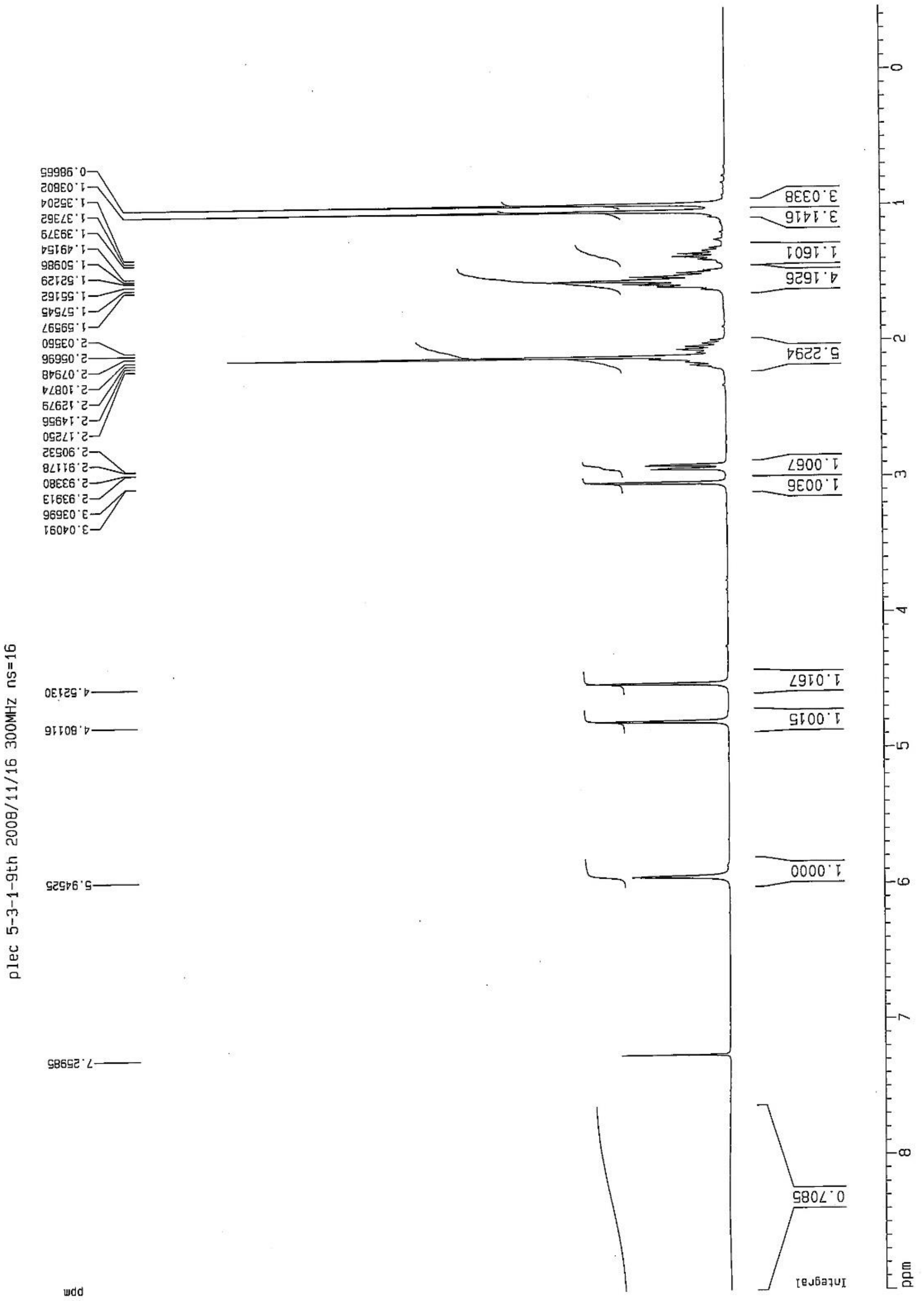

Fig. S1. ^{1}H NMR spectrum of **4**

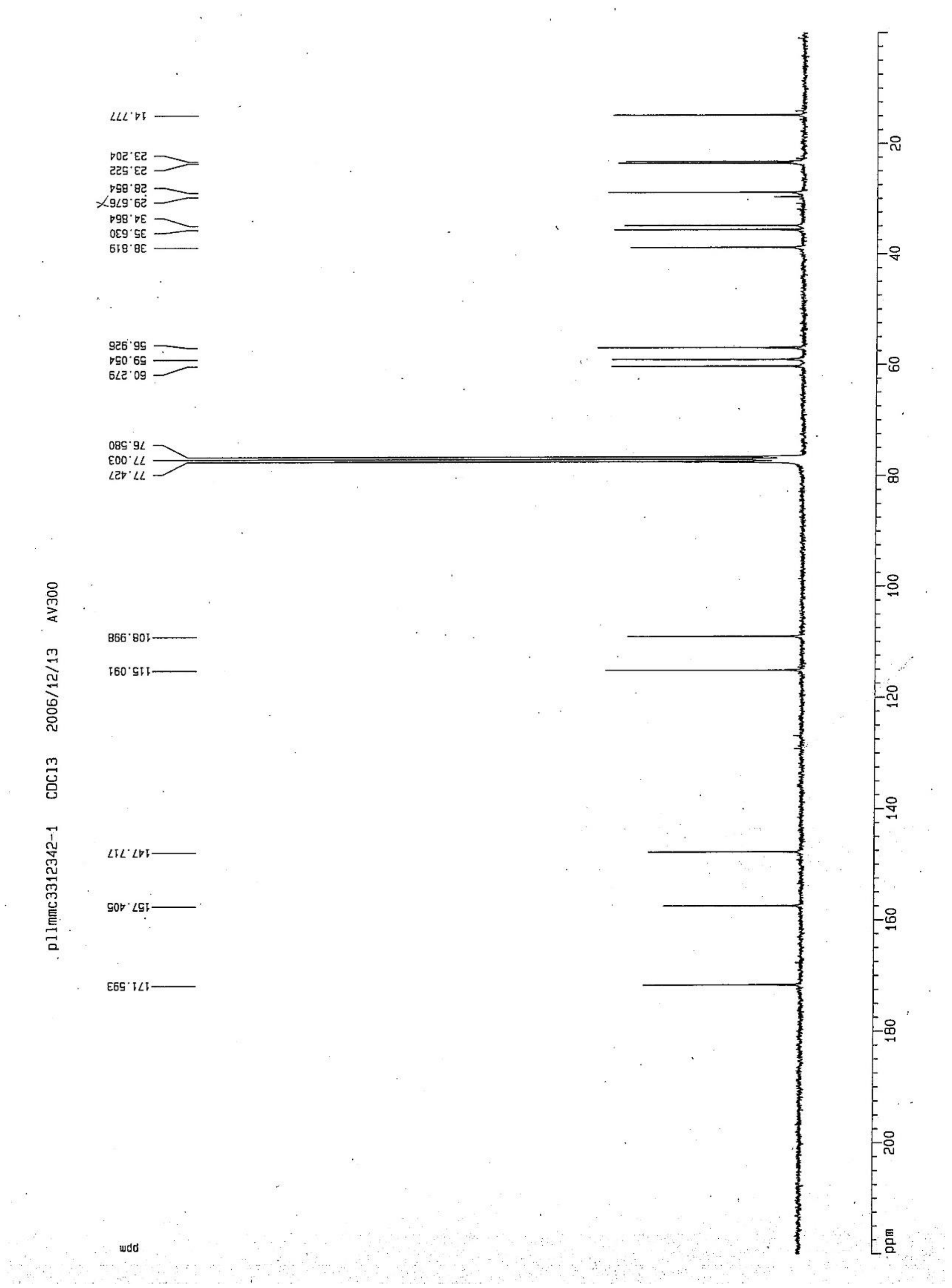

Fig. S2. ^{13}C NMR spectrum of **4**

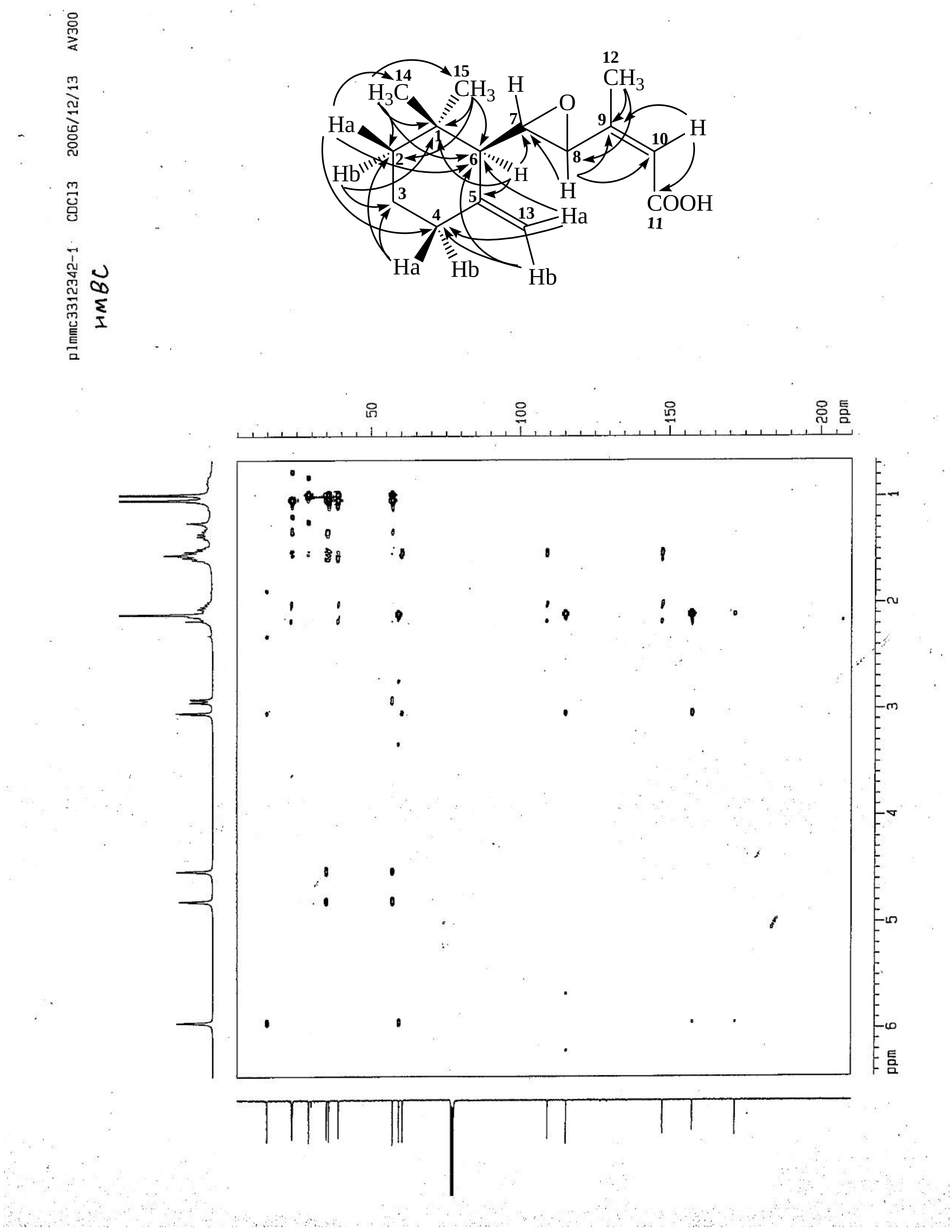

Fig. S3. HMBC spectrum of **4**

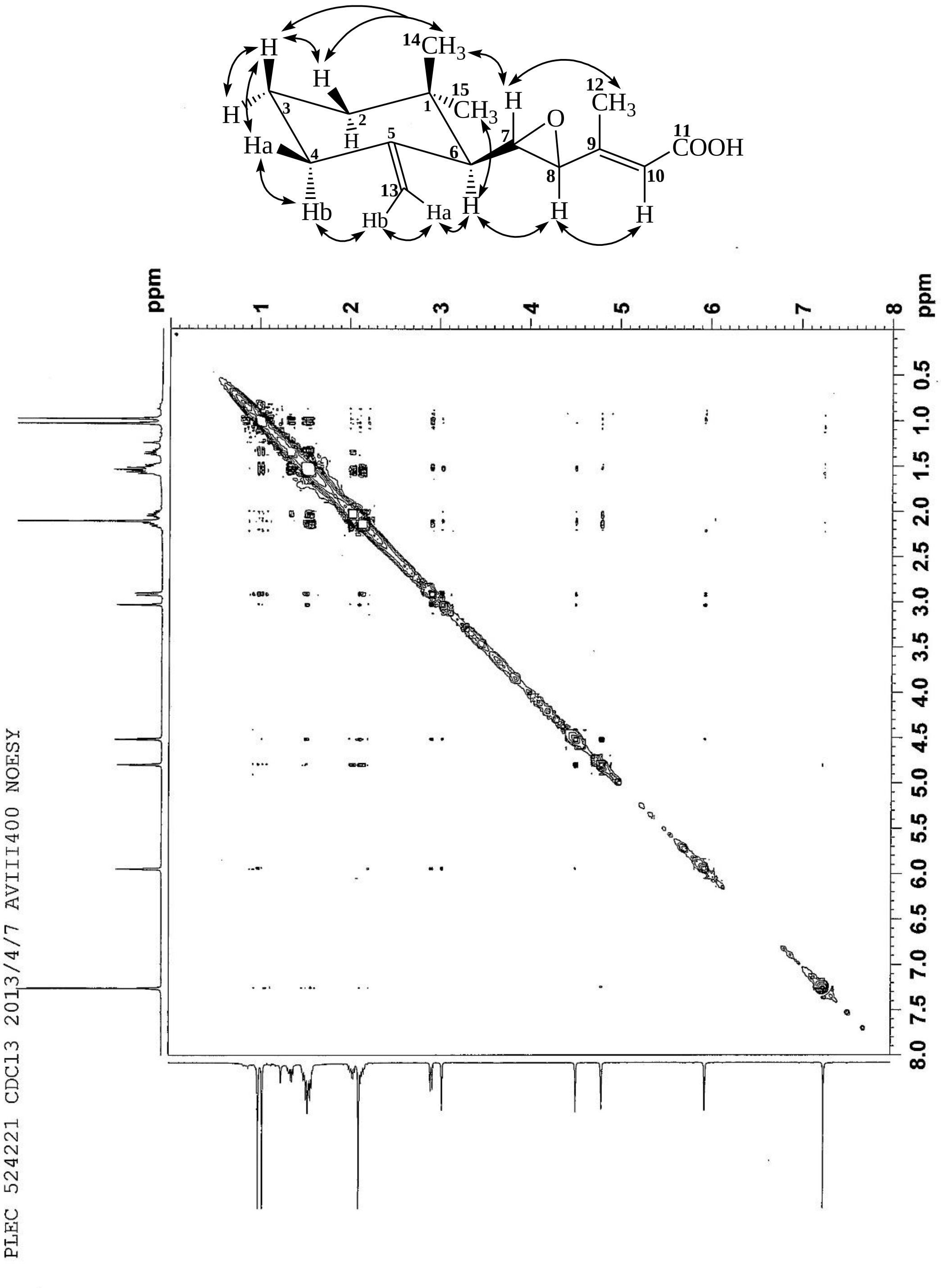

Fig. S4. NOESY spectrum of **4**

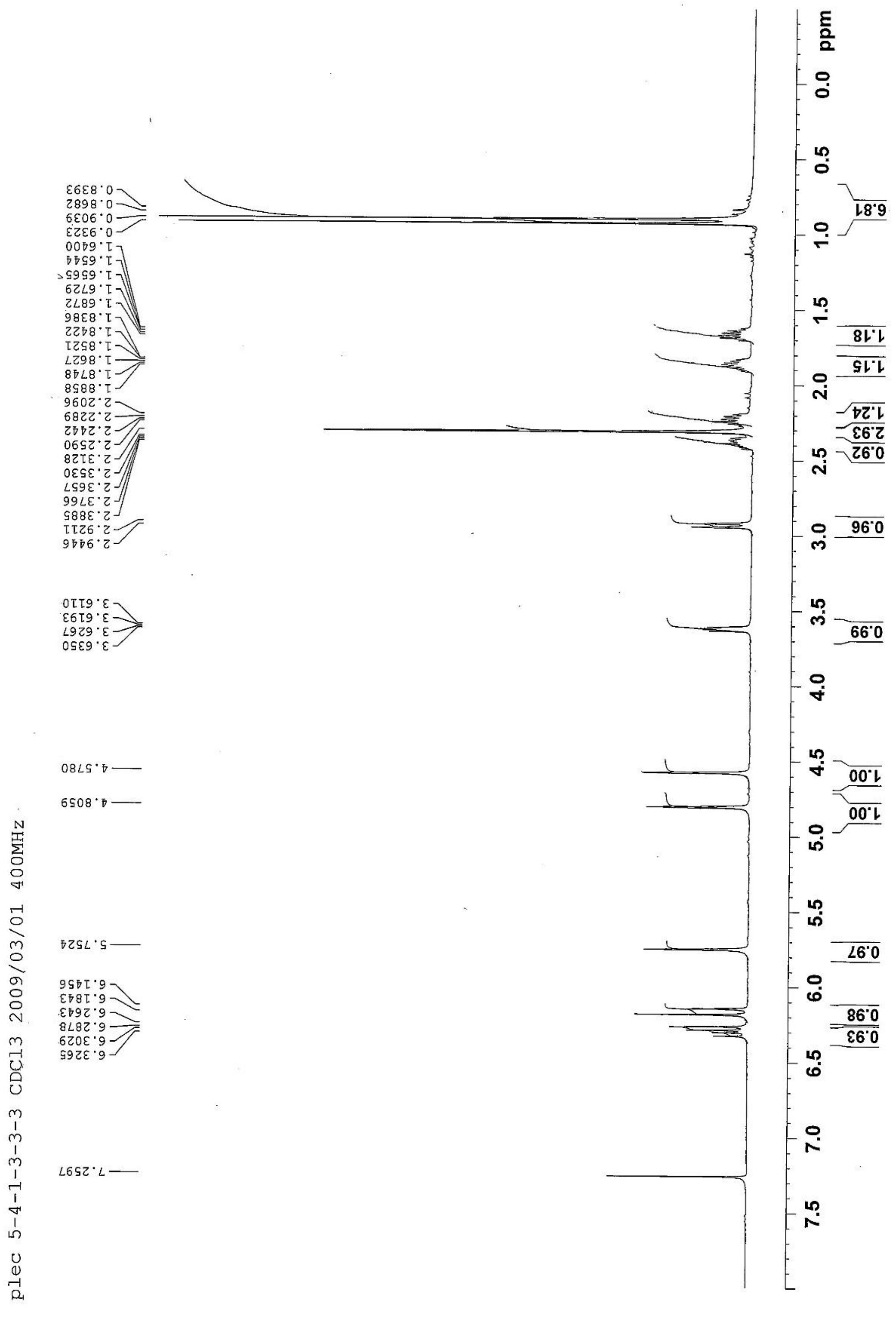

Fig. S5. ^{1}H NMR spectrum of **5**

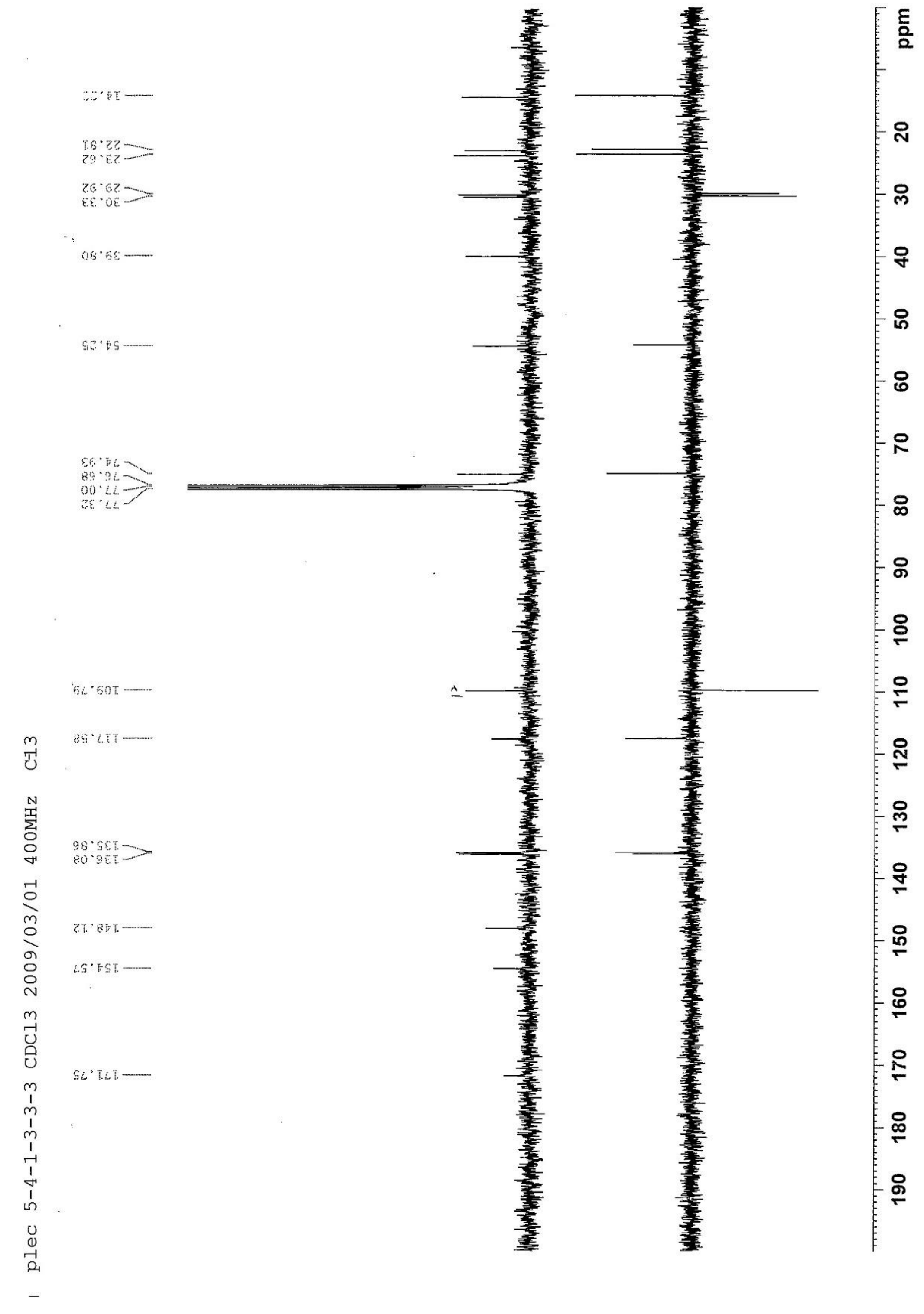

Fig. S6. ^{13}C NMR spectrum of **5**

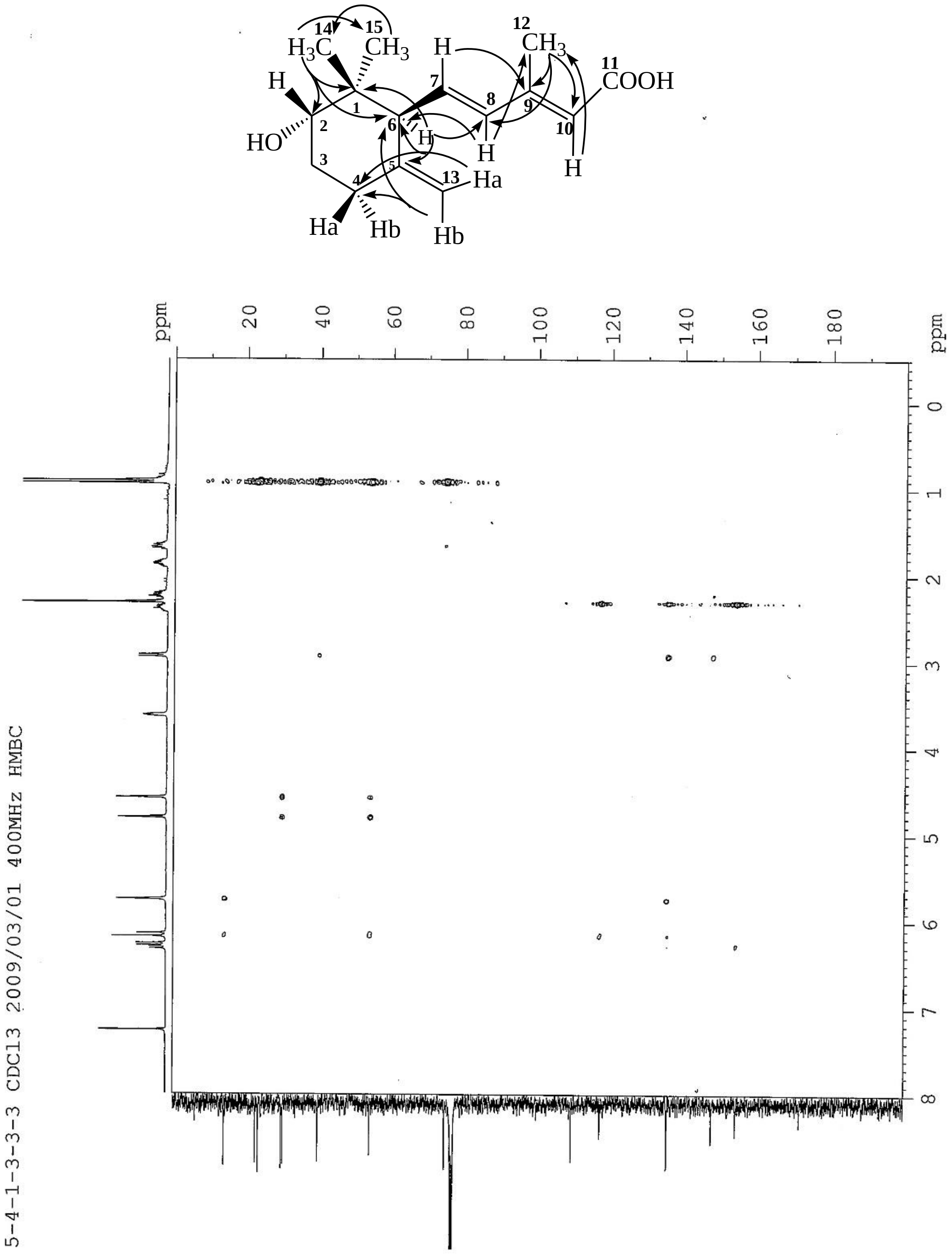

Fig. S7. HMBC spectrum of **5**

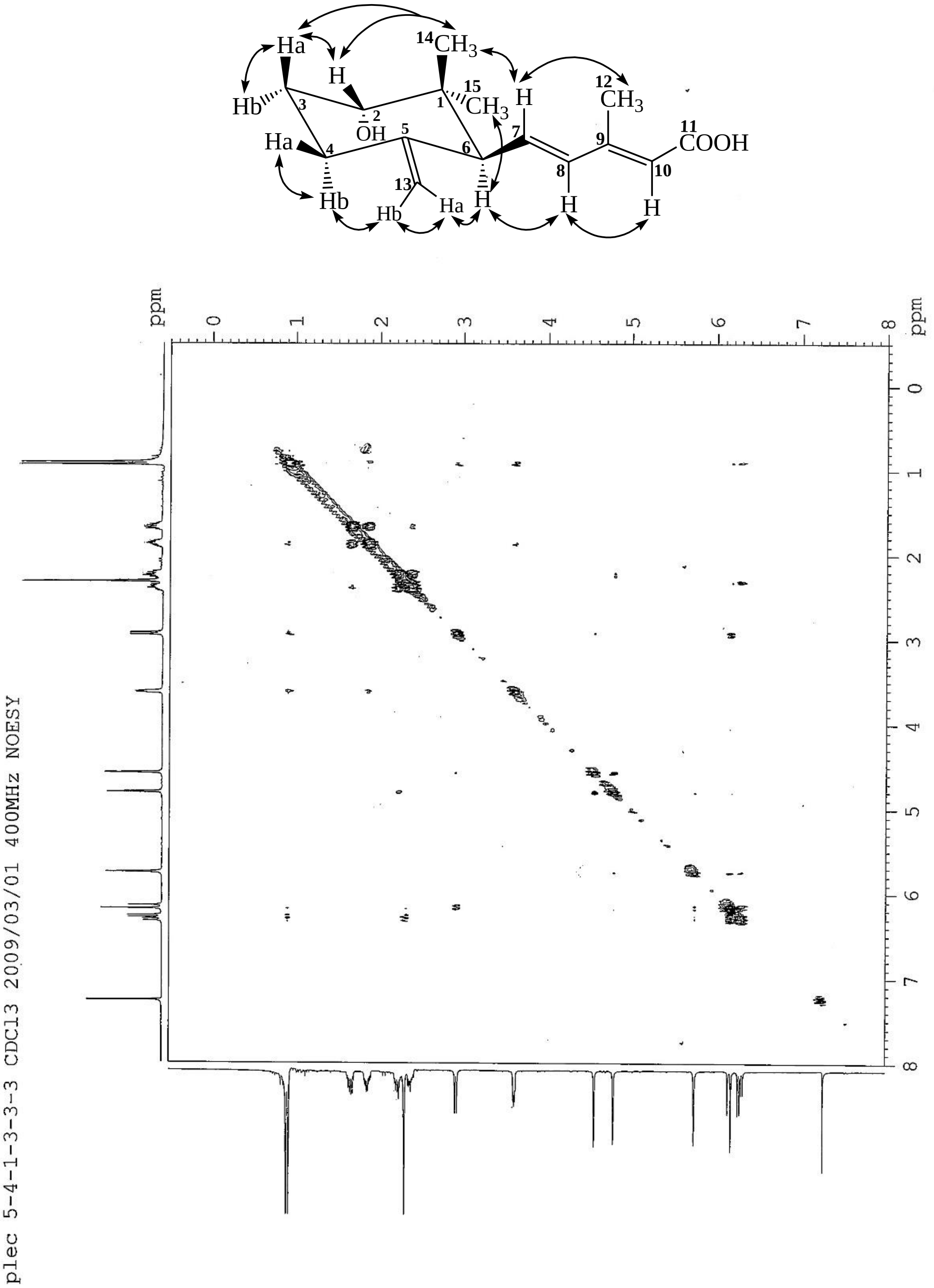

Fig. S8. NOESY spectrum of **5**

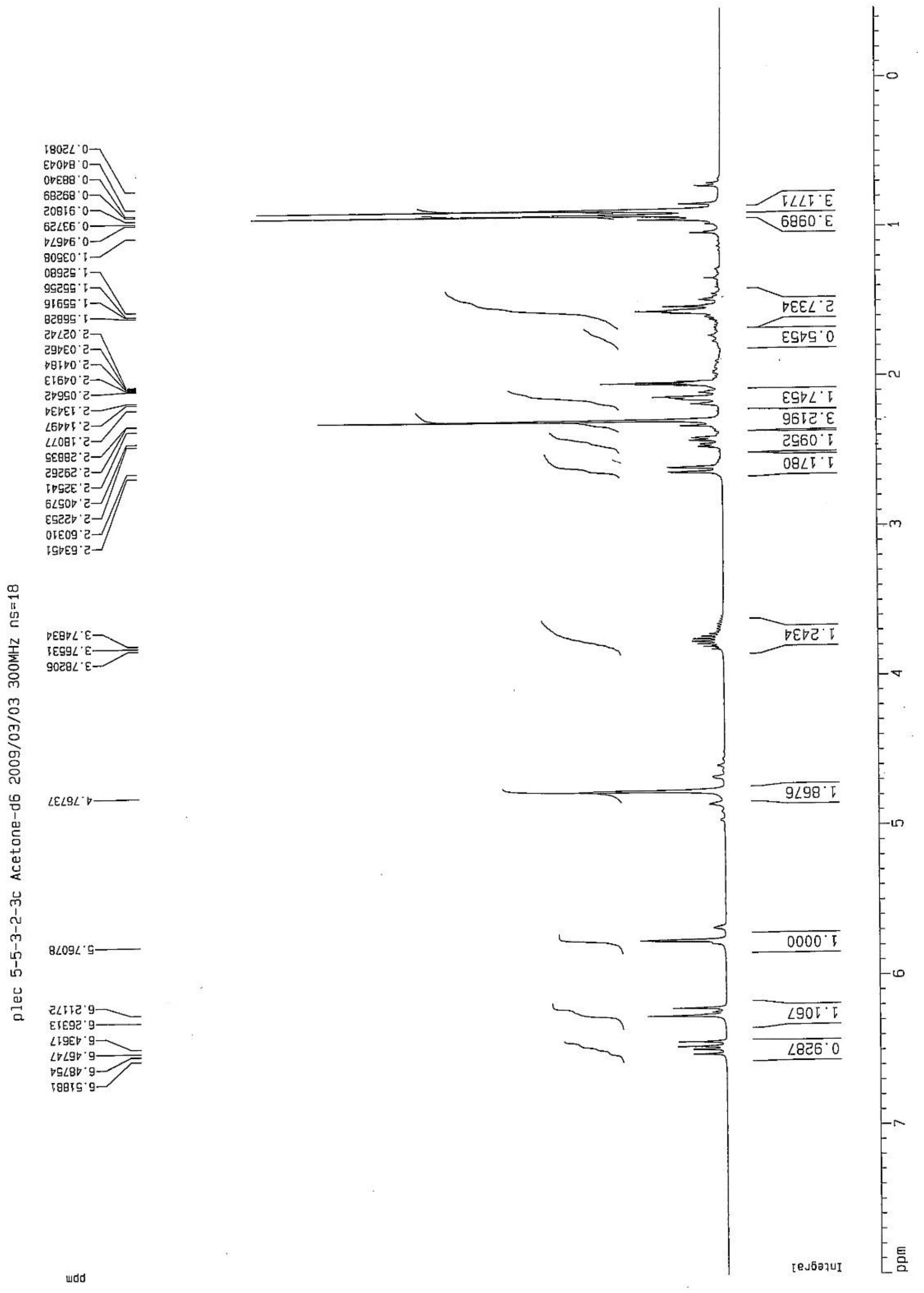

Fig. S9. ^{1}H NMR spectrum of **6**

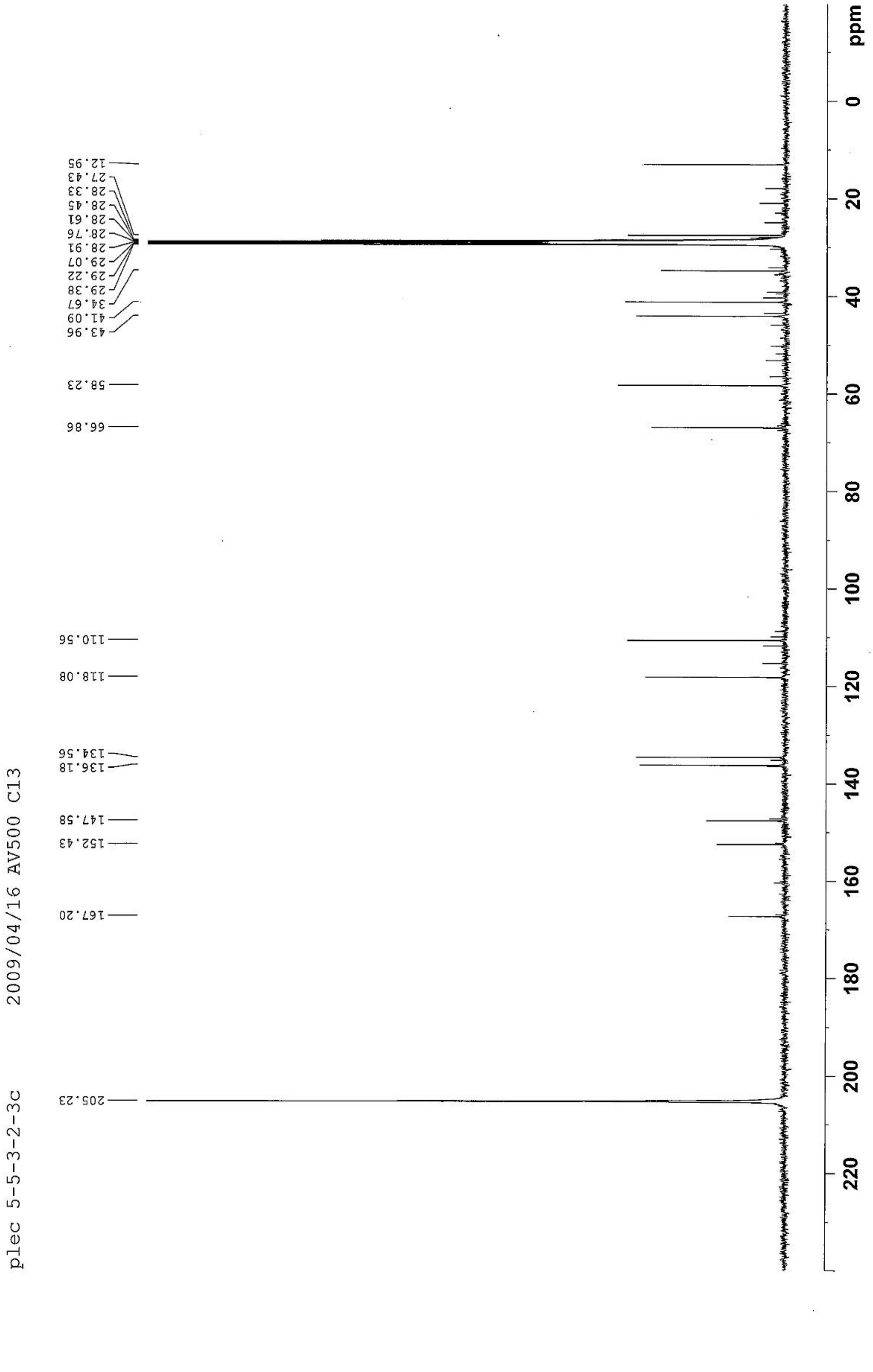

Fig. S10. ^{13}C NMR spectrum of **6**

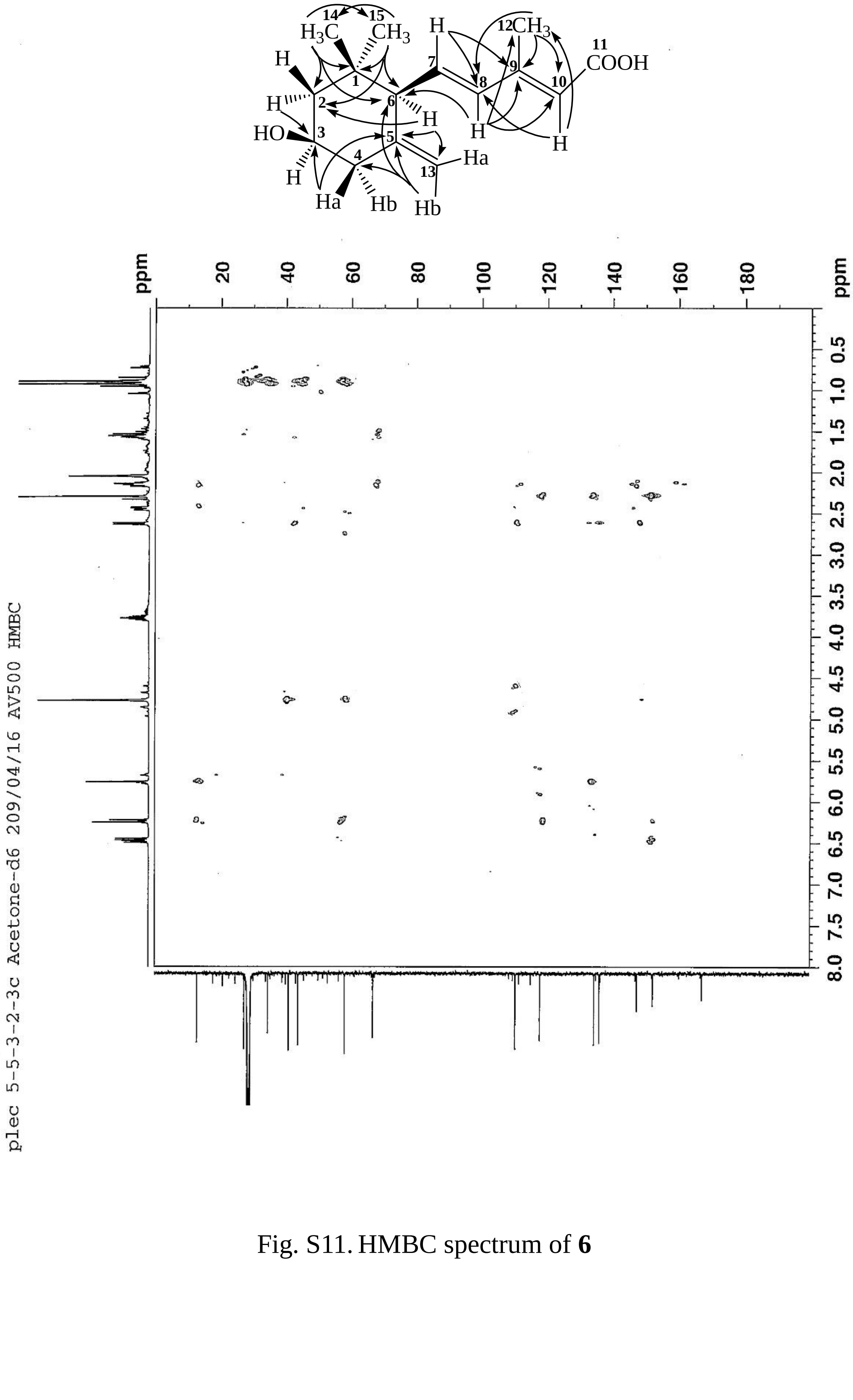

Fig. S11. HMBC spectrum of **6**

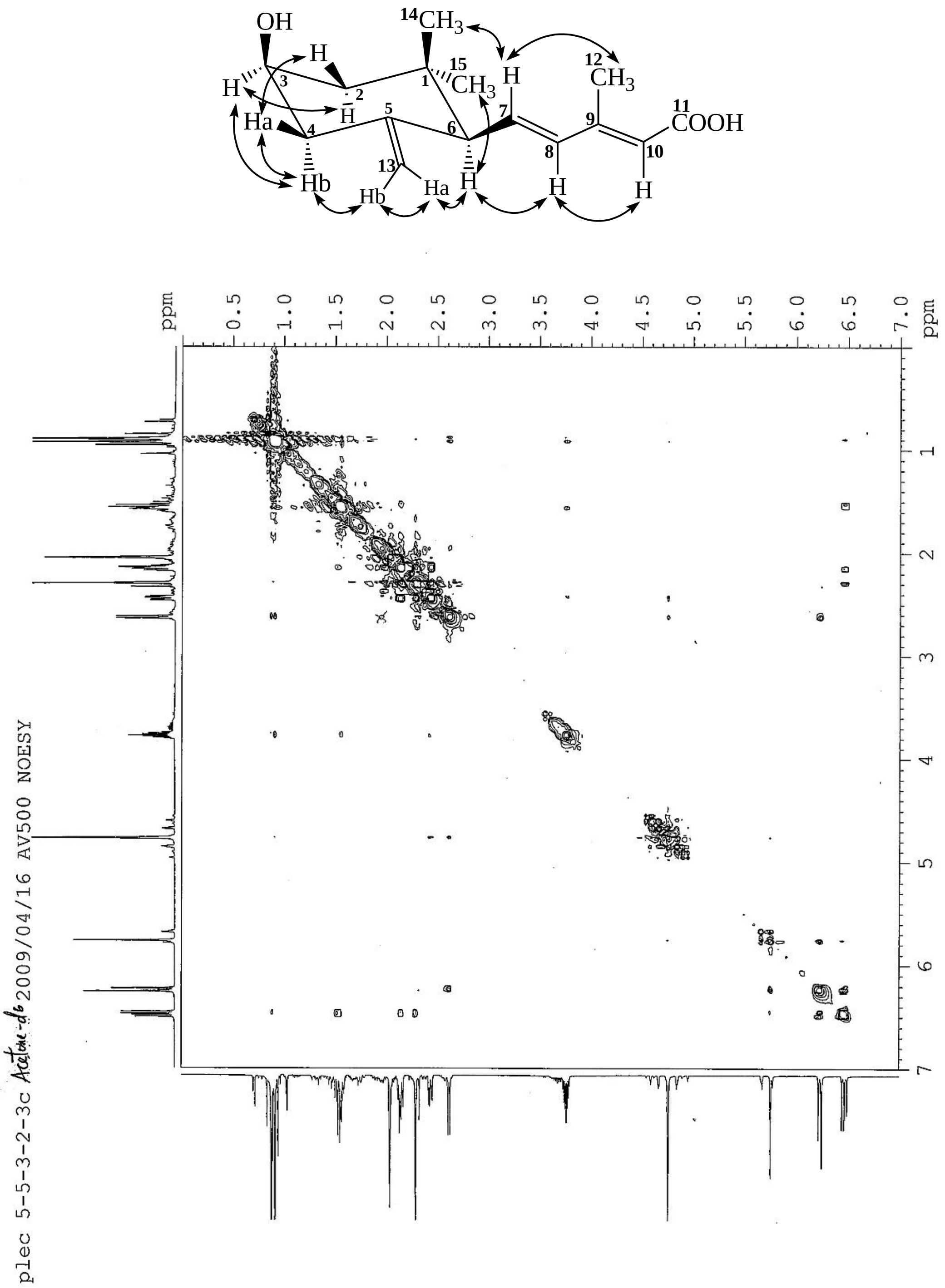

Fig. S12. NOESY spectrum of **6**

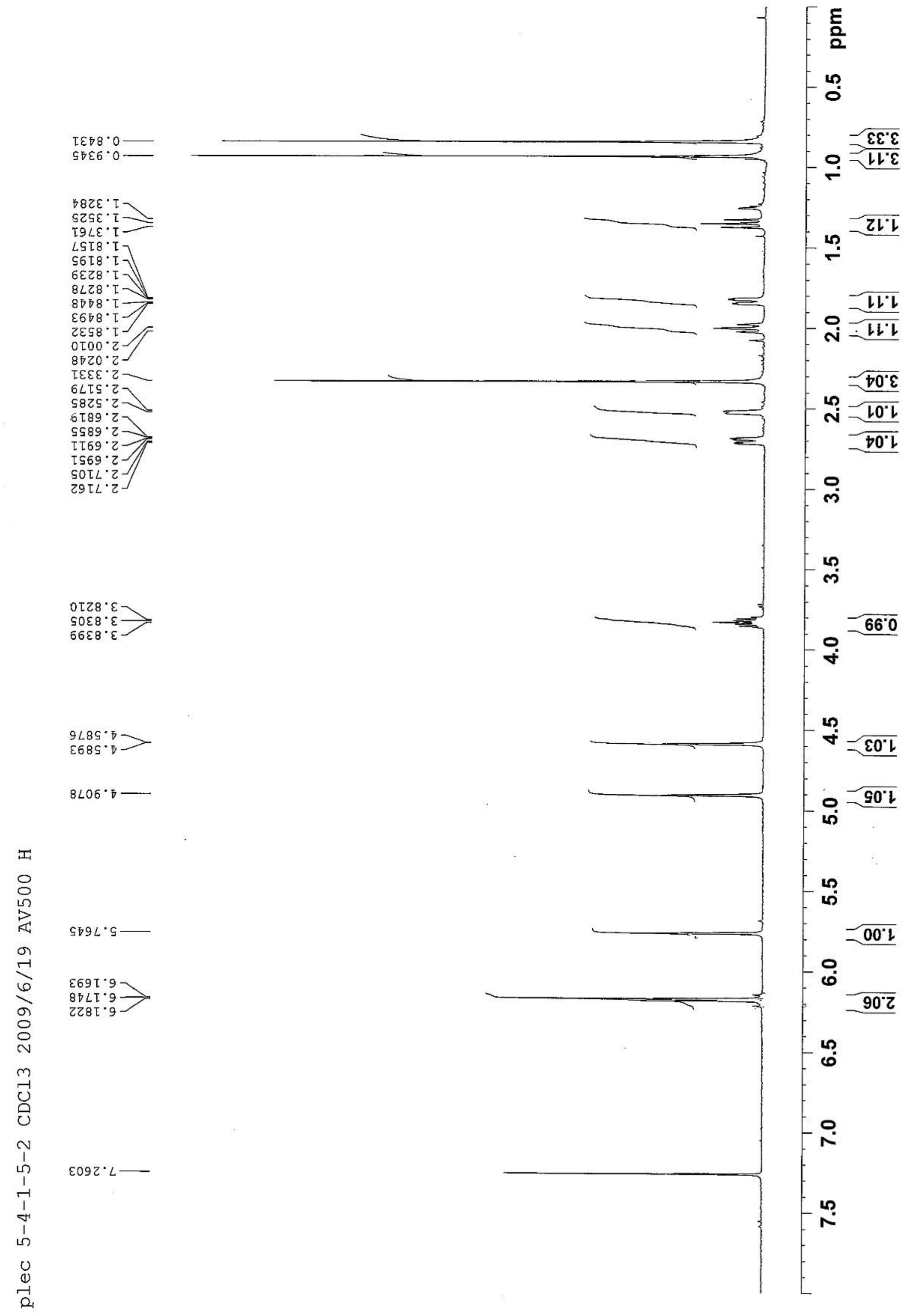

Fig. S13. ^{1}H NMR spectrum of **7**

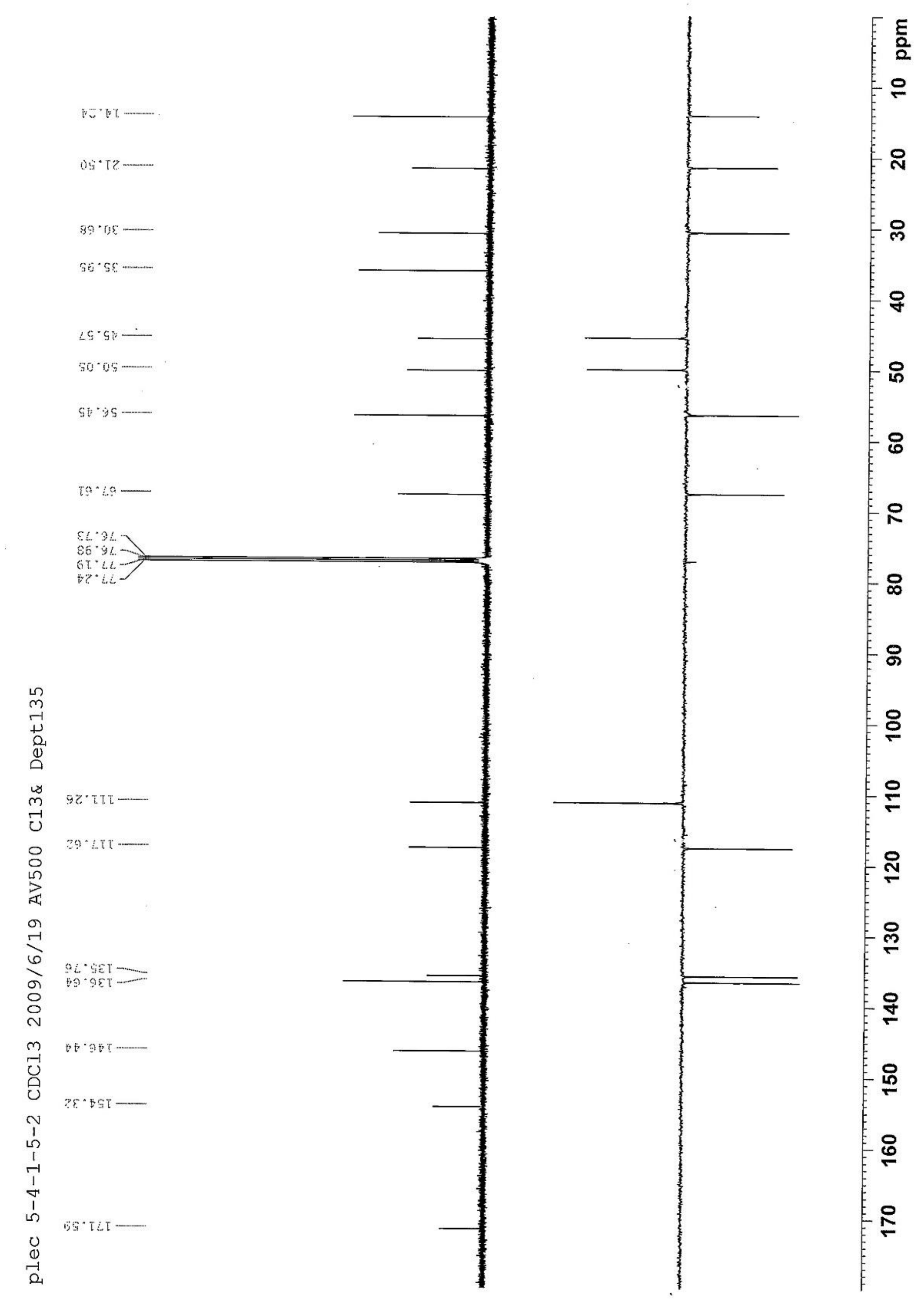

Fig. S14. ^{13}C NMR spectrum of **7**

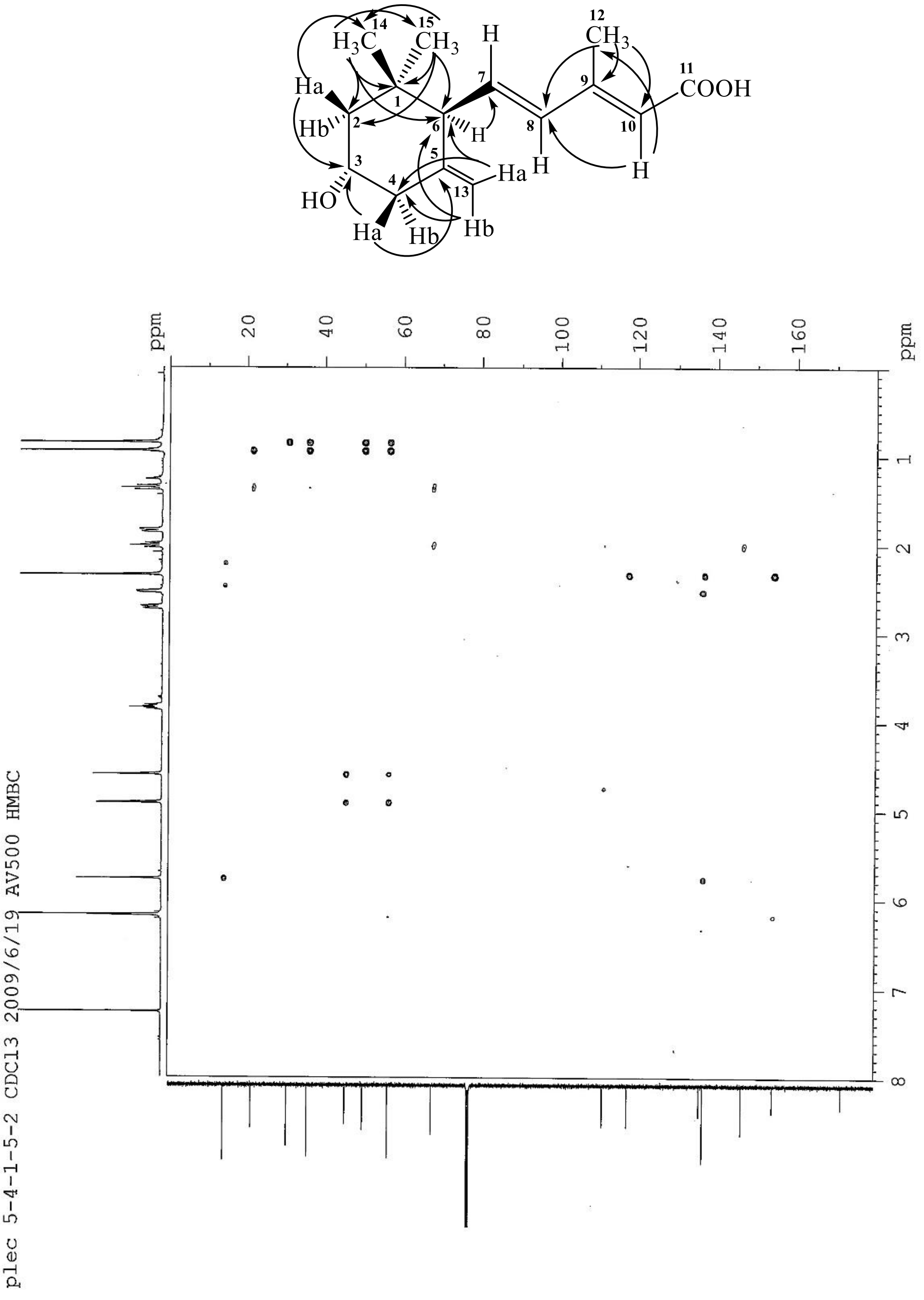

Fig. S15. HMBC spectrum of **7**

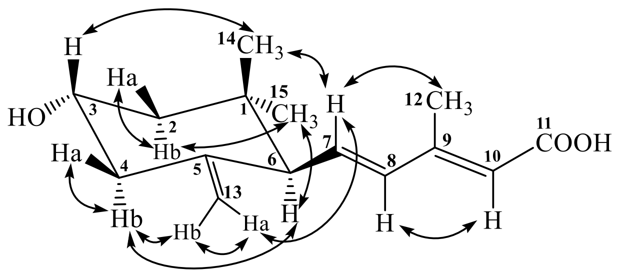

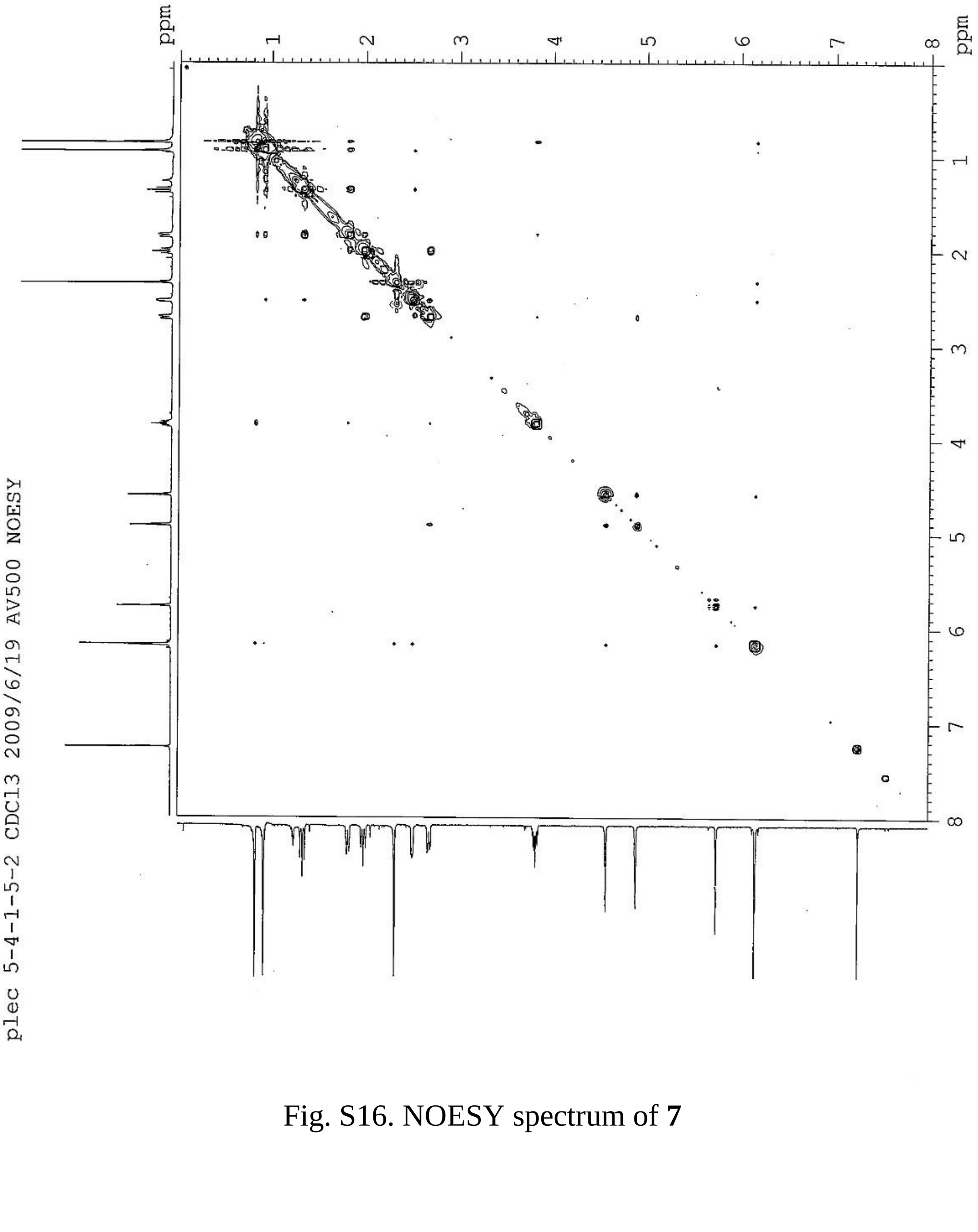

Fig. S16. NOESY spectrum of **7**

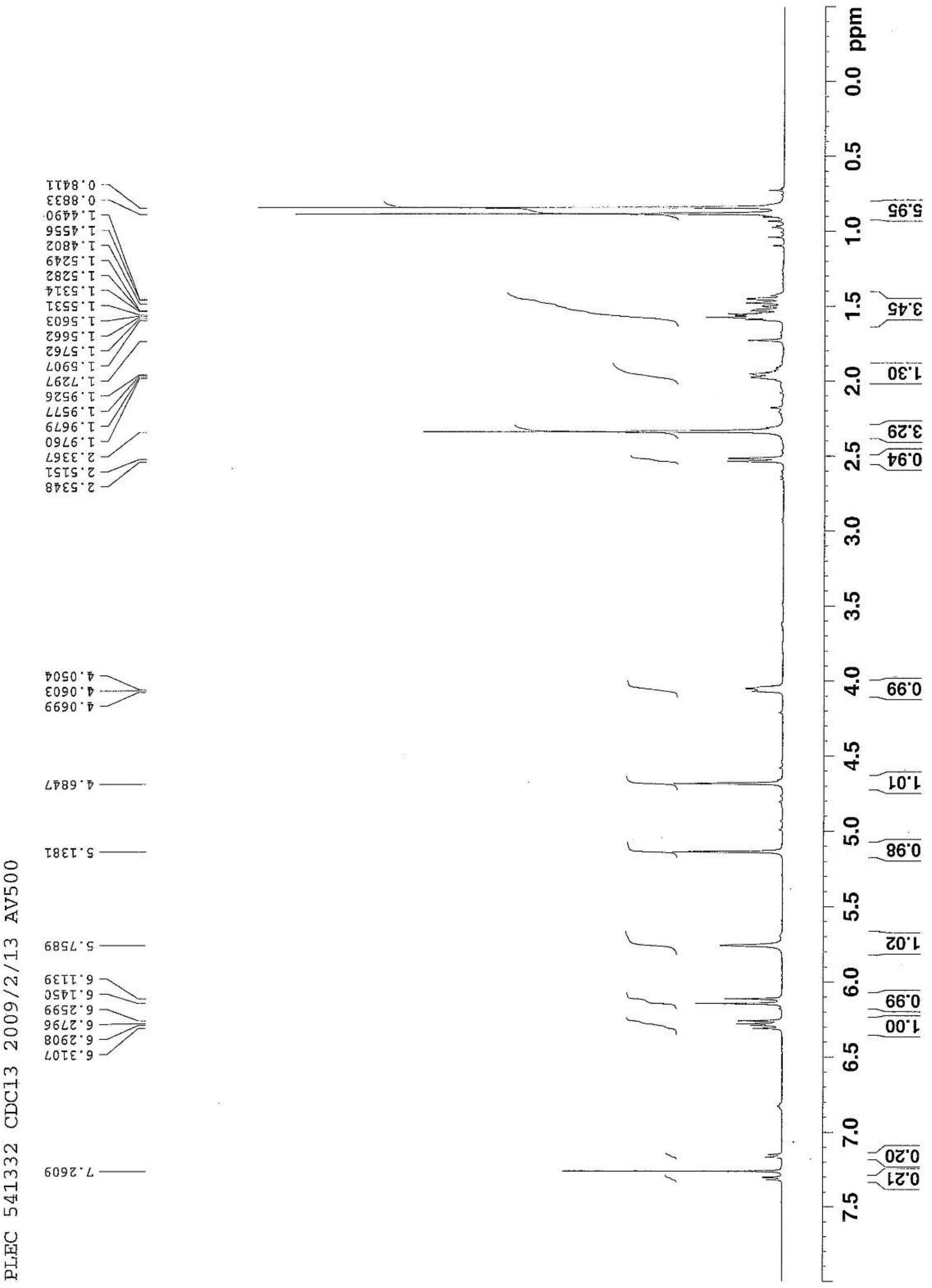

Fig. S17. 1H NMR spectrum of **8**

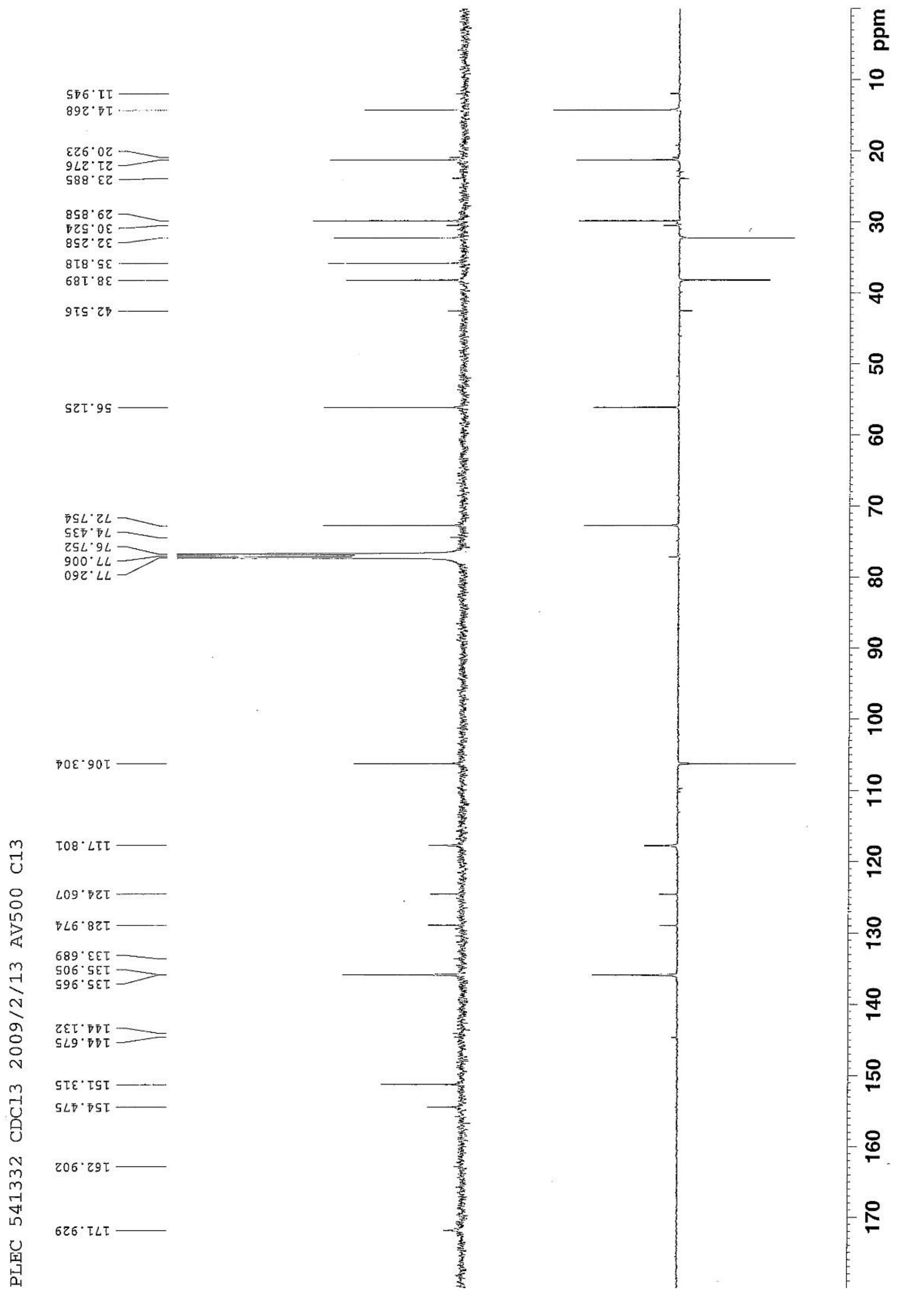

Fig. S18. ^{13}C NMR spectrum of **8**

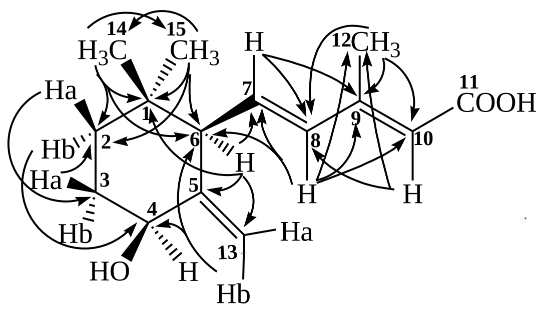

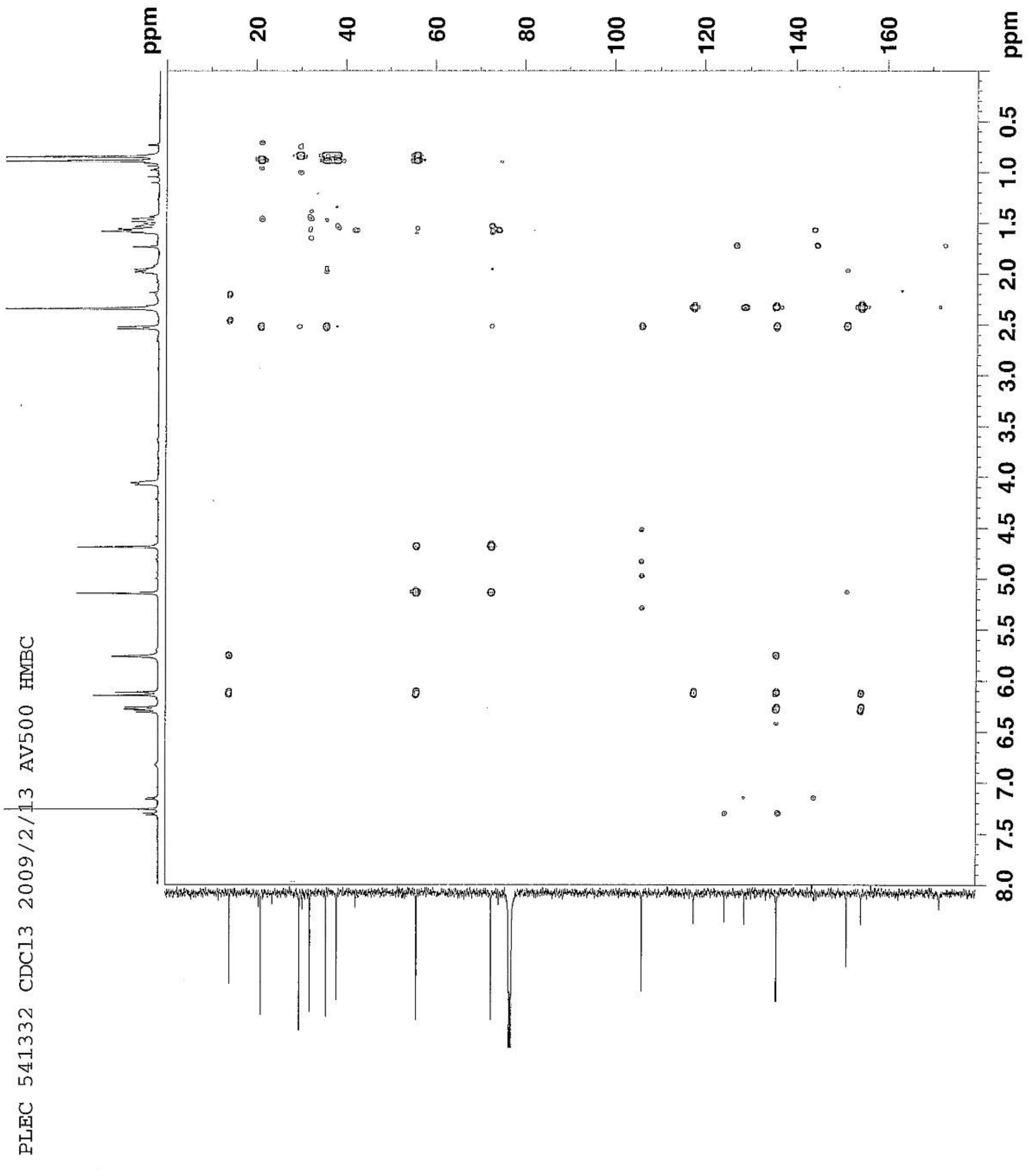

Fig. S19. HMBC spectrum of **8**

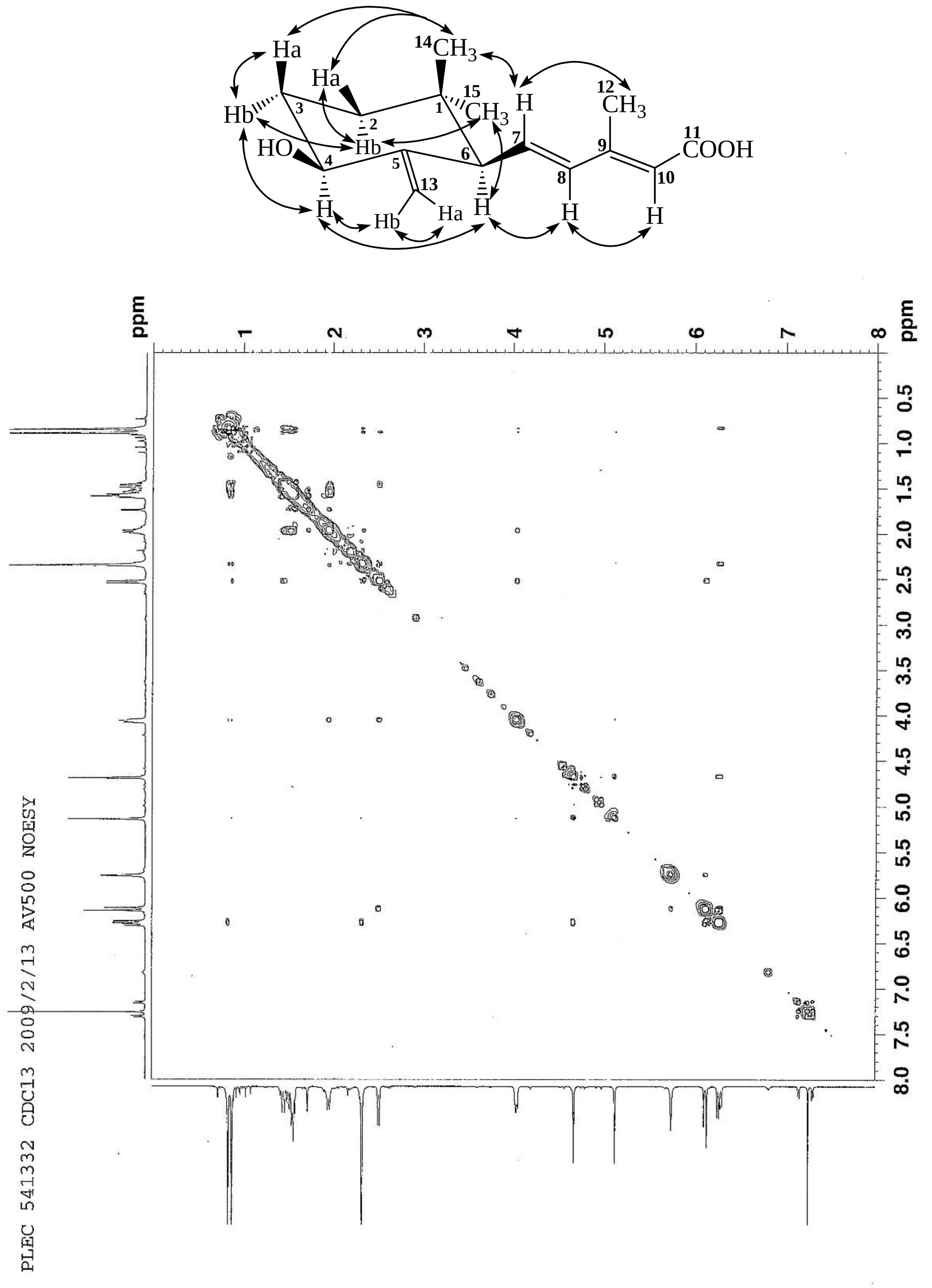

Fig. S20. NOESY spectrum of **8**

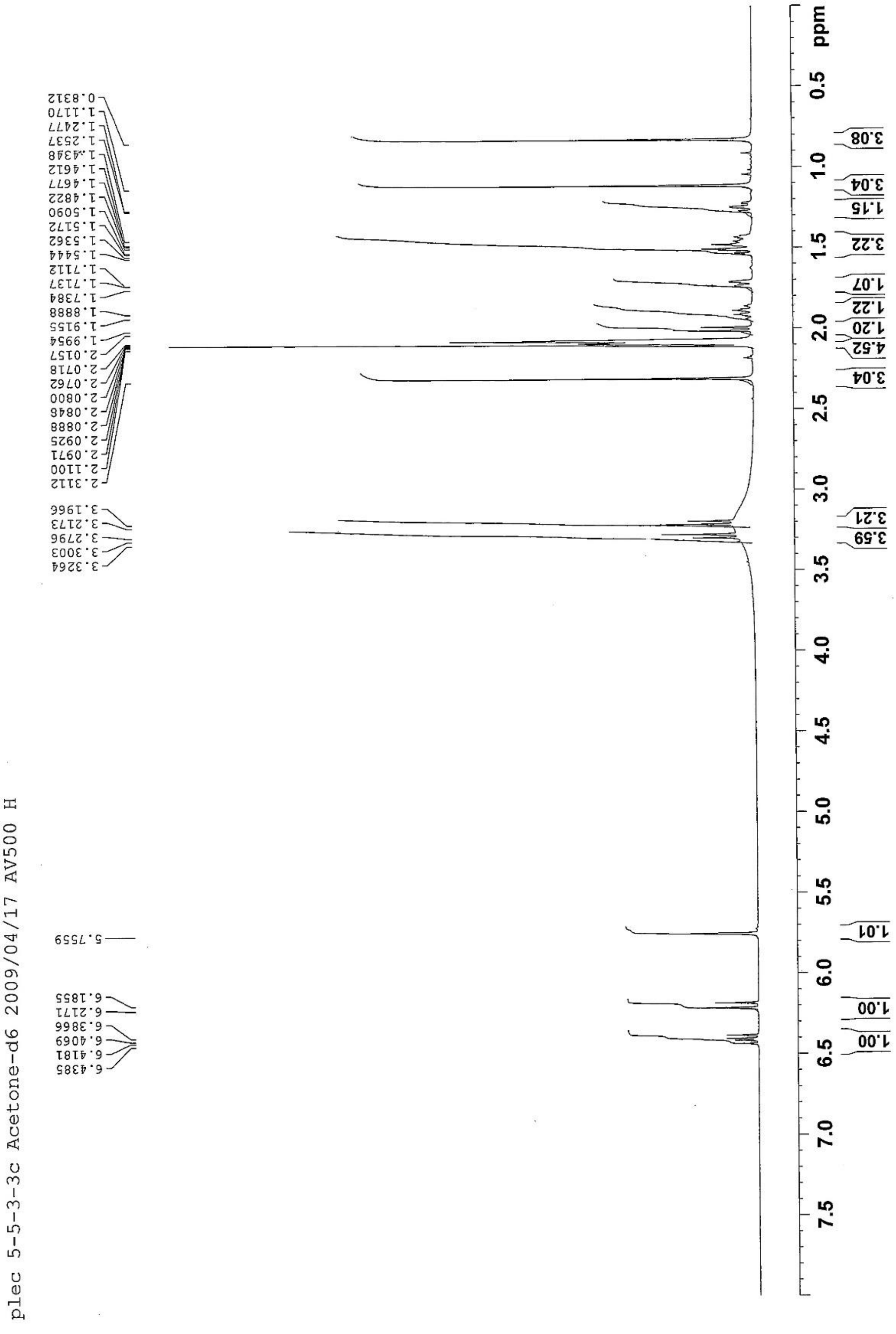

Fig. S21. ^{1}H NMR spectrum of **9**

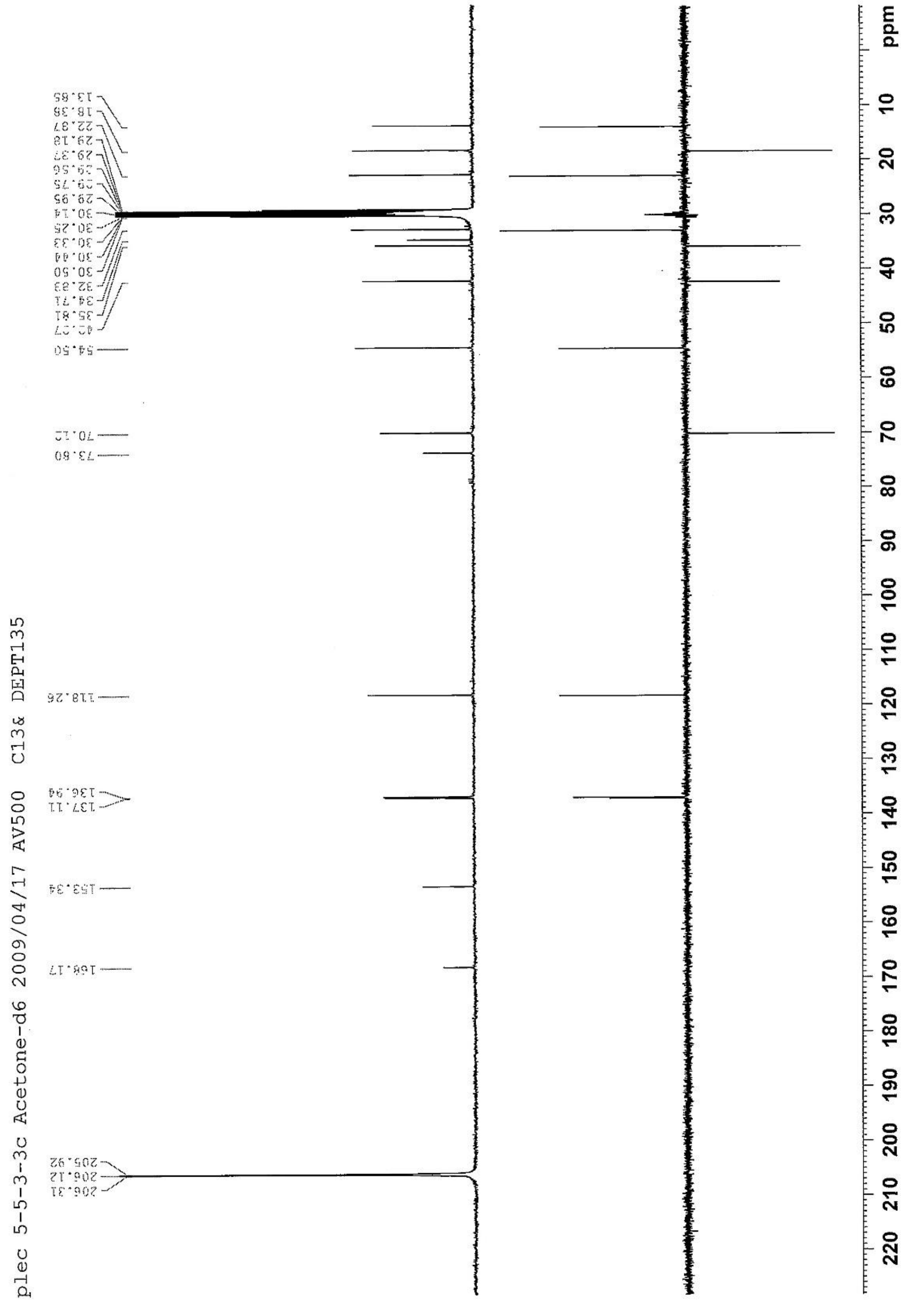

Fig. S22. ^{13}C NMR spectrum of **9**

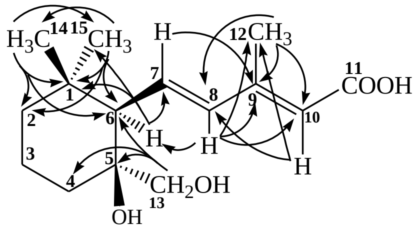

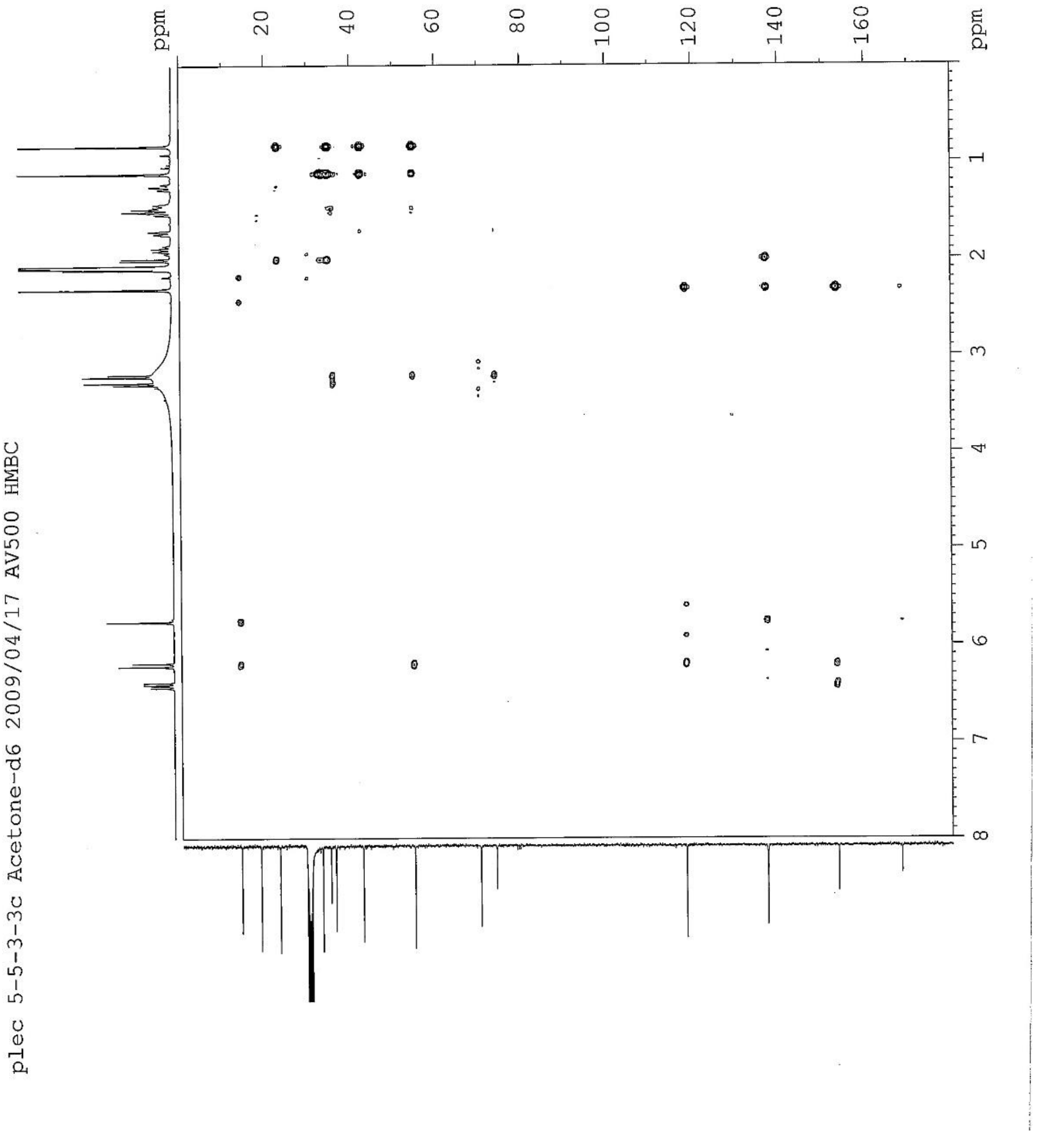

Fig. S23. HMBC spectrum of **9**

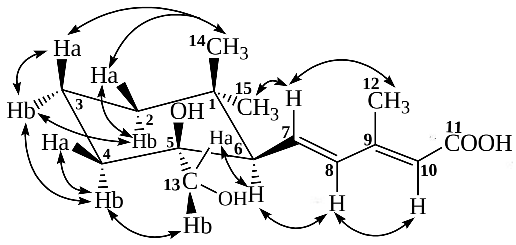

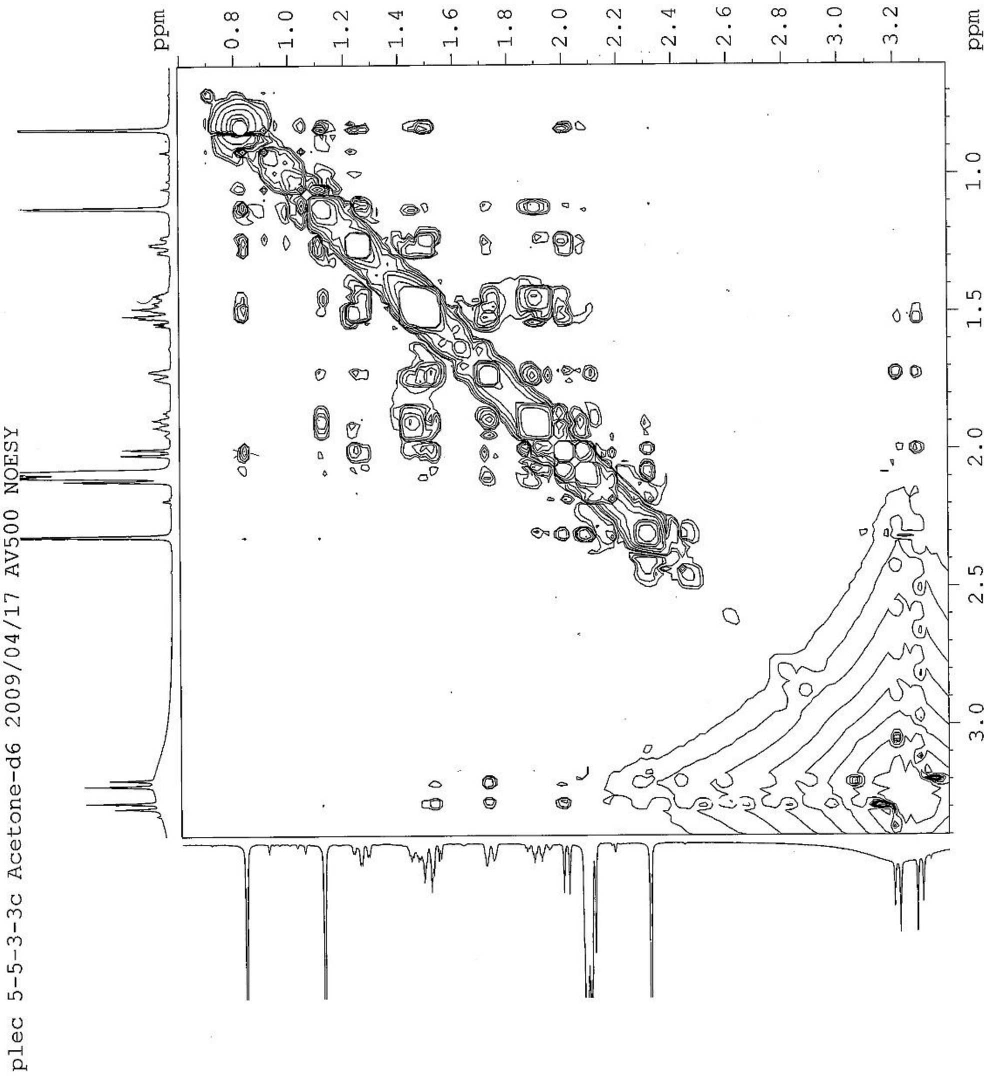

Fig. S24. NOESY spectrum of **9**

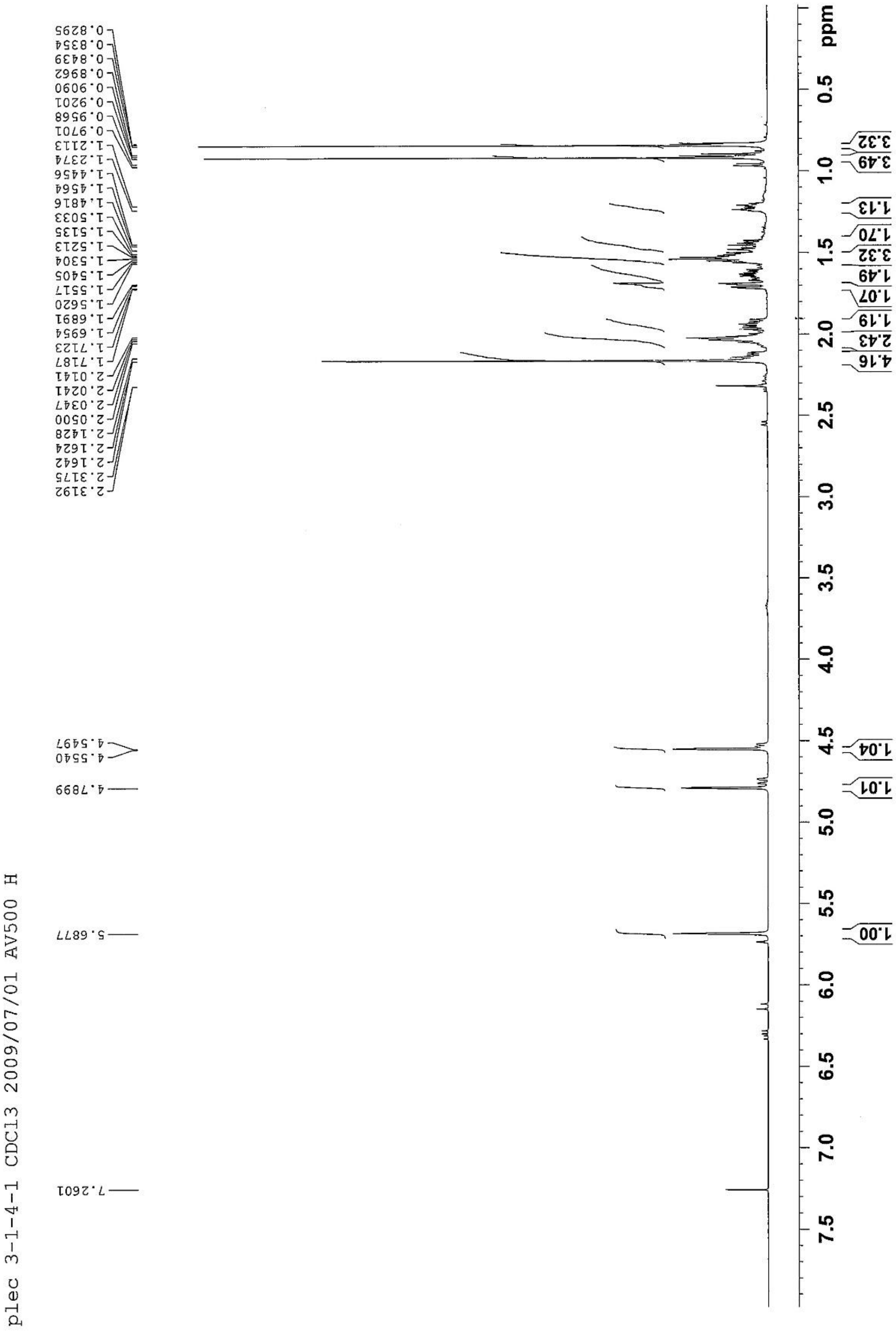

Fig. S25. ^{1}H NMR spectrum of **10**

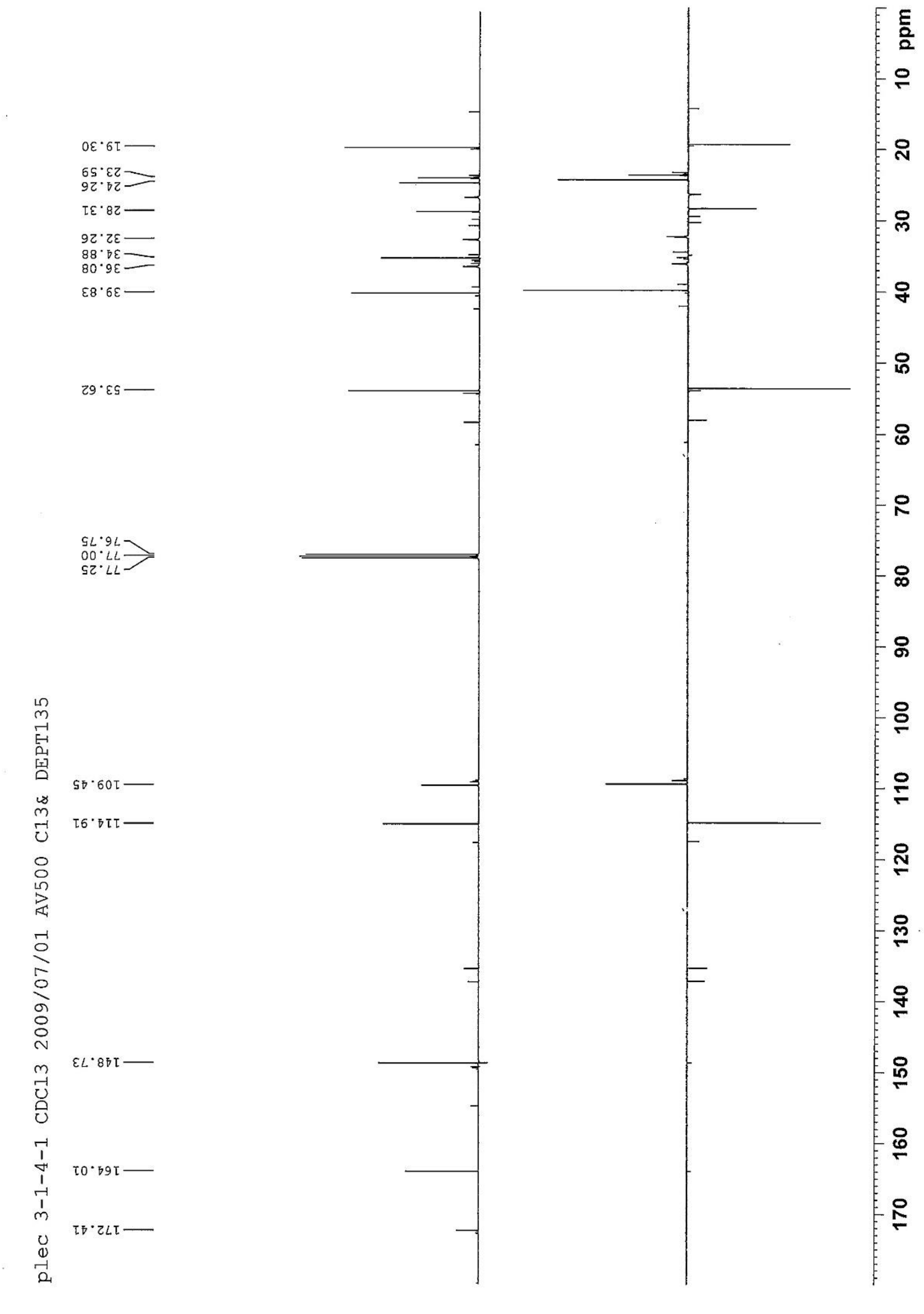

Fig. S26. ^{13}C NMR spectrum of **10**

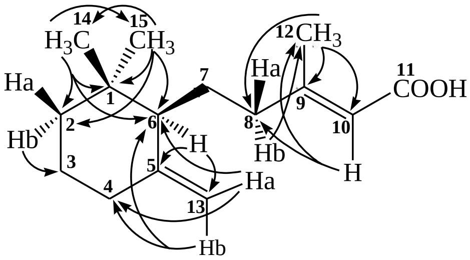

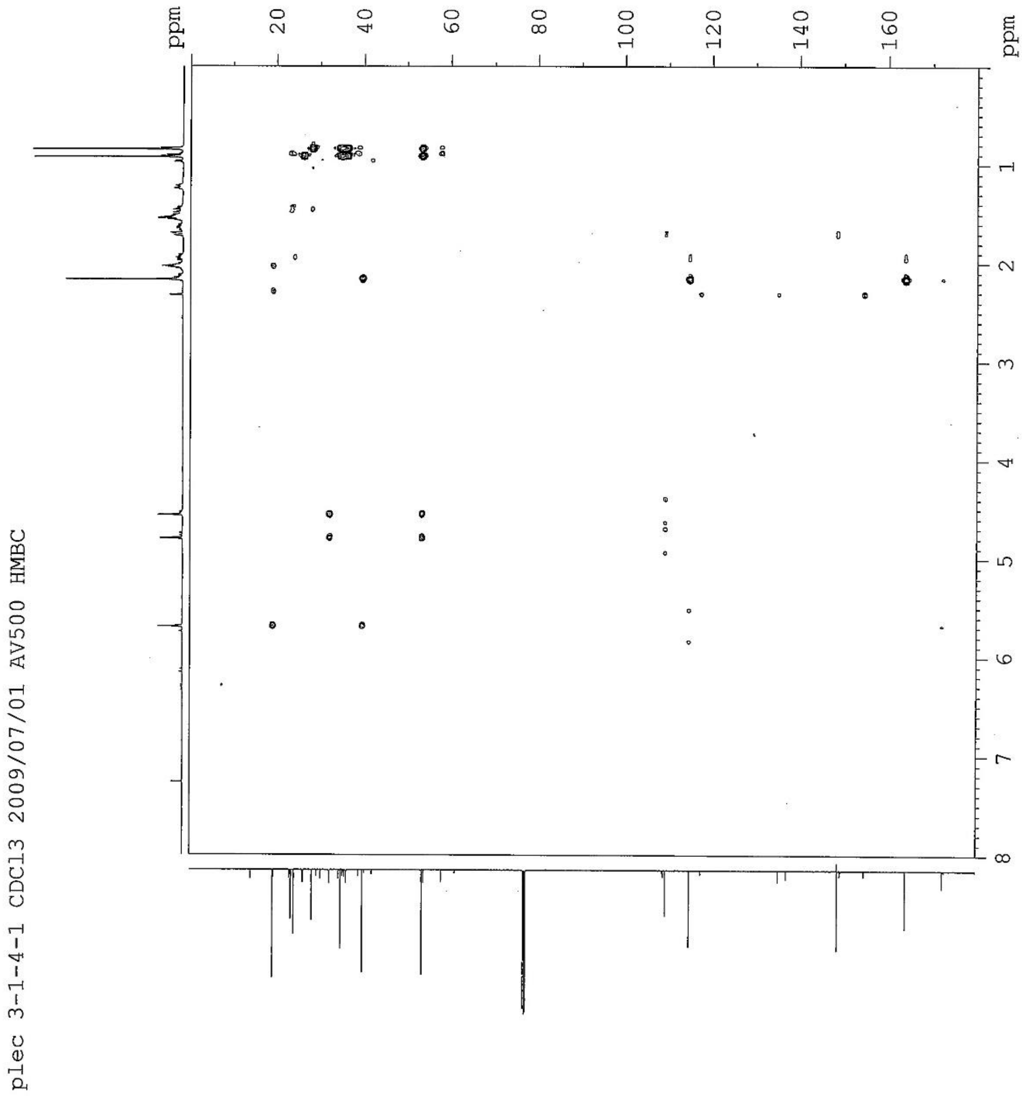

Fig. S27. HMBC spectrum of **10**

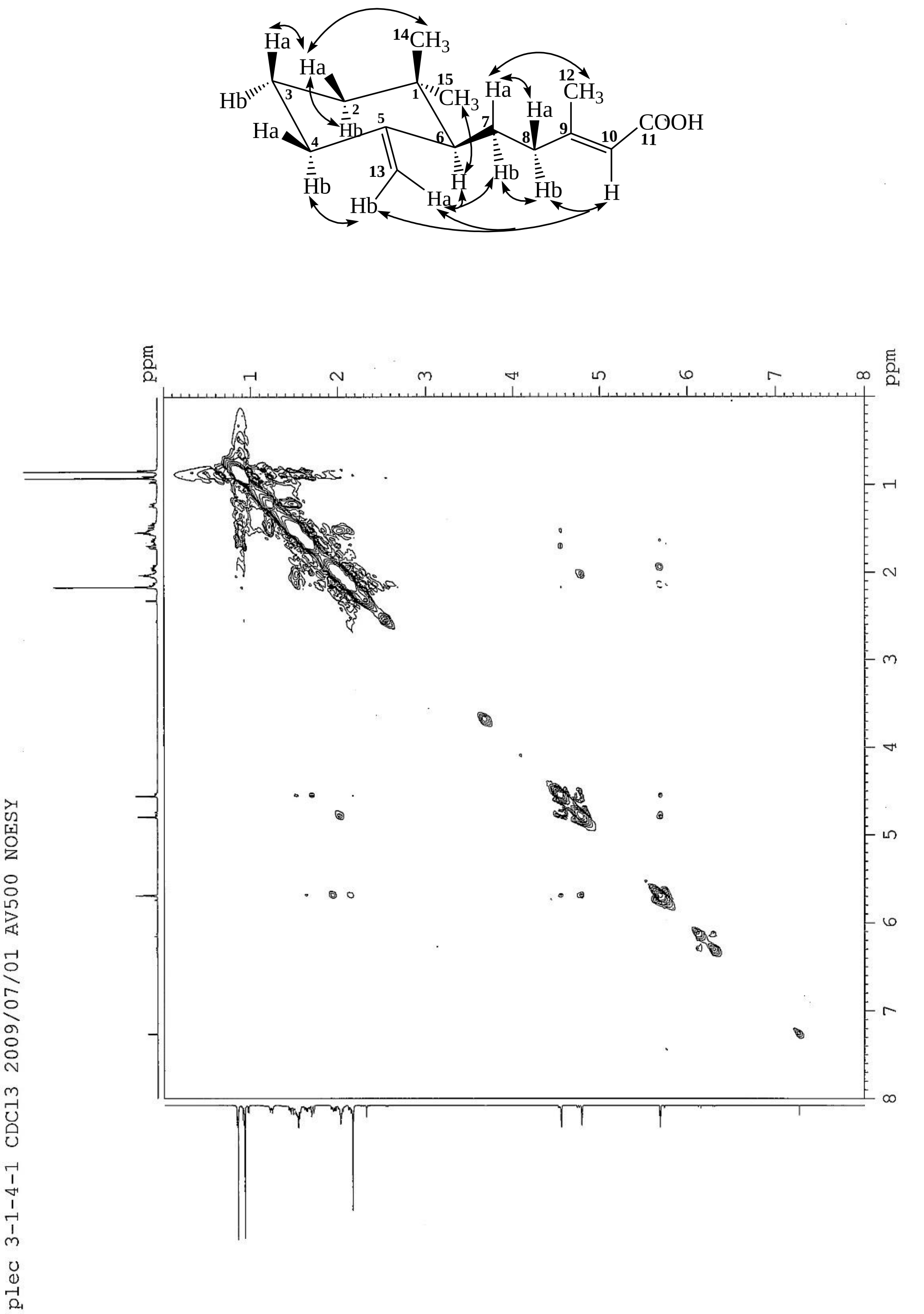

Fig. S28. NOESY spectrum of **10**

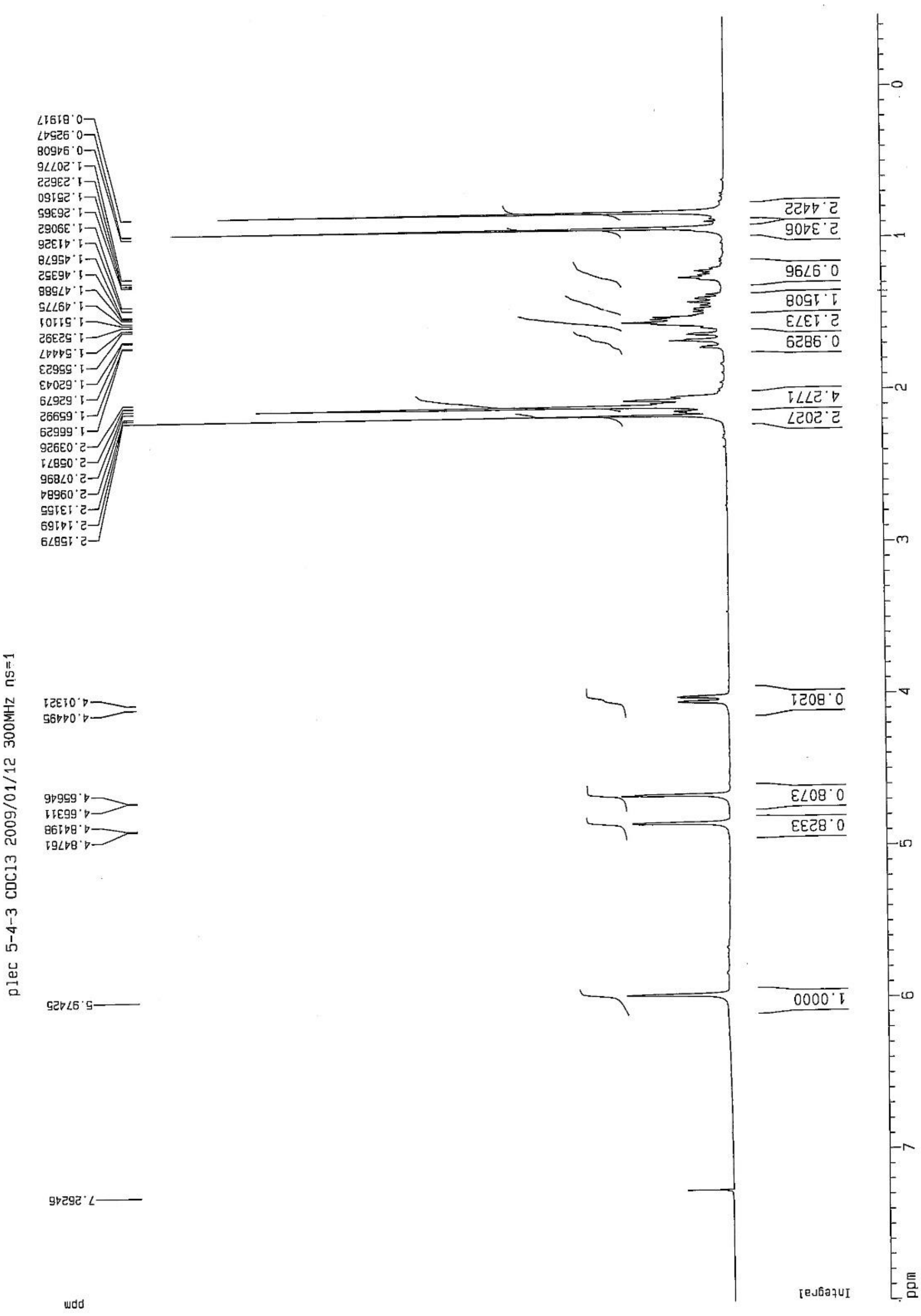

Fig. S29. ^{1}H NMR spectrum of **11**

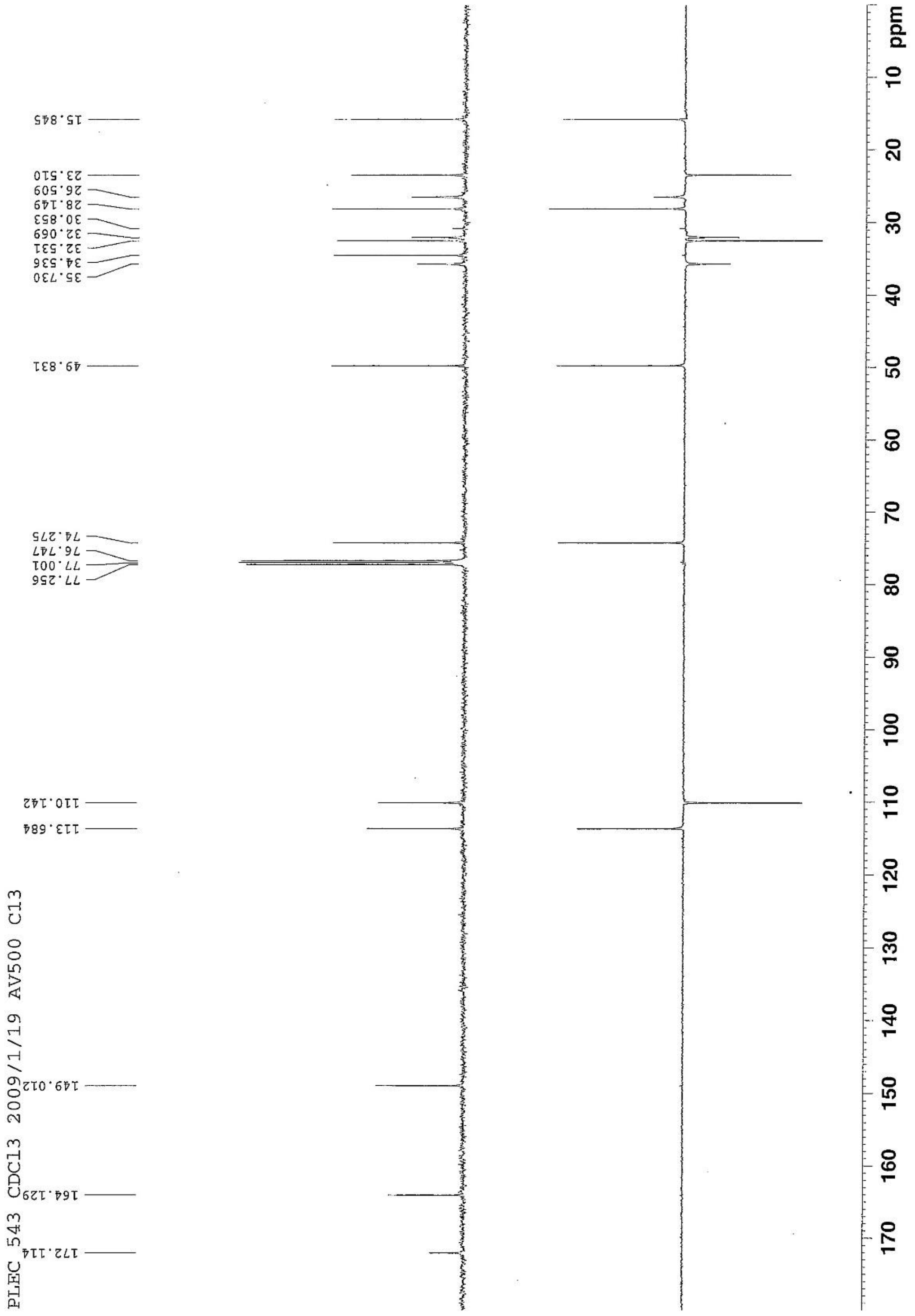

Fig. S30. ^{13}C NMR spectrum of **11**

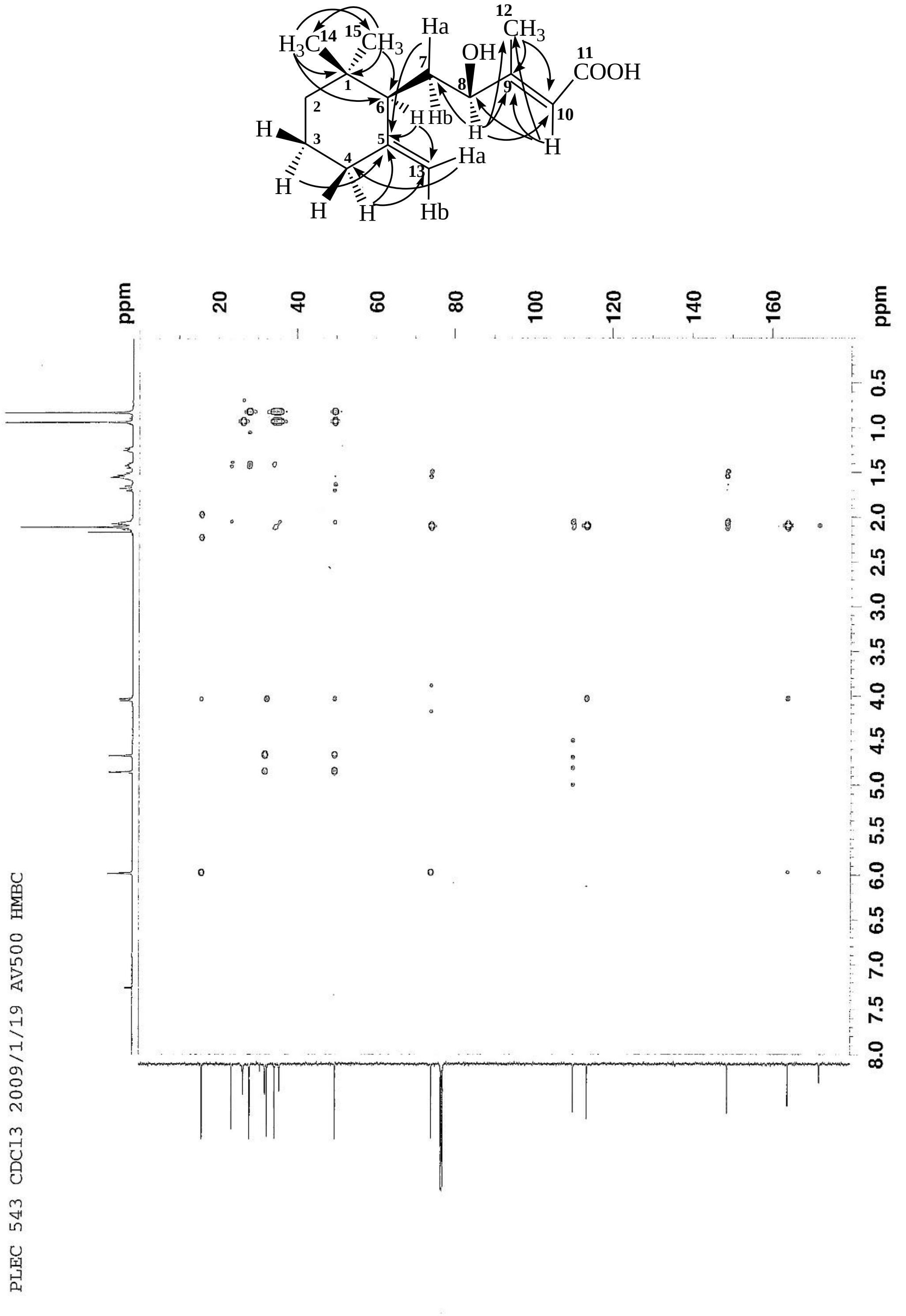

Fig. S31. HMBC spectrum of **11**

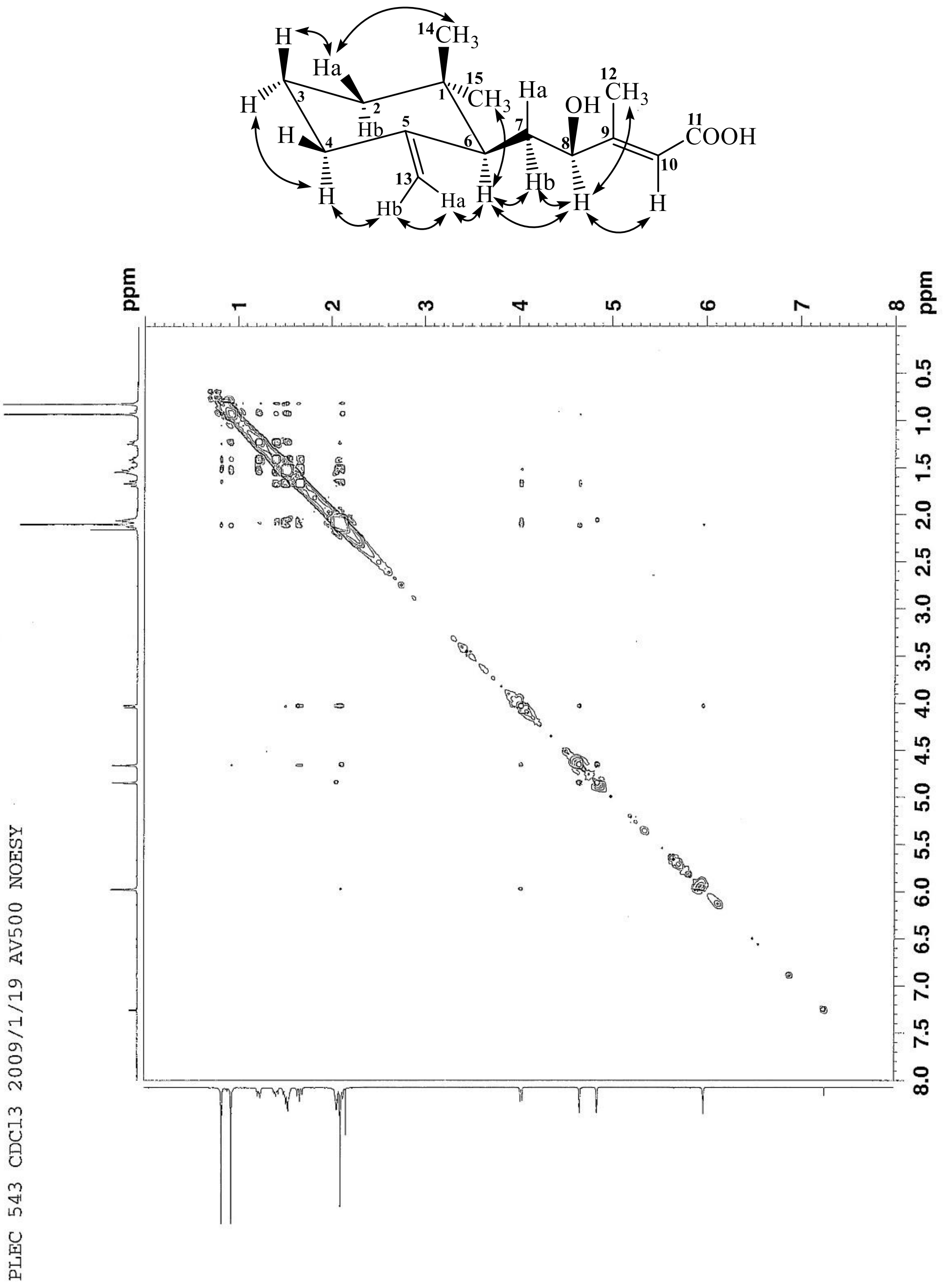

Fig. S32. NOESY spectrum of **11**

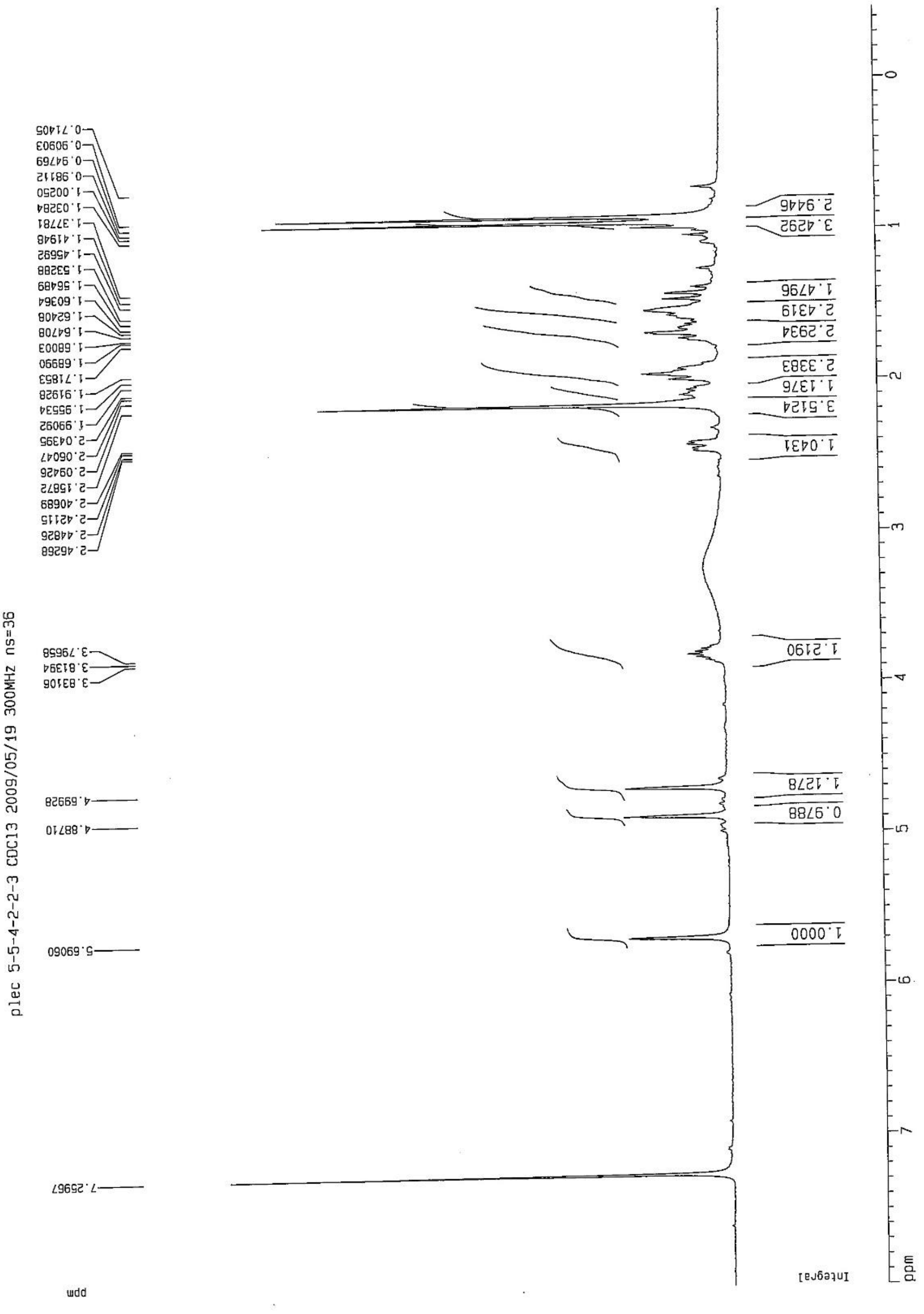

Fig. S33. ^{1}H NMR spectrum of **12**

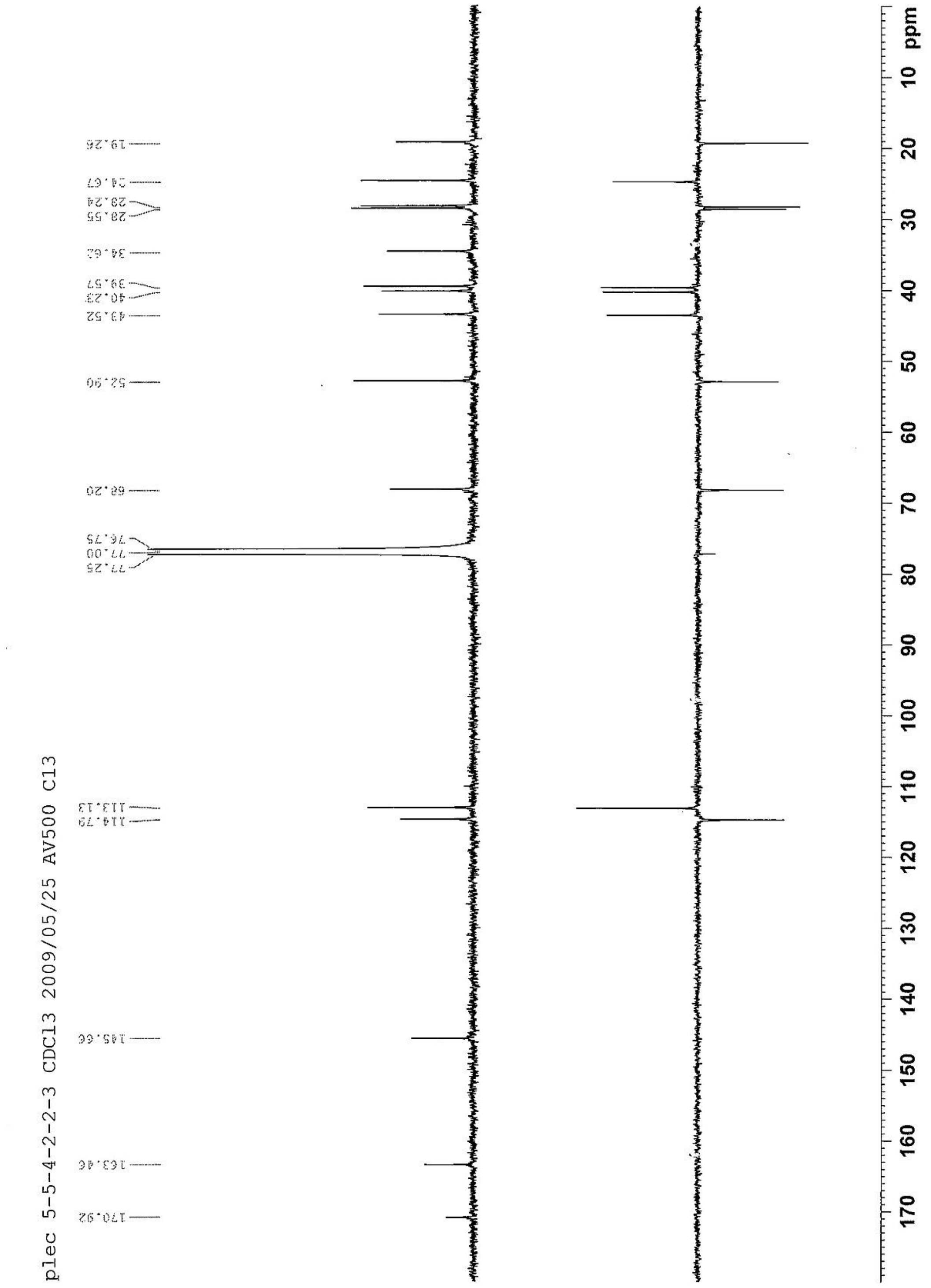

Fig. S34. ^{13}C NMR spectrum of **12**

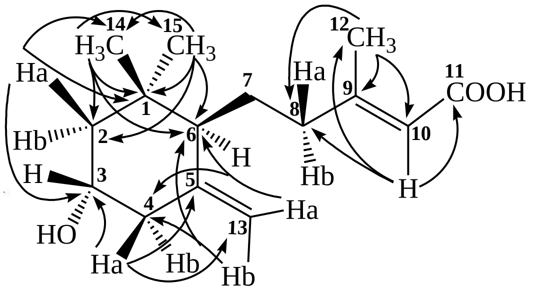

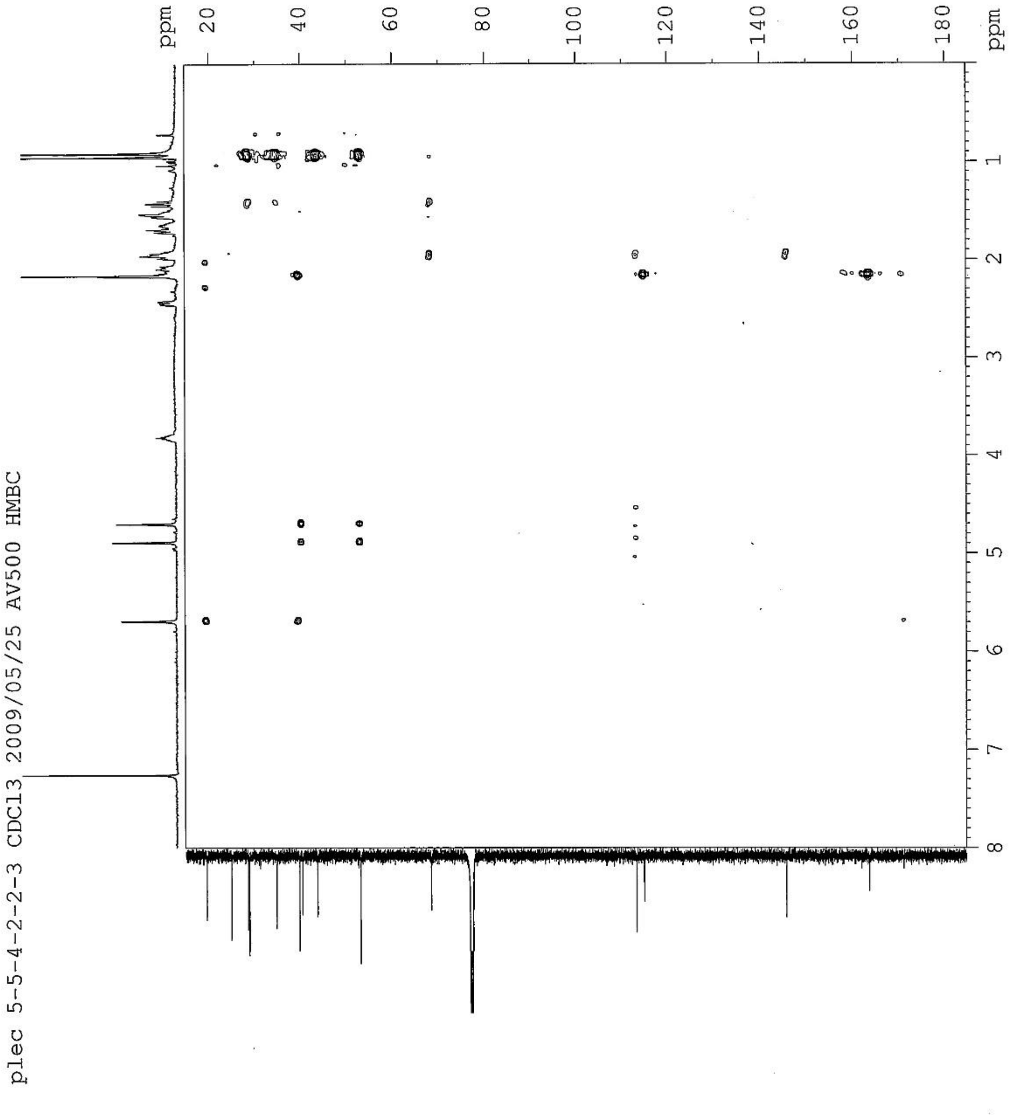

Fig. S35. HMBC spectrum of **12**

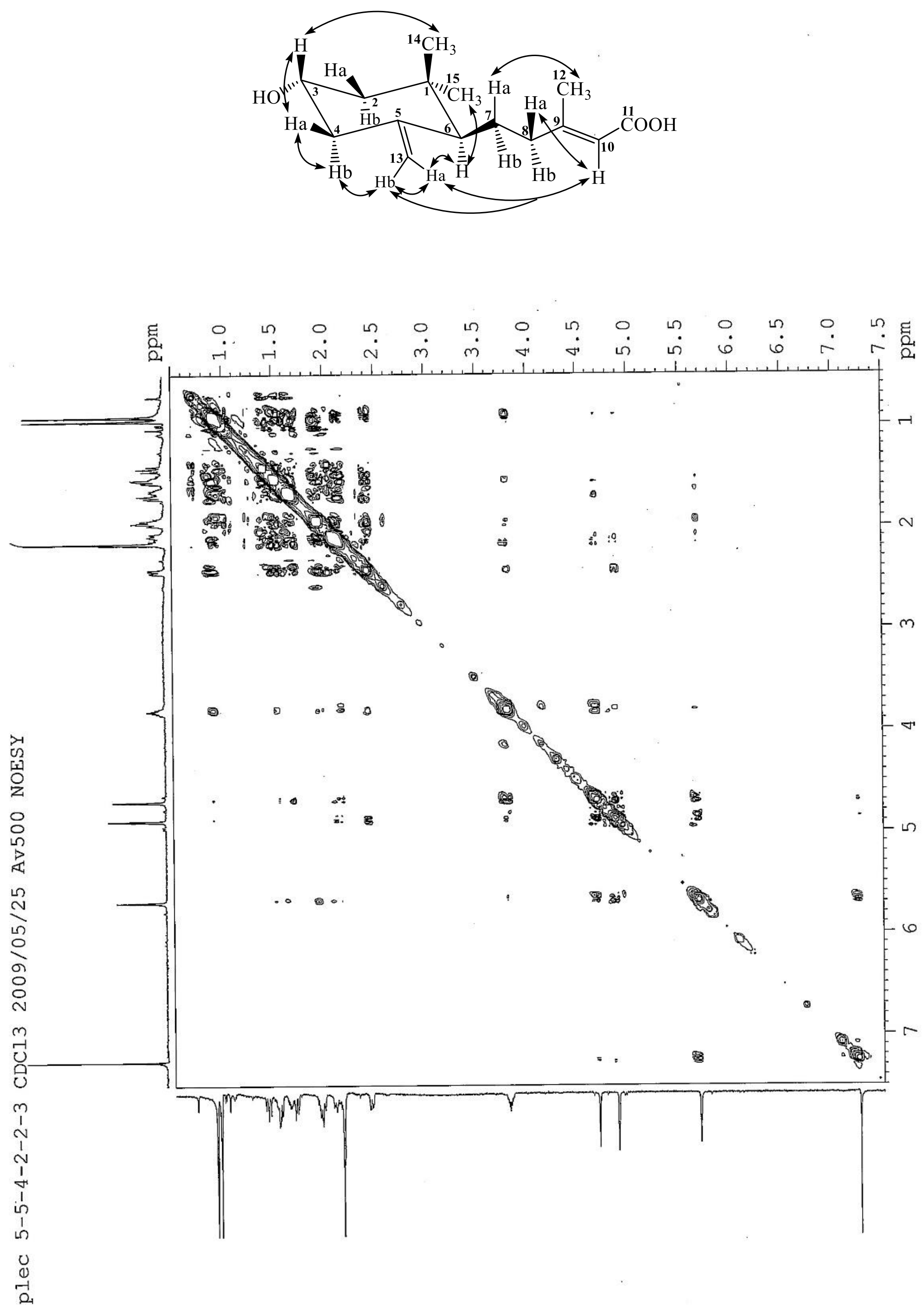

Fig. S36. NOESY spectrum of **12**

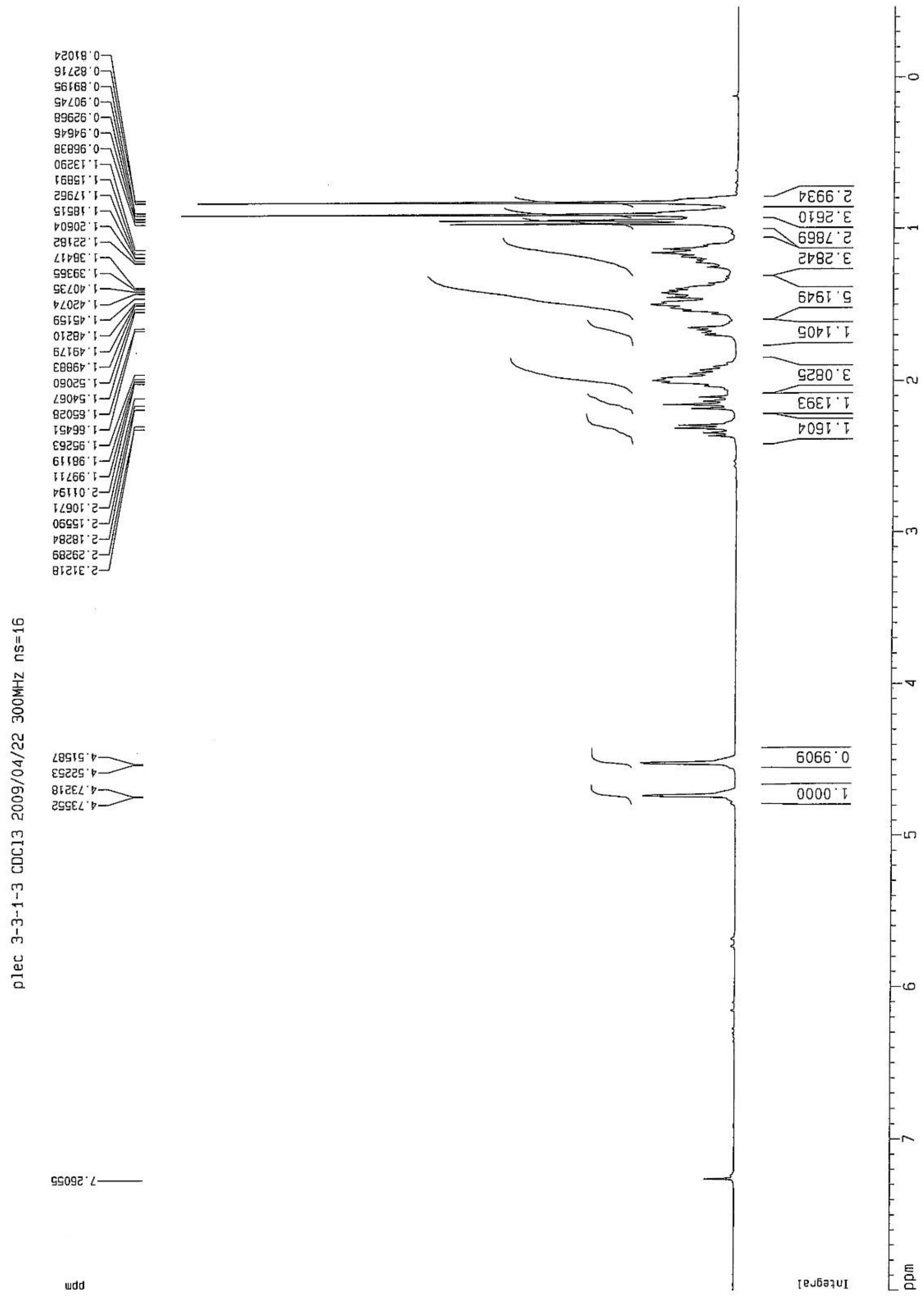

Fig. S37. ^{1}H NMR spectrum of **13**

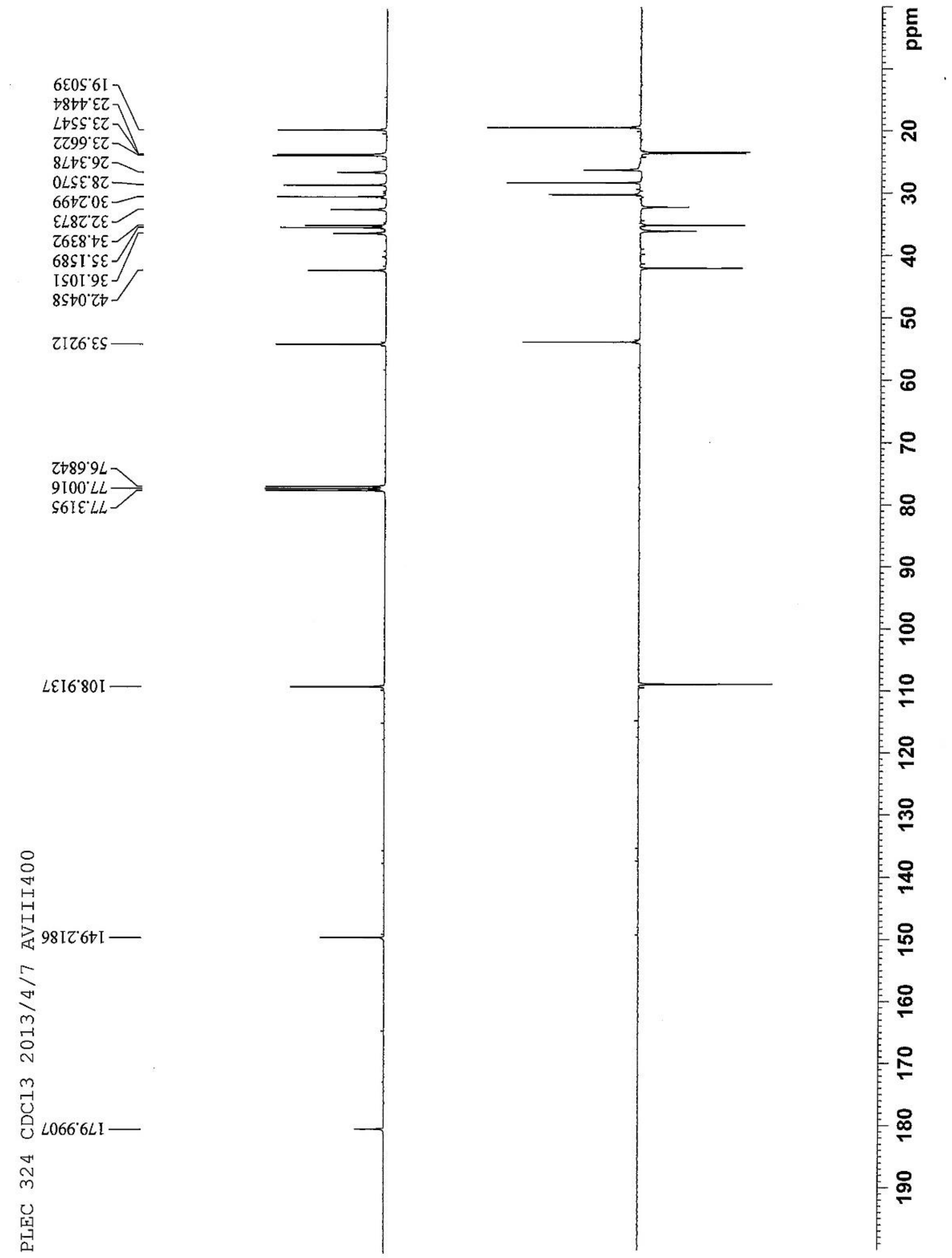

Fig. S38. ^{13}C NMR spectrum of **13**

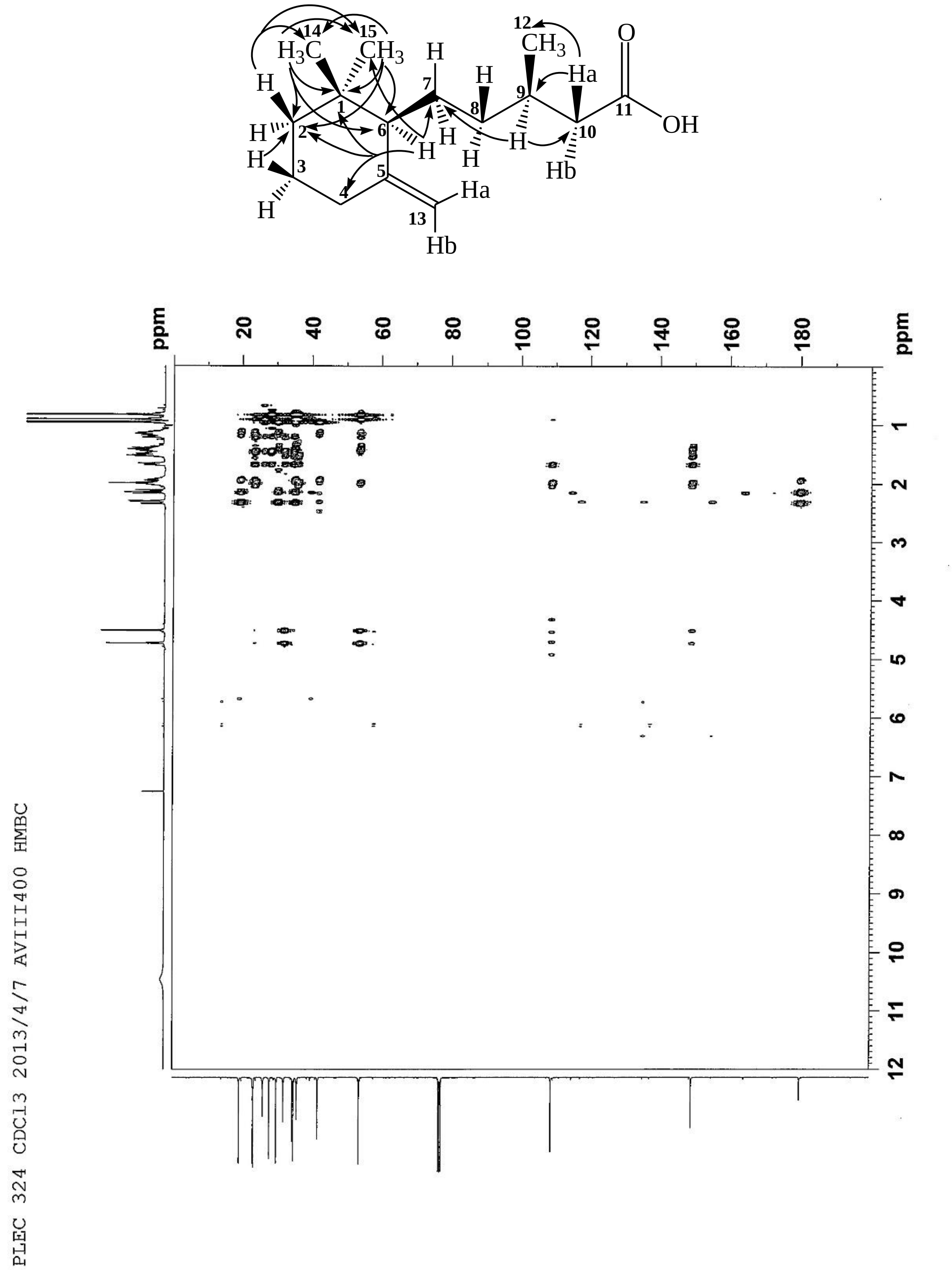

Fig. S39. HMBC spectrum of **13**

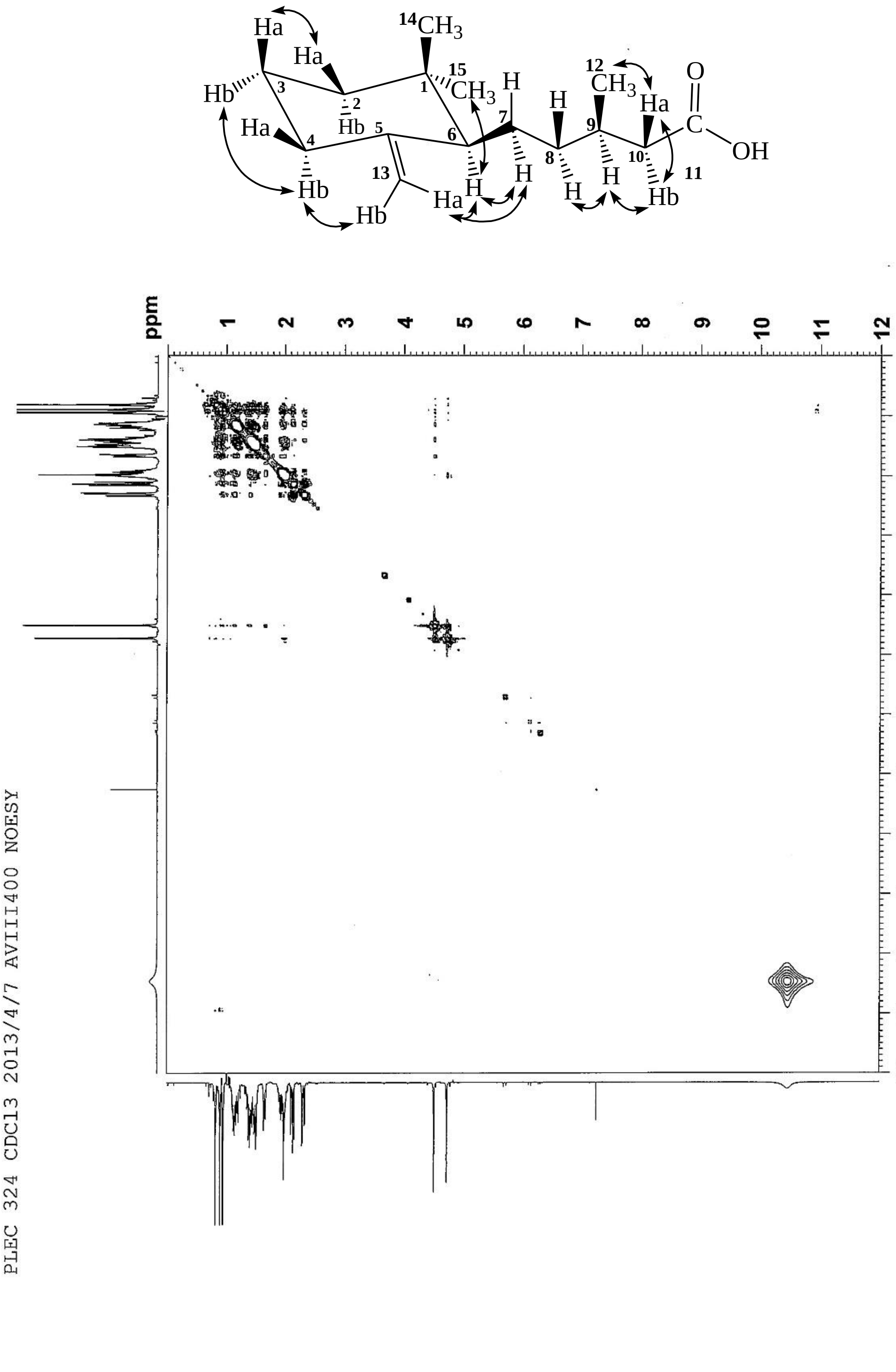

Fig. S40. NOESY spectrum of **13**

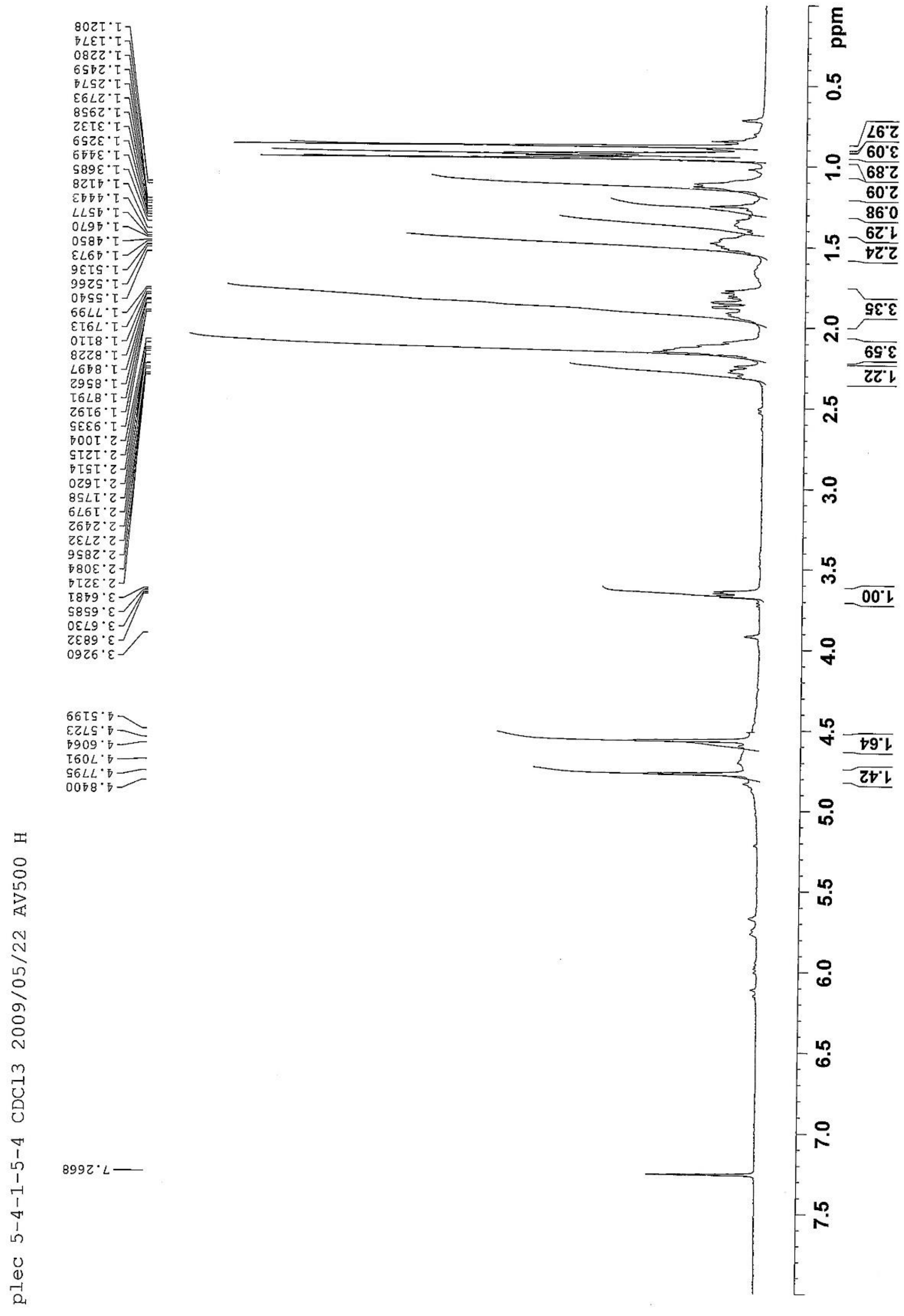

Fig. S41. ¹H NMR spectrum of **14**

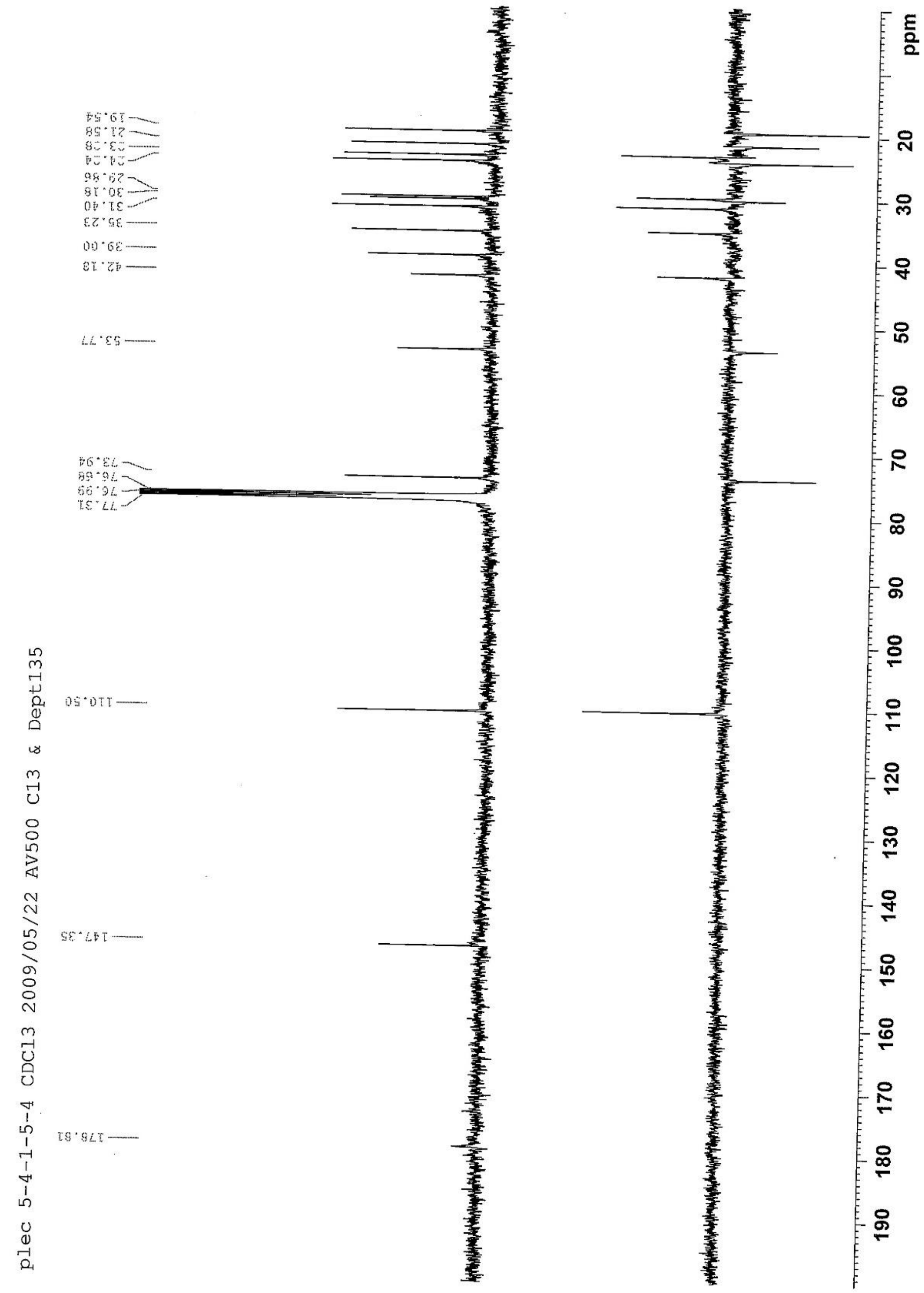

Fig. S42. ^{13}C NMR spectrum of **14**

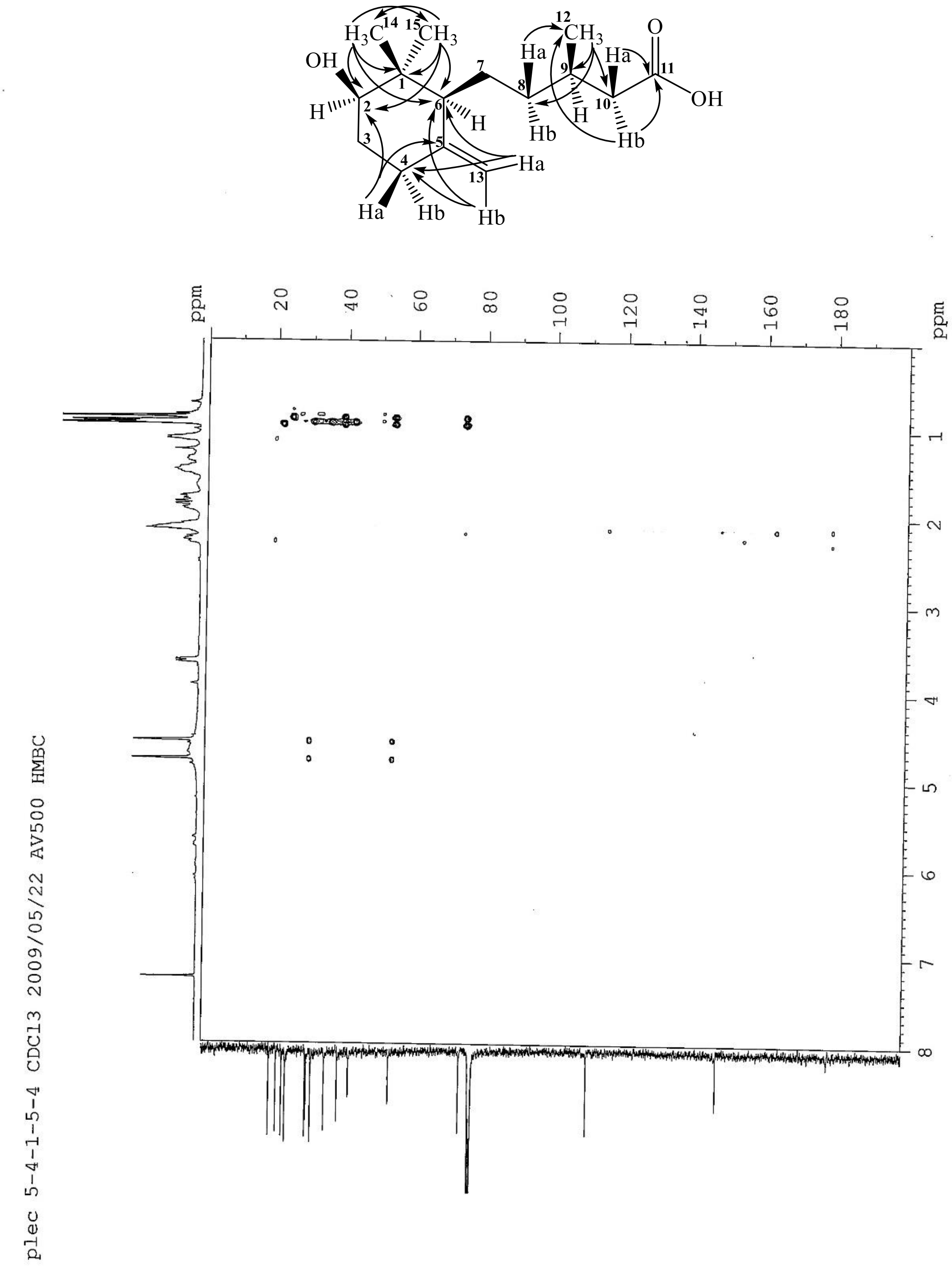

Fig. S43. HMBC spectrum of **14**

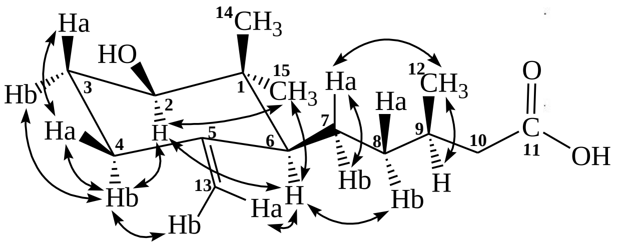

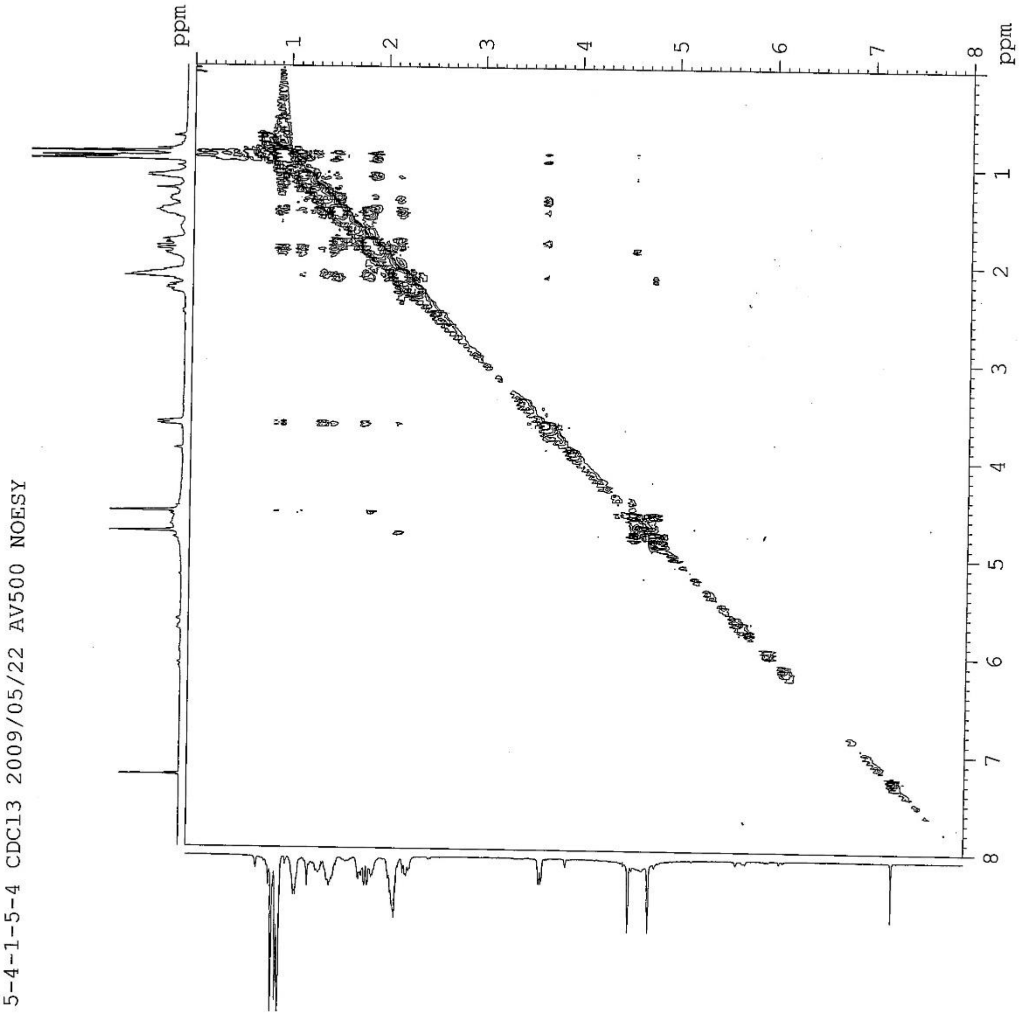

Fig. S44. NOESY spectrum of **14**

Fig. S45. Western blot analysis of examined compounds.

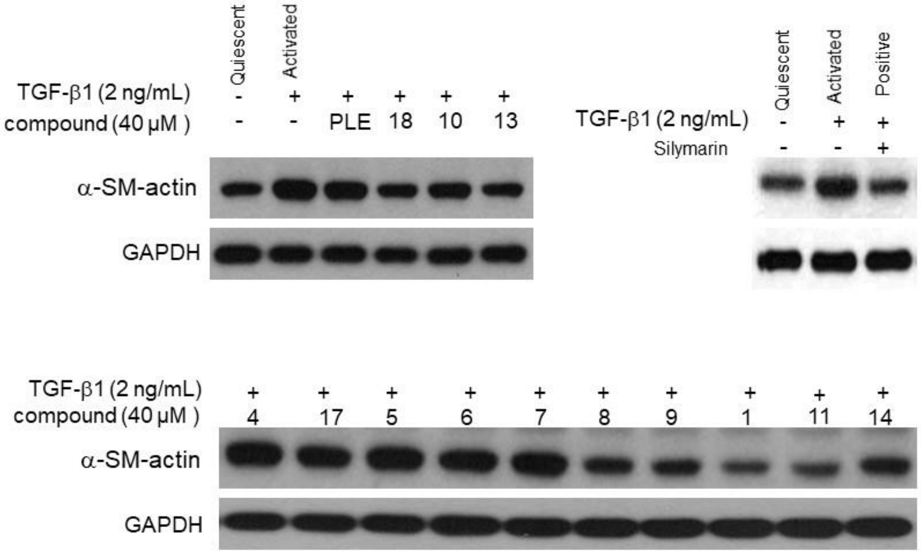

Experimental detail of western blot analysis

Proteins were separated on 10 % denatured gels and transferred to PVDF membranes. The transferred membranes were then incubated for 1 h with blocking solution of 5 % nonfat milk-TBST solution, followed by immersion in the same solution containing an antibody against glyceraldehyde 3-phosphate dehydrogenase (GAPDH) and α-SMA (Santa Cruz) overnight. After washing with the mixture of tris-buffered saline and Tween® 20 (TBST) four times, the membranes were incubated with the 5 % nonfat milk-TBST solution containing peroxidase-labeled anti-rabbit IgG (Santa Cruz) for 2 h. After washing in TBST five times, enhanced chemiluminescence substrate (ECL, PerkinElmer TM) was used for protein detection. Band intensity was quantified using GeneTools Image Software (Syngene, England), as GAPDH was used as the internal control. The Western blot experiments were repeated in triplicate.
